# Ruthenium Olefin Metathesis Catalysts Bearing a Macrocyclic N‐Heterocyclic Carbene Ligand: Improved Stability and Activity

**DOI:** 10.1002/anie.202201472

**Published:** 2022-04-13

**Authors:** Wioletta Kośnik, Dawid Lichosyt, Marcin Śnieżek, Angelika Janaszkiewicz, Krzysztof Woźniak, Maura Malińska, Bartosz Trzaskowski, Anna Kajetanowicz, Karol Grela

**Affiliations:** ^1^ Biological and Chemical Research Centre Faculty of Chemistry University of Warsaw Żwirki i Wigury Street 101 02-089 Warsaw Poland; ^2^ Institute of Organic Chemistry Polish Academy of Sciences Kasprzaka 44/52 01-224 Warsaw Poland; ^3^ Centre of New Technologies University of Warsaw Banacha 2c 02-097 Warsaw Poland

**Keywords:** Catalyst Design, N-Heterocyclic Carbenes, Olefin Metathesis, Reaction Mechanisms, Ruthenium

## Abstract

Formation of sterically hindered C−C double bonds *via* catalytic olefin metathesis is considered a very challenging task for Ru catalysts. This limitation led to the development of specialised catalysts bearing sterically reduced N‐heterocyclic carbene (NHC) ligands that are very active in such transformations, yet significantly less stable as compared to general purpose catalysts. To decrease the small‐size NHC catalysts susceptibility to decomposition, a new NHC ligand was designed, in which two sterically reduced aryl arms were tied together by a C‐8 alkyl chain. The installation of this macrocyclic ligand on the ruthenium centre led to the formation of an olefin metathesis catalyst (*trans*‐**Ru6**). Interestingly, this complex undergoes transformation into an isomer bearing two Cl ligands in the *cis*‐arrangement (*cis*‐**Ru6**). These two isomeric complexes exhibit similarly high thermodynamic stability, yet different application profiles in catalysis.

## Graphical abstract

New Grubbs‐type complexe containing macrocyclic N‐heterocyclic carbene ligand with two small aryl arms tied together was obtained and characterised. Interestingly, it can exist in two forms, *cis* and *trans*. Both of them exhibit similarly high thermodynamic stability, yet different application profiles in catalysis, however both can be used in difficult metathesis reactions, also leading to the formation of tetrasubstituted double bonds.

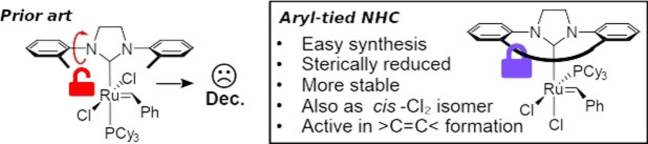

## Introduction

**Figure 1:**
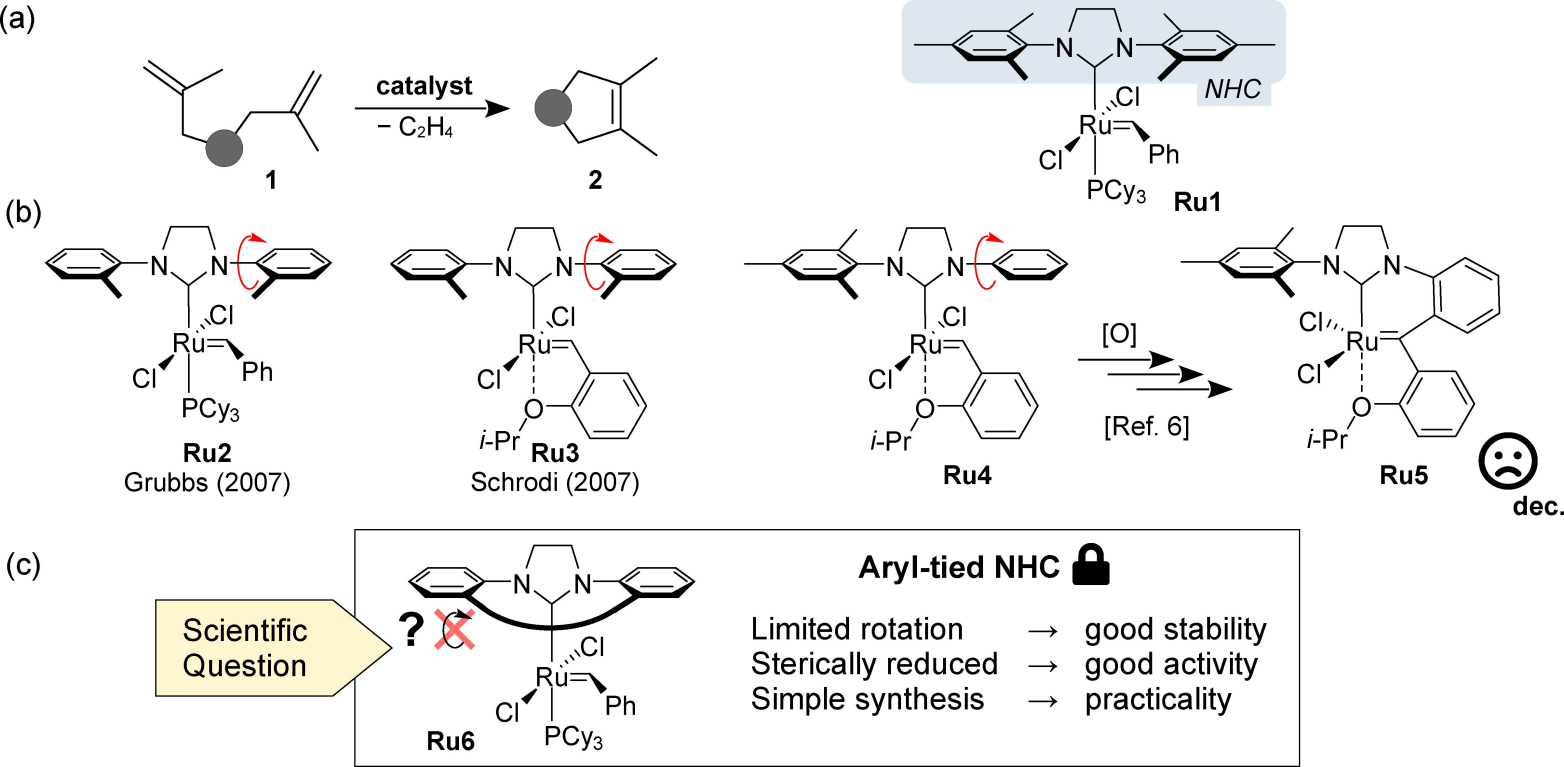
a) Challenging formation of a tetrasubstituted C−C double bond by olefin metathesis and a popular Grubbs’ NHC‐bearing catalyst **Ru1**. b) Selected complexes **Ru2–Ru4** featuring sterically reduced NHC ligands and one of multiple decomposition pathways of these catalysts.^[6]^ c) Proposed catalyst **Ru6** bearing an aryl‐tied NHC ligand.

Well‐defined ruthenium olefin metathesis catalysts are widely utilised in modern organic chemistry due to their universality and good stability toward air and moisture.^[1]^ Although the most iconic Grubbs’ 2^nd^ generation catalyst **Ru1** (Figure 1a) enabled the synthesis of myriads of products featuring variously substituted double bonds, the effective synthesis of tetrasubstituted or crowded alkenes still remains a challenge.^[2]^ To solve this limitation, Grubbs,^[3]^ Schrodi,^[3a]^ and others^[4]^ proposed that Ru complexes containing N‐heterocyclic carbene ligands (NHCs) with at least one *ortho* position in the *N*‐aryl arm unsubstituted (such as **Ru2**, Figure 1b) should provide the space required for the formation of the more sterically demanding metallacyclobutane en route to a tetrasubstituted olefin. This key observation led to the development of other catalysts bearing sterically reduced NHC ligands (such as **Ru3**).^[5]^

Unfortunately, in many cases improved activity in the formation of substituted C−C double bonds was at the expense of the catalyst's stability.^[5]^ It has been demonstrated that these catalysts typically decompose *via* the activation of C−C and C−H bonds in the aryl arms of the sterically reduced NHC ligands.^[7]^ Suresh *et al*. carried out a mechanistic study to explore the structural and energetic features leading to the decomposition pathways of such small‐NHC catalysts using density functional theory (DFT).^[8]^ The mechanistic study proved that the deactivation of these catalysts occur through C−H activation followed by C−H agostic interactions and σ‐bond metathesis. Removing the steric protection next to the Ru centre makes the catalyst not only less stable thermodynamically, but also more sensitive toward typical Ru catalysts poisons, such as Brønsted bases or oxygen. For example, Blechert *et al*. studied the decomposition of one of such small NHC catalysts and observed that in the presence of air, **Ru4** converts in a short time into catalytically inactive **Ru5** *via* a set of pericyclic cyclisation, oxidation, and rearomatisation reactions (Figure 1b).^[6]^

## Results and Discussion

**Scheme 1:**
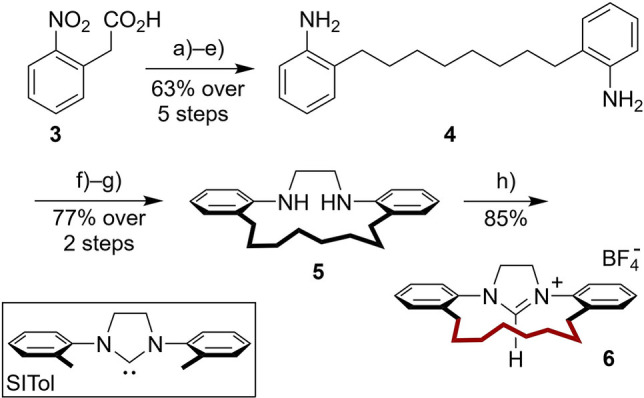
Structure of SITol ligand (in inset) and synthesis of macrocyclic NHC ligand precursor **6**. Conditions: a) MeOH, H_2_SO_4_ (cat.), 70 °C, 99 %; b) 1,6‐diiodohexane, K_2_CO_3_, DMSO, rt, 80 %; c) LiOH×H_2_O, THF, rt; d) K_2_CO_3_, DMF, 50 °C, 85 % (over two steps); e) Cu(OAc)_2_, NaBH_4_, EtOH, rt, 94 %; f) (CHO)_2_, MeCN/DCM (4 : 1 v/v), HCOOH, 90 %; g) NaBH_4_, MeOH:THF (1 : 1 v/v), 85 %; h) HC(OEt)_3_, NH_4_Cl, 90 °C, then NH_4_BF_4_, 55 %; or alternatively HC(OEt)_3_, NH_4_BF_4_, MW, 120 °C, 85 %. rt=room temperature; MW=microwave irradiation.

The results discussed above show that ruthenium catalysts bearing *N*‐aryl substituted NHC ligands lacking steric hindrance in the *ortho* position of the arene fragment, although generally more potent in the formation of tetrasubstituted C−C double bonds, can also give rise to intramolecular C−H insertion, leading to the formation of metathesis‐inactive ruthenium complexes. In our design, we assumed that covalently connecting (tying) two *N*‐aryl “arms” by a hydrocarbon chain of a proper length will limit the rotational freedom of the aryl fragments and possibly make the corresponding catalyst less prone to C−H activation and thus more stable (Figure 1c). The new NHC ligand was designed by a formal connection of two methyl groups in *o*‐tolyl substituents present in the known SITol NHC ligand of **Ru2** (Figure 1b) by an alkyl linker of a given length.^[9]^ With the help of Dreiding models^[10]^ (see Supporting Information for details) we predicted the minimal length of the tether. This simple “modelling” eventually led to the practical synthesis of the NHC ligand precursor **6** in which two phenyl substituents in the SITol‐type structure are linked by a C‐8 hydrocarbon chain (Scheme 1).

Commercially available acid **3** was converted into methyl ester, which was then alkylated with 1,6‐diiodohexane to produce **4** after hydrolysis, decarboxylation, and reduction of the nitro group (Scheme 1). Key macrocyclisation was conducted with glyoxal under optimised conditions, yielding the corresponding diimine in 90 % yield. This product was then conveniently reduced with sodium borohydride leading to a macrocyclic 1,2‐diamine **5** in good yield (77 % over 2 steps). Initially, the formation of imidazolinium salt **6** under classical conditions was lower yielding (50 %, see Supporting Information for details). Although such a yield can be considered quite satisfactory in the case of a macrocyclic architecture,^[11]^ we decided to further optimize this step. The use of microwave irradiation (MW) and a finely powdered NH_4_BF_4_ salt allowed us to obtain salt **6** in an improved yield of 85 % after 50 minutes of reaction (Scheme 1).

**Scheme 2:**
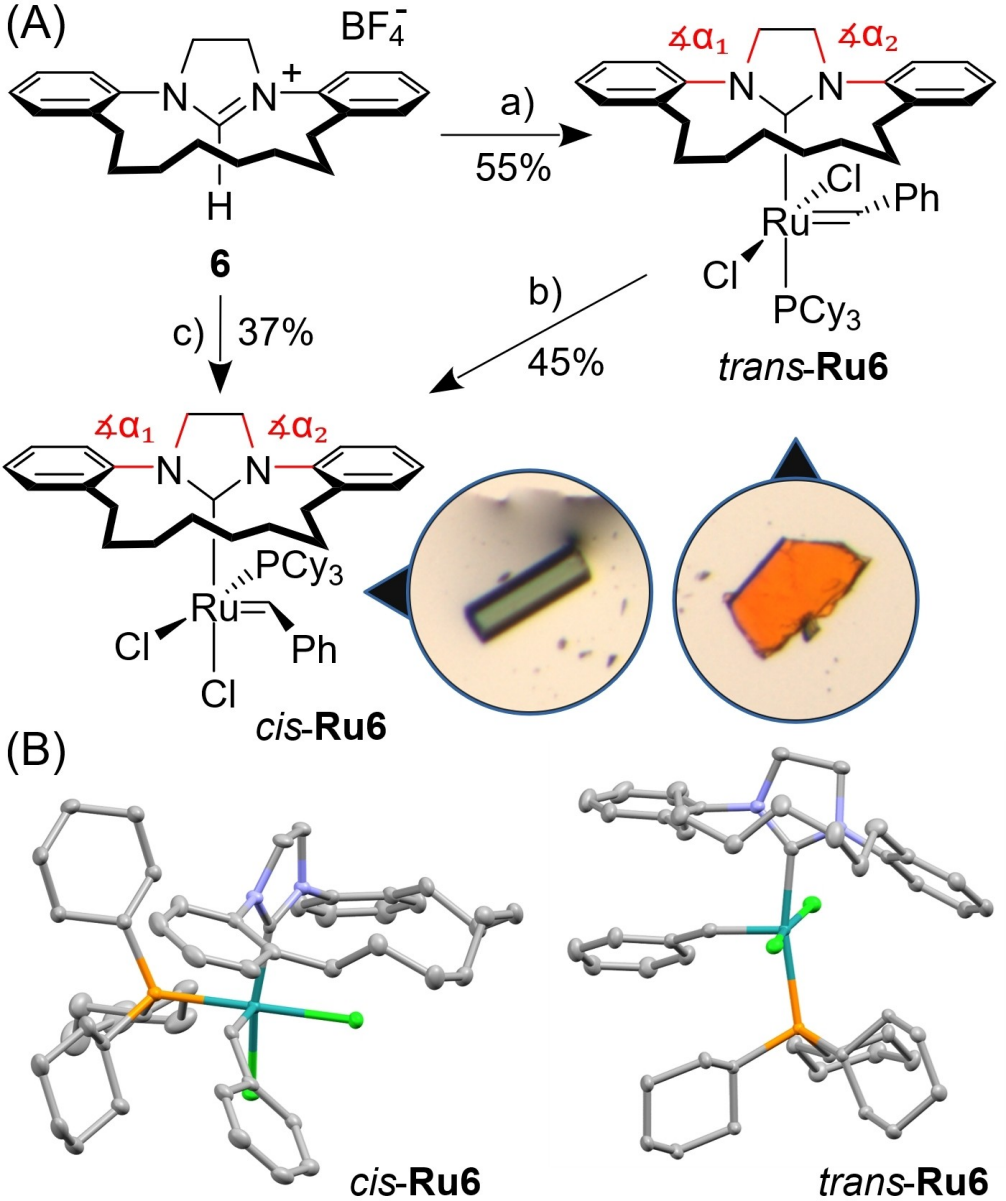
A) Synthesis of Ru complex *trans*‐**Ru6** and then its unexpected isomerisation to *cis*‐**Ru6**. Conditions: a) Potassium *tert*‐pentoxide, toluene, rt (0.5 h), then (PCy_3_)_2_(Cl)_2_Ru=CHPh (Grubbs’ 1^st^ gen. catalyst), 60 °C (0.5 h) then 80 °C (1 h), 55 %; b) SiO_2_, DCM/MeOH/H_2_O (94 : 5 : 1, v/v), rt, 45 %; c) *i*) Potassium *tert*‐pentoxide, toluene, rt (0.5 h), then (PCy_3_)_2_(Cl)_2_Ru=CHPh (Grubbs’ 1^st^ gen. catalyst), 60 °C (0.5 h) then 80 °C (1 h); *ii*) SiO_2_, DCM/MeOH (95 : 5, v/v), rt, 37 % (over two steps). B) Solid‐state structure of new complexes. Hydrogen atoms are omitted for clarity (for details see Supporting Information).

Having the macrocyclic NHC ligand precursor **6** in hand, we attempted to obtain the planned catalyst. Deprotonation of **6** with potassium *tert*‐pentoxide followed by the addition of the Grubbs 1^st^ generation complex ((PCy_3_)_2_(Cl)_2_Ru=CHPh) led to the formation of complex **Ru6**, as a pink‐brown solid, in 55 % isolated yield (Scheme 2A). The complex was stable in a solid form for several months (stored under inert atmosphere), as well as in DCM solution. However, when its solution in DCM/MeOH/H_2_O (originally purple) was deposited on silica gel and slowly eluted with the same solvent mixture, we noticed that a new green fraction appeared. The green band was then eluted from silica with DCM/MeOH/H_2_O, to yield, after recrystallisation from DCM, the new complex, obtained as a green solid in 45 % yield (Scheme 2, route b). The same green complex can be made directly from the ligand precursor **6** and Grubbs 1^st^ generation complex, in 37 % over two steps (Scheme 2, route c). We were fortunate to grow single crystals suitable for X‐ray measurement from both of these complexes, which, in addition to the analysis of ^1^H, ^13^C, ^31^P, and DEPT NMR spectra, allowed us to unambiguously assign the structures of both obtained products. (Scheme 2B). As a result, we found that in the presence of SiO_2_ and methanol, the initially formed *trans*‐**Ru6** isomerises to a complex where both Cl ligands are in the *cis‐*arrangement (*cis*‐**Ru6**). Interestingly, in the absence of SiO_2_, *trans*‐**Ru6** is stable in DCM solution.

To describe the differences in a solid state between the newly obtained complexes and the known *o*‐tolyl‐NHC Ru catalysts (those with reported crystallographic structures), we defined specific valence angles (α_1_ and α_2_; see Scheme 2 and Figure S5). The average of reported α_1_ and α_2_ angles in known SITol‐type ligands is 120(1)° and 121(1)° (see Supporting Information for details), whereas these angles are significantly smaller for *trans*‐**Ru6** (114.1(2)° and 118.5(2)°) and for *cis*‐**Ru6** (116.7(1)° and 119.9(2)°). This simple analysis shows that the introduction of the aliphatic chain connecting the *o*‐tolyl groups in the NHC ligand not only forces their *syn* conformation and blocks their free rotation, but also moves these arms up (which is witnessed by smaller angles α_1_ and α_2_).

The *trans*‐ to *cis*‐dichloro isomerisation process of **Ru6** is quite intriguing and requires further comment. In 2004, the first derivatives of the Hoveyda‐type catalyst (a complex featuring *a chelating* benzylidene ligand) with *cis*‐dichloro configuration have been reported.^[12]^ Since then, a large number of *cis*‐dichloro structures have appeared in the literature, most of them belonging to the Hoveyda‐type family of chelated complexes.^[13]^

On the other hand, it is quite difficult to obtain a related *cis*‐dichloro *Grubbs‐type catalyst* (a Ru complex featuring *a non‐chelating* benzylidene and two strongly σ‐donating and bulky ligands, such as PCy_3_ or NHC) using the same design principle.^[14]^ As a result, to the best of our knowledge, there are not many Grubbs 2^nd^ generation benzylidene catalysts in *cis*‐dichloro conformation known.[^13e^, ^15^] Therefore, we decided to study the isomerisation of the title Grubbs complex **Ru6** using DFT approach in four different solvent models (toluene, DCM, methanol, and water), focusing on the relative stabilities of the *trans* and *cis* isomers (for details see Supporting Information). Interestingly, the *trans* isomer was predicted to be slightly favoured over the *cis* isomer in toluene (by 2.7 kcal mol^−1^), but in methanol the *cis* isomer was predicted to be favoured over the *trans* isomer (by 2.2 kcal mol^−1^; for more details on the analysis of *cis–trans* isomerisation, see Supporting Information). Next, we considered that the concerted mechanism of isomerisation, where the *trans* isomer converts to the *cis* one through a single transition state, is operational in this case. Thus, the phosphine ligand (PCy_3_) changes its position in a manner similar to the Berry pseudorotation mechanism.^[16]^ This mechanism seems, according to our calculations, to be preferred over an alternative one‐step mechanisms in which the phosphine ligand is moved close to the *trans* position with respect to one chloride ion, while the second chloride shifts to the *trans* position with respect to the Ru=C bond (for details see Supporting Information). The estimated Gibbs free energy for the transition state of isomerisation (25.6–25.8 kcal mol^−1^ depending on the solvent) is similar to the energy barrier for *trans–cis* isomerisation of a Hoveyda‐like system obtained earlier,^[17]^ and are in good agreement with the observed experimental results, suggesting a very slow isomerisation.^[18]^

**Figure 2:**
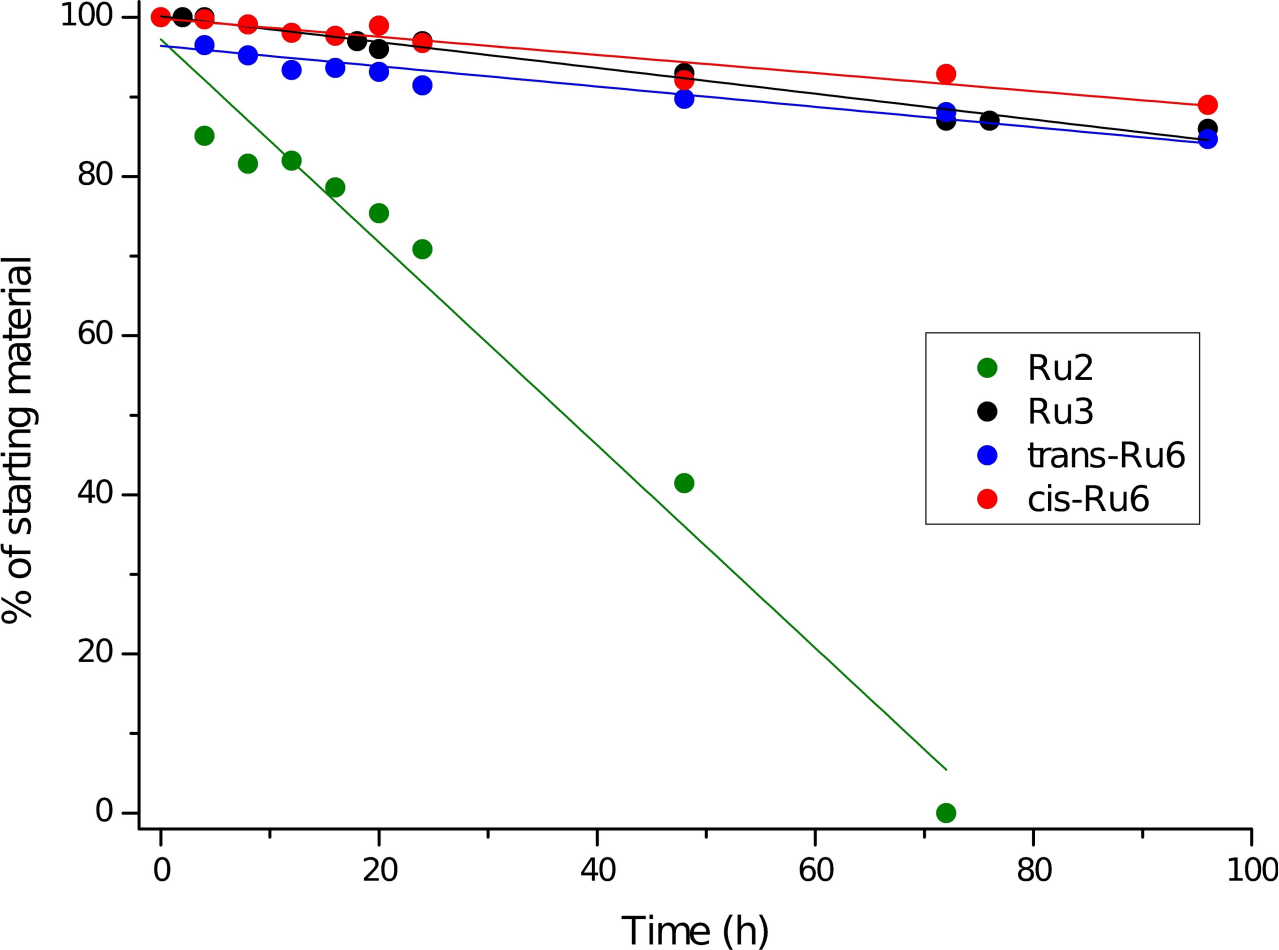
Catalysts stability in DCM‐*d*_2_ under argon at rt. Determined by ^1^H NMR, with 1,3,5‐trimetoxybenzene as an internal standard. Lines are visual aid only.

Returning to the bench chemistry, we were pleased to see that both complexes featuring the macrocyclic, small‐arms NHC ligand are very stable, not only in a solid form but also in a solution. As can be seen from Figure 2, the observed stability significantly extended the stability of the structurally related small‐NHC benzylidene complex **Ru2**. Surprisingly, the stability of the newly obtained complexes bearing “tied” aryl arms was as high as the one of complex **Ru3** (it is known that the *phosphine‐free* Hoveyda‐Grubbs‐type complexes exhibit in general higher thermal stability comparing to the corresponding Grubbs’ systems). The unique stability of phosphine‐containing **Ru6** complexes makes them potentially interesting in catalysis, especially under higher‐temperature conditions (see below).

**Scheme 3:**
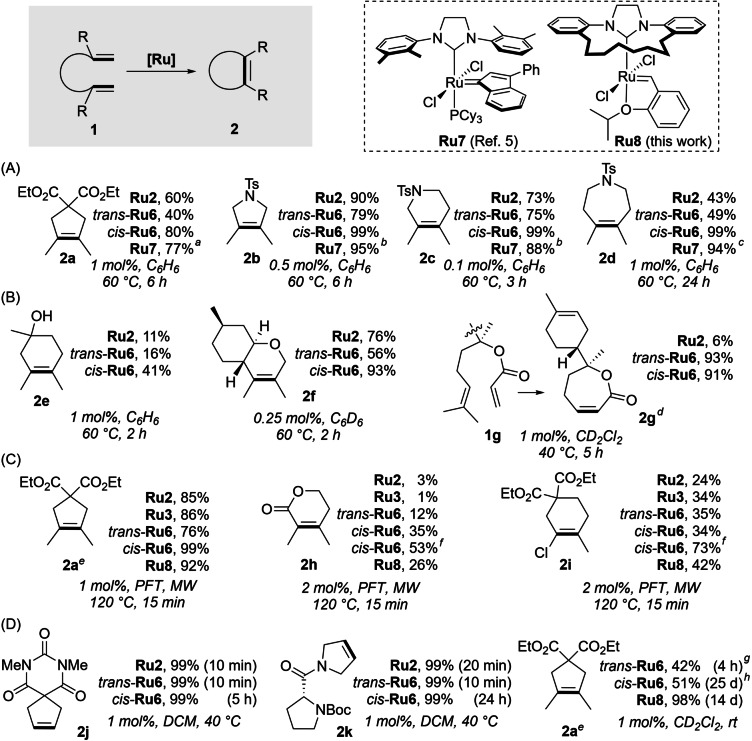
Ring‐closing metathesis scope and limitation study. The conversion was determined by GC (for substrates **1 a**–**e** and **1 j**–**k**) or by ^1^H NMR (for substrates **1 f**–**i**), for both techniques durene was used as an internal standard (for details see Supporting Information). [a] 0.5 mol %, 60 °C, PhMe, data taken from Ref. [5]. [b] 0.1 mol %, 60 °C, PhMe, data taken from Ref. [5]. [c] 4×0.25 mol %, 60 °C, PhMe, data taken from Ref. [5]. [d] Accompanied with the formation of higher cyclic and oligomeric products, for details, see Supporting Information. [e] Conversion determined by ^1^H NMR. [f] 2×2 mol % of catalyst used. [g] No further reaction progress observed after that. [h] Non‐decomposed *cis*‐**Ru6** still detected by NMR. PFT=perfluorotoluene.

Although similar in stability at room temperature, the two isomers of **Ru6** exhibited different catalytic properties. In the challenging ring‐closing metathesis (RCM) leading to carbo‐ and heterocycles **2 a**–**2 f** featuring tetrasubstituted C−C bonds, *cis*‐**Ru6** gave reproductively better results than its *trans*‐isomer (Scheme 3, rows A–B). In these challenging reactions *cis*‐**Ru6** outdistanced also its closest structural analogue—the phosphine‐containing catalyst **Ru2** ^[3a]^—offering 9 to 56 pp (percent points) higher conversions. Noteworthy, data reported in the literature^[5]^ allows to compare **Ru6** also with the state‐of‐the‐art complex bearing a medium‐size NHC, **Ru7**.[^5^, ^19^] Interestingly, while this trend is preserved for the derivative of isopulgeol (**2 f**), the RCM reaction of a diene **1 g**—bearing one terminal and one geminally disubstituted C−C double bond, based on a sesquiterpene alcohol, bisabolol—leads to almost quantitative conversion for both isomers of **Ru6**. Importantly, also in this case the productivity of *cis*‐**Ru6** was ahead of those exhibited in the same reaction by the SITol‐bearing **Ru2**, an industry‐standard tool designed particularly for such challenging transformations (Scheme 3, row B).^[3a]^

Subsequently, we focused on even more challenging substrates, precursors of products **2 h**–**2 i**. Requested by one of the Referees, we added to the pool of tested catalysts also the phosphine‐free relatives—the known SITol Hoveyda‐type complex (**Ru3**), and its macrocyclic‐NHC ligand‐based analogue **Ru8** (for its synthesis and crystal structure, see Supporting Information). To push the starters of this race to their limit of efficiency, we switched to more forcing conditions, consisting of using a microwave reactor and perfluorotoluene as a solvent.^[20]^ We decided also to include in this study the model malonate substrate **1 a** again,^[21]^ which gave with *cis*‐**Ru6** the quantitative conversion, surpassing the efficiency of *trans*‐**Ru6**, as well as **Ru2**, **Ru3**, and **Ru8** under the same conditions (Scheme 3, row C). Also for another problematic substrate (**1 h**), that contains an electron‐poor C−C double bond, we found that complex *cis*‐**Ru6** affords the highest conversions, exceeding the efficiency of other catalysts in a range from 9 to 34 pp (percent points). Noteworthy, in this case the SITol‐bearing **Ru2** (Grubbs‐type) and **Ru3** (Hoveyda‐type) were found to be completely ineffective. It should be noted that under the same conditions, the macrocyclic‐NHC catalysts **Ru6** and **Ru8** gave conversions up to 35 % and 26 %, respectively. Importantly, the conversion toward **2 h** could be further increased to 53 %, as demonstrated for *cis*‐**Ru6** by adding the catalyst portion‐wise (2×2 mol %, Scheme 3, row C). In turn, the formation of chloro‐substituted **2 i** revealed also the advantage of catalysts bearing the macrocyclic‐NHC ligand in comparison to their SITol analogues within the same catalyst type (Grubbs‐type: **Ru2** vs. **Ru6** and Hoveyda‐type: **Ru3** vs. **Ru8**). Similarly to the previous case, when *cis*‐**Ru6** was used in two portions (in total 2×2 mol %), the conversion toward chlorocyclohexene **2 i** become significantly increased (to 73 %, Scheme 3, row C).

It should be noted that the beneficial properties of *cis*‐**Ru6** are accompanied by its latent character, well‐illustrated during the formation of polyfunctional cycloalkenes **2 j** and **2 k** (Scheme 3 row D). In these two (rather straightforward) model RCM transformations^[22]^ *cis*‐**Ru6** required a visibly longer time to achieve complete conversion compared to **Ru2** and *trans*‐**Ru6** (Scheme 3, row D). The different performance of *cis* and *trans*‐**Ru6** complexes was also demonstrated in the RCM reaction of model challenging substrate **1 a** conducted at room temperature (Scheme 3, row D). We observed that *trans*‐**Ru6** activates faster than *cis*‐**Ru6** (42 % conversion of **1 a** after 4 h versus 3 %, respectively, for details see Figure S8 in Supporting Information), however, the latter is more stable in the reaction environment—being still present in the reaction mixture after 25 days. This leads to a much slower reaction at rt but better final yield compared with that obtained in the reaction catalysed by the *trans* isomer (Scheme 3, row D). Therefore, the superior properties of *cis*‐**Ru6** over *trans*‐**Ru6** in challenging metathesis transformations may originate from its slower activation, leading to higher stability in the reaction mixture. Interestingly, Hoveyda‐type **Ru8** led to very good results in tetrasubstituted C−C double bond formation not only at 120 °C but also at rt—needing of course much longer time (for details see Figure S9 in Supporting Information and compare results presented in Scheme 3, rows C and D).^[23]^

**Scheme 4:**
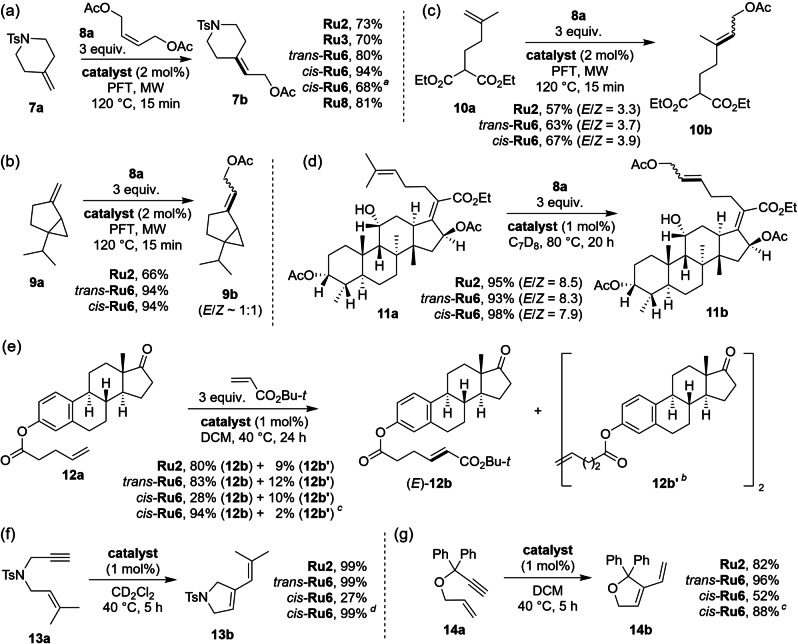
Cross‐metathesis and enyne cycloisomerisation examples. The yield was determined by ^1^H NMR (for products **7 b**, **9 b**–**11 b**), by GC (for product **14 b**), or as a yield of products isolated by column chromatography (for products **12 b** and **12 b′**). *E*/*Z* ratio determined by GC (products **9 b**, **10 b**) or by ^1^H NMR (product **11 b**). For all analytical measurements durene was used as internal standard. [a] 1 mol % of catalyst used. [b] Product of self‐metathesis of substrate **12 a**. [c] Alternative conditions: *cis*‐**Ru6** (1 mol %), PhMe, 80 °C. [d] Alternative conditions: *cis*‐**Ru6** (1 mol %), C_7_D_8_ (toluene‐*d*_8_), 80 °C.

Next, macrocyclic‐NHC complexes were tested in cross‐metathesis (CM) reactions (Scheme 4a–e). In a model reaction between challenging gem‐disubstituted alkene **7 a** ^[24]^ and (*Z*)‐2,3‐buten‐1,4‐diol diacetate (**8 a**) known complexes featuring SITol ligand, **Ru2** and **Ru3**, gave the expected product within 70 % yield. Notably, 10–24 pp higher yields (80 and 94 %) were obtained with *trans*‐**Ru6** and *cis*‐**Ru6**, respectively. The Hoveyda‐type analogue **Ru8** gave in the same reaction 81 % yield. Note that this CM was conducted under microwave irradiation, using previously established conditions (cf. Scheme 3). Both *trans*‐ and *cis*‐**Ru6** showed an advantage over commercial **Ru2** in CM of sabinene (**9 a**), a natural bicyclic monoterpene (Scheme 4b). Interestingly, in CM of an even more challenging geminal substrate **10 a**,^[25]^ *trans*‐ and *cis*‐**Ru6** as well as **Ru2** were found to be similarly productive, leading to yields of 57–67 % (Scheme 4c). In contrast, a derivative of a complex polyfunctional fusidic acid (**11 a**) reacted with **8 a** relatively easily, giving product **11 b** in a yield of 93–98 % with both *trans*‐ and *cis*‐**Ru6** and **Ru2** (Scheme 4d). In CM of **12 a** with an electron‐deficient partner,^[26]^ *tert*‐butyl acrylate, latent *cis*‐**Ru6** was initially found to be less productive, however, at higher temperature, led to a very good yield (94 %) of the expected estrone derivative (*E*)‐**12 b**, accompanied by only a tiny amount of the unwanted self‐CM product **12 b′** (“homodimer”).^[27]^ Finally, in a catalytic enyne cycloisomerisation, *trans*‐**Ru6** exhibited good reactivity, identical or slightly higher than the one displayed by benchmark SITol‐bearing **Ru2** (Scheme 4f, g). Interestingly, the more dormant *cis*‐**Ru6** required a higher temperature to operate (Scheme 4f, g, conditions *d*).

**Scheme 5:**
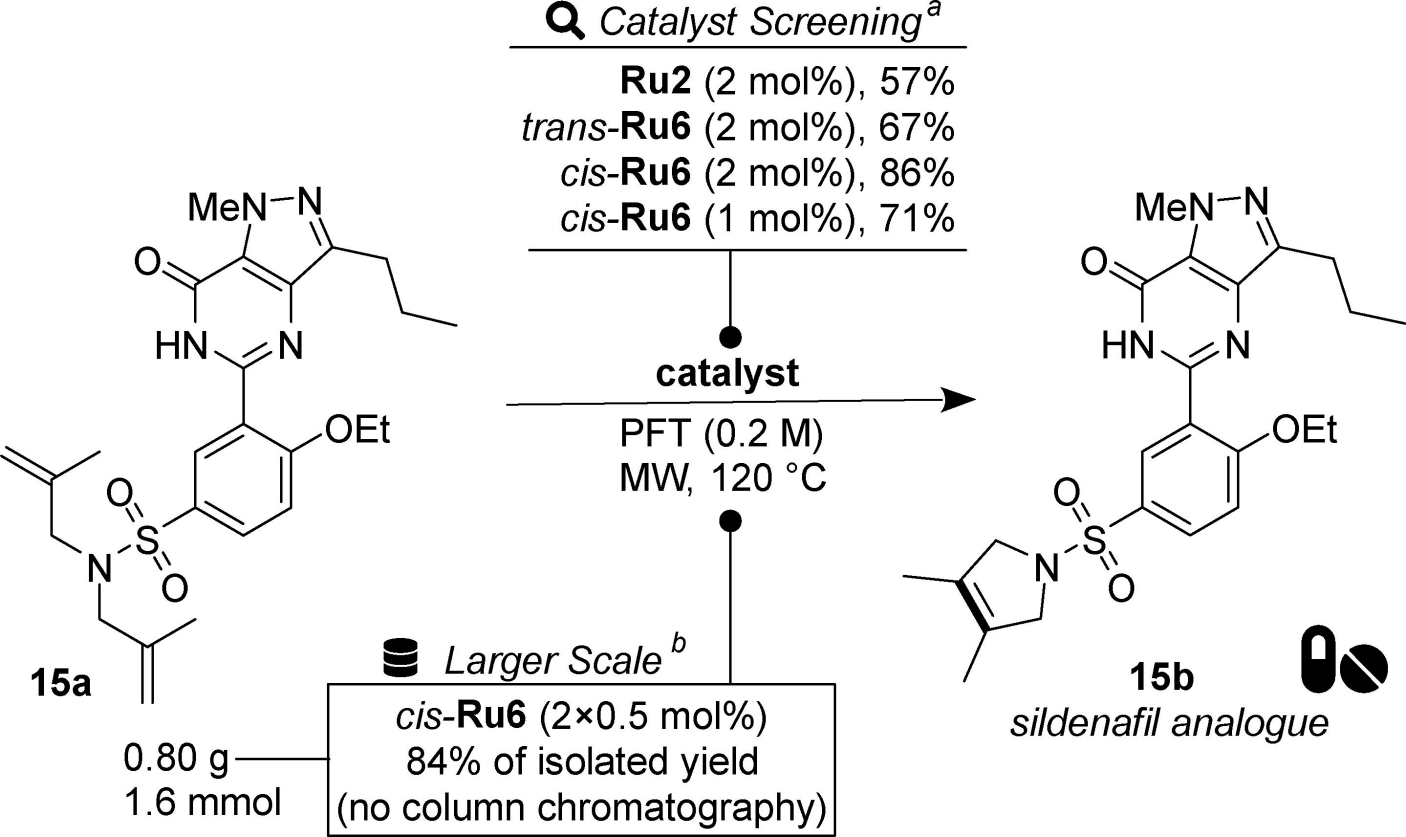
Ring‐closing metathesis of sildenafil analogue **15 a**. [a] Reaction carried out within 15 min, in 0.1 mmol scale, the conversion determined by ^1^H NMR using durene as an internal standard. [b] Reaction carried out within 2×15 min, in 1.6 mmol scale, with addition of catalyst in two portions, for details, see Supporting Information.

As the final test, we decided to perform the RCM reaction leading to sulphonamide **15 b**, a relative of sildenafil—a drug sold *inter alia* under the trade‐name Viagra™ (Scheme 5). Despite the similarity of substrate **15 a** to a sulphonamide **2 b** tested previously, we saw this substrate, due the presence of a number of Lewis basic groups in its structure, as a challenging target interesting in context of pharmaceutical chemistry. Applying a set of catalysts under previously established MW conditions (scale 0.1 mmol) leads to the following order of efficiency of the tested complexes: *cis*‐**Ru6**>*trans*‐**Ru6**>**Ru2**. Noteworthy, using *cis*‐**Ru6** in two 0.5 mol % portions allows to obtain analytically pure **15 b** at larger scale (1.6 mmol) in 84 % of isolated yield without column chromatography.

## Conclusion

An N‐heterocyclic carbene (NHC) ligand bearing two sterically reduced aryl arms connected by a C‐8 linker was obtained. Installation of this macrocyclic ligand on a ruthenium centre led to the formation of a ruthenium benzylidene complex *trans*‐**Ru6**. Interestingly, in the presence of methanol and silica, this complex undergoes isomerisation into *cis*‐**Ru6**, an isomer containing two Cl ligands in a *cis*‐arrangement, a geometry not very typical for classical Grubbs‐type phosphine‐containing benzylidene Ru complexes. Importantly, both of these complexes are significantly more stable than the structurally related small‐NHC catalyst **Ru2**, and generally exhibit a very good activity profile in olefin metathesis. In addition, *cis*‐**Ru6** that displays a dormant character and can be assumed to be *a latent metathesis catalyst*, is particularly active in the challenging formation of tetrasubstituted C−C double bonds. Because the formation of sterically hindered olefins was always the Achilles’ heel of ruthenium metathesis catalysts, the trait exhibited by *cis*‐**Ru6** seems to be of interest.

The results described herein show that, while it is well known that ruthenium catalysts bearing *N*‐aryl substituted NHC ligands lacking steric hindrance in the *ortho* position of the arene substituent gave rise to intramolecular C−H insertion, leading to the formation of metathesis‐inactive ruthenium complexes, connecting of these two aryl arms by a hydrocarbon linker can significantly stabilise the resulted catalyst. We believe that this observation is of importance for the further development of olefin metathesis catalysts.

**Supporting Information**: Detailed experimental procedures, copies on NMR spectra, computational methods, Cartesian coordinates of DFT‐optimized structures, and crystallographic details. Crystal structures have been deposited with the Cambridge Crystallographic Data Centre CCDC).^[28]^ The data can be obtained free of charge via www.ccdc.cam.ac.uk/structures.

## Conflict of interest

The authors declare no conflict of interest.

1

## Supporting information

As a service to our authors and readers, this journal provides supporting information supplied by the authors. Such materials are peer reviewed and may be re‐organized for online delivery, but are not copy‐edited or typeset. Technical support issues arising from supporting information (other than missing files) should be addressed to the authors.

Supporting Information

Supporting Information

Supporting Information

Supporting Information

Supporting Information

Supporting Information

Supporting Information

Supporting Information

Supporting Information

Supporting Information

## Data Availability

The data that support the findings of this study are available from the corresponding author upon reasonable request.
