# Supplementary material for: Ruthenium Olefin Metathesis Catalysts Bearing a Macrocyclic N‐Heterocyclic Carbene Ligand: Improved Stability and Activity
Source: Angew Chem Int Ed Engl. 2022 Apr 13;61(24):e202201472. doi: 10.1002/anie.202201472 (PMC9322543; doi:10.1002/anie.202201472)

checkCIF/PLATON report

You have not supplied any structure factors. As a result the full set of tests cannot be run.

No syntax errors found. CIF dictionary Interpreting this report

Datablock: I

Bond precision: C-C = 0.0041 A Wavelength=0.71073

Cell:	a=15.9530(13)	b=21.6658(17)	c=16.5514(14)
	alpha=90	beta=109.213(1)	gamma=90
Temperature:	100 K		

	Calculated	Reported
Volume	5402.1(8)	5402.1(8)
Space group	P 21/n	P2(1)/n
Hall group	-P 2yn	?
Moiety formula	C48 H67 Cl2 N2 P Ru, 3(C H2 Cl2)	C48 H67 Cl2 N2 P Ru, 3(C H2 Cl2)
Sum formula	C51 H73 Cl8 N2 P Ru	C51 H73 Cl8 N2 P Ru
Mr	1129.75	1129.75
Dx,g cm-3	1.389	1.389
Z	4	4
Mu (mm-1)	0.751	0.751
F000	2352.0	2352.0
F000’	2352.63	
h,k,lmax	19,26,20	19,26,20
Nref	10290	10282
Tmin,Tmax	0.914,0.928	0.834,0.929
Tmin’	0.829	

Correction method= MULTI-SCAN

Data completeness= 0.999 Theta(max)= 25.710

R(reflections)= 0.0326(8484) wR2(reflections)= 0.0850(10282)

S = 1.046 Npar= 606

The following ALERTS were generated. Each ALERT has the format
test-name_ALERT_alert-type_alert-level.
Click on the hyperlinks for more details of the test.

Alert level B

PLAT220_ALERT_2_B Large Non-Solvent C Ueq(max)/Ueq(min) ... 4.15 Ratio

---

Alert level C

```
PLAT214_ALERT_2_C Atom Cl4B    (Anion/Solvent) ADP max/min Ratio         4.30 prola
PLAT244_ALERT_4_C Low   'Solvent' Ueq as Compared to Neighbors of         C51
PLAT410_ALERT_2_C Short Intra H...H Contact  H25    ..  H45A    ..       1.96 Ang.
```

Alert level G

```
PLAT083_ALERT_2_G SHELXL Second Parameter in WGHT Unusually Large.       8.88
PLAT128_ALERT_4_G Alternate Setting of Space-group P21/c   .......      P21/n
PLAT232_ALERT_2_G Hirshfeld Test Diff (M-X)  Ru1    --  C25     ..       6.58 su
PLAT244_ALERT_4_G Low   'Solvent' Ueq as Compared to Neighbors of        C52B
PLAT244_ALERT_4_G Low   'Solvent' Ueq as Compared to Neighbors of         C52
PLAT302_ALERT_4_G Note: Anion/Solvent Disorder ..................          44 Perc.
PLAT779_ALERT_4_G Suspect or Irrelevant (Bond) Angle in CIF .... #          5
              CL4A -C51  -CL4B    1.555   1.555   1.555            22.20 Deg.
```

---

0 **ALERT level A** = Most likely a serious problem - resolve or explain
1 **ALERT level B** = A potentially serious problem, consider carefully
3 **ALERT level C** = Check. Ensure it is not caused by an omission or oversight
7 **ALERT level G** = General information/check it is not something unexpected

0 ALERT type 1 CIF construction/syntax error, inconsistent or missing data
5 ALERT type 2 Indicator that the structure model may be wrong or deficient
0 ALERT type 3 Indicator that the structure quality may be low
6 ALERT type 4 Improvement, methodology, query or suggestion
0 ALERT type 5 Informative message, check

It is advisable to attempt to resolve as many as possible of the alerts in all categories. Often the minor alerts point to easily fixed oversights, errors and omissions in your CIF or refinement strategy, so attention to these fine details can be worthwhile. In order to resolve some of the more serious problems it may be necessary to carry out additional measurements or structure refinements. However, the purpose of your study may justify the reported deviations and the more serious of these should normally be commented upon in the discussion or experimental section of a paper or in the "special_details" fields of the CIF. checkCIF was carefully designed to identify outliers and unusual parameters, but every test has its limitations and alerts that are not important in a particular case may appear. Conversely, the absence of alerts does not guarantee there are no aspects of the results needing attention. It is up to the individual to critically assess their own results and, if necessary, seek expert advice.

Publication of your CIF in IUCr journals

A basic structural check has been run on your CIF. These basic checks will be run on all CIFs submitted for publication in IUCr journals (*Acta Crystallographica*, *Journal of Applied Crystallography*, *Journal of Synchrotron Radiation*); however, if you intend to submit to *Acta Crystallographica Section C* or *E*, you should make sure that full publication checks are run on the final version of your CIF prior to submission.

Publication of your CIF in other journals

Please refer to the *Notes for Authors* of the relevant journal for any special instructions relating to CIF submission.

PLATON version of 24/03/2011; check.def file version of 16/03/2011

Datablock I - ellipsoid plot

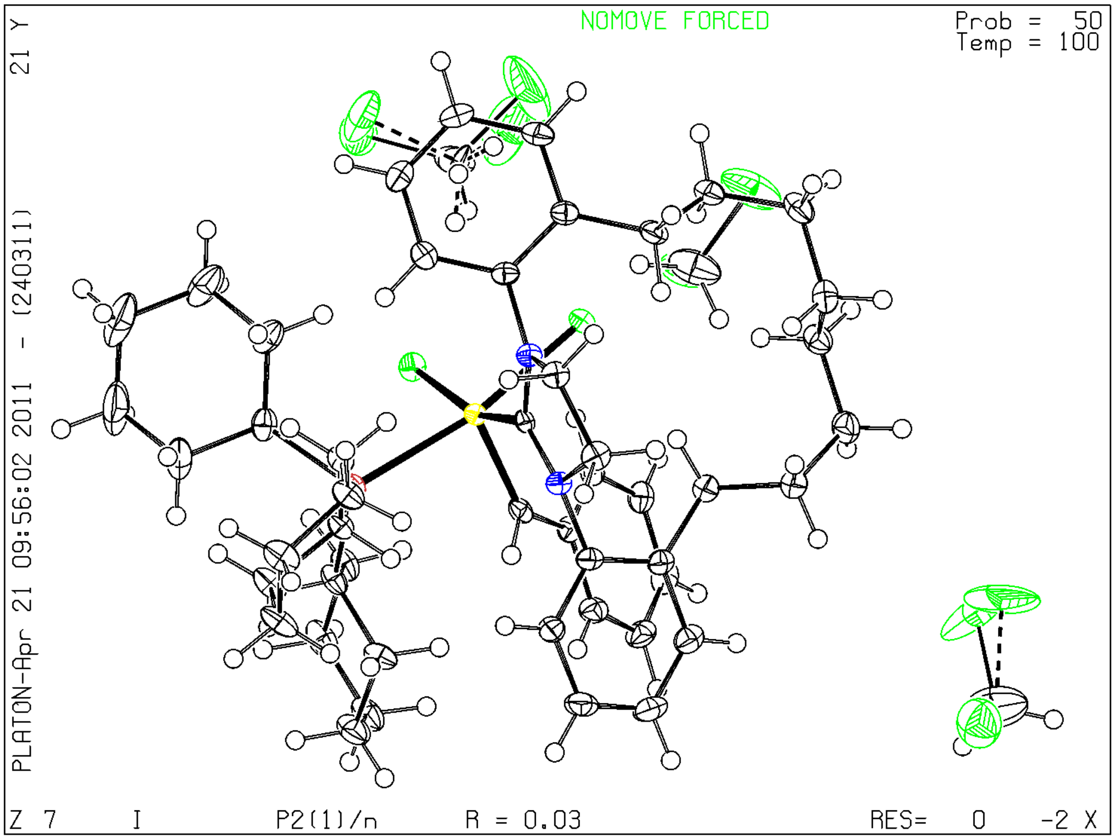

Supplement: Supplementary file 1 — Supporting Information [file ANIE-61-0-s003.pdf]
