# Supplementary material for: Ruthenium Olefin Metathesis Catalysts Bearing a Macrocyclic N‐Heterocyclic Carbene Ligand: Improved Stability and Activity
Source: Angew Chem Int Ed Engl. 2022 Apr 13;61(24):e202201472. doi: 10.1002/anie.202201472 (PMC9322543; doi:10.1002/anie.202201472)

checkCIF/PLATON report

No syntax errors found. **CIF dictionary** **Interpreting this report**

Datablock: ms_182

Bond precision: C-C = 0.0061 A Wavelength=0.71073

Cell:	a=10.6042(7)	b=11.2650(7)	c=15.0385(10)
	alpha=83.636(3)	beta=84.196(3)	gamma=64.031(3)
Temperature:	100 K		

	Calculated	Reported
Volume	1602.32(18)	1602.32(18)
Space group	P -1	P-1
Hall group	-P 1	-P1
Moiety formula	C33 H40 Cl2 N2 O Ru, C H4 O	C33 H40 Cl2 N2 O Ru, C H4 O
Sum formula	C34 H44 Cl2 N2 O2 Ru	C34 H44 Cl2 N2 O2 Ru
Mr	684.68	684.68
Dx,g cm-3	1.419	1.419
Z	2	2
Mu (mm-1)	0.689	0.689
F000	712.0	712.0
F000’	710.28	
h,k,lmax	13,13,18	12,13,18
Nref	6202	6137
Tmin,Tmax	0.921,0.937	0.653,0.745
Tmin’	0.917	

Correction method= MULTI-SCAN

Data completeness= 0.990 Theta(max)= 25.850

R(reflections)= 0.0421(4567) wR2(reflections)= 0.0935(6137)

S = 0.960 Npar= 377

The following ALERTS were generated. Each ALERT has the format
test-name_ALERT_alert-type_alert-level.
Click on the hyperlinks for more details of the test.

Alert level G

PLAT154_ALERT_1_G The su’s on the Cell Angles are Equal (x 10000) 300 Deg.

---

0 **ALERT level A** = In general: serious problem
0 **ALERT level B** = Potentially serious problem

```
0 ALERT level C = Check and explain
1 ALERT level G = General alerts; check

1 ALERT type 1 CIF construction/syntax error, inconsistent or missing data
0 ALERT type 2 Indicator that the structure model may be wrong or deficient
0 ALERT type 3 Indicator that the structure quality may be low
0 ALERT type 4 Improvement, methodology, query or suggestion
0 ALERT type 5 Informative message, check
```

Publication of your CIF in IUCr journals

A basic structural check has been run on your CIF. These basic checks will be run on all CIFs submitted for publication in IUCr journals (*Acta Crystallographica*, *Journal of Applied Crystallography*, *Journal of Synchrotron Radiation*); however, if you intend to submit to *Acta Crystallographica Section C* or *E*, you should make sure that full publication checks are run on the final version of your CIF prior to submission.

Publication of your CIF in other journals

Please refer to the *Notes for Authors* of the relevant journal for any special instructions relating to CIF submission.

PLATON version of 13/08/2009; check.def file version of 12/08/2009

Datablock ms_182 - ellipsoid plot

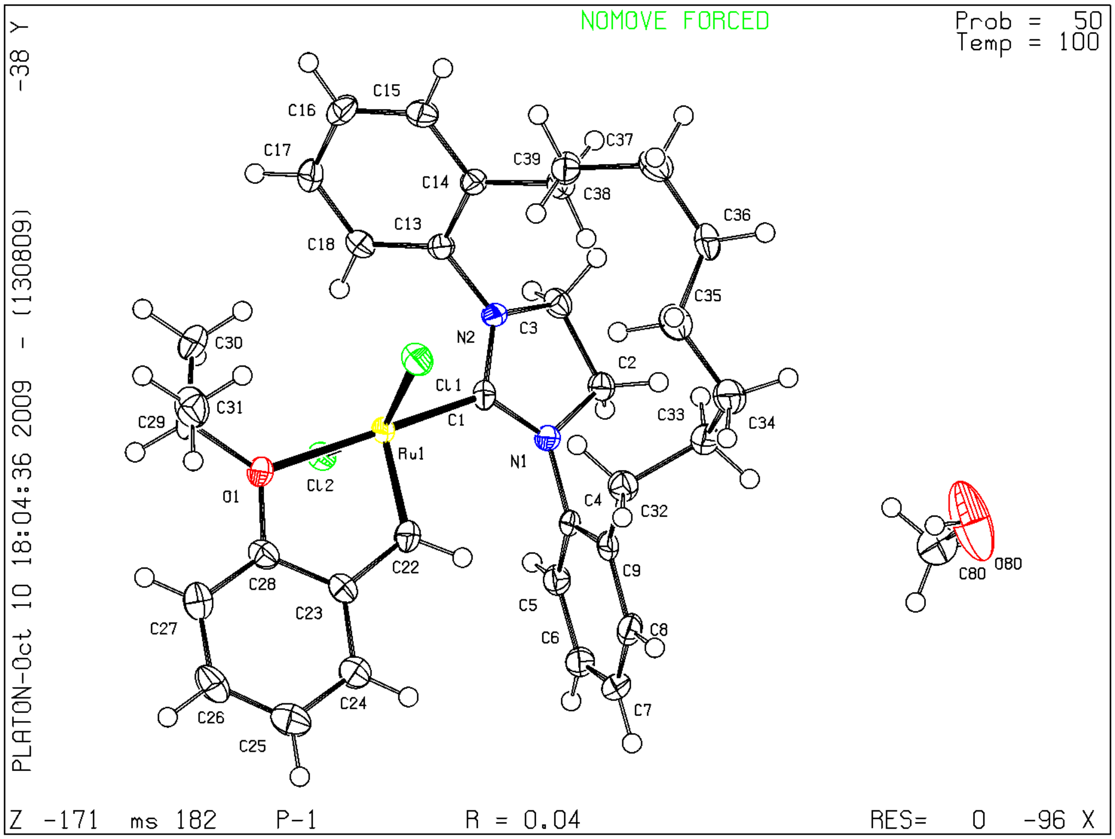

Supplement: Supplementary file 2 — Supporting Information [file ANIE-61-0-s006.pdf]
