# Supplementary material for: Ruthenium Olefin Metathesis Catalysts Bearing a Macrocyclic N‐Heterocyclic Carbene Ligand: Improved Stability and Activity
Source: Angew Chem Int Ed Engl. 2022 Apr 13;61(24):e202201472. doi: 10.1002/anie.202201472 (PMC9322543; doi:10.1002/anie.202201472)

checkCIF/PLATON report

You have not supplied any structure factors. As a result the full set of tests cannot be run.

No syntax errors found. CIF dictionary Interpreting this report

Datablock: shelxl

Bond precision: C-C = 0.0029 A Wavelength=0.71073

Cell:	a=9.6828(9)	b=13.0687(12)	c=19.1788(17)
	alpha=77.242(3)	beta=83.599(3)	gamma=76.298(2)
Temperature:	100 K		

	Calculated	Reported
Volume	2295.2(4)	2295.1(4)
Space group	P -1	P-1
Hall group	-P 1	?
Moiety formula	C48 H67 Cl2 N2 P Ru, C H2 Cl2	C48 H67 Cl2 N2 P Ru, C H2 Cl2
Sum formula	C49 H69 Cl4 N2 P Ru	C49 H69 Cl4 N2 P Ru
Mr	959.90	959.90
Dx,g cm-3	1.389	1.389
Z	2	2
Mu (mm-1)	0.645	0.646
F000	1008.0	1008.0
F000’	1007.11	
h,k,lmax	12,17,25	12,17,25
Nref	10953	10944
Tmin,Tmax	0.890,0.981	0.882,0.981
Tmin’	0.879	

Correction method= MULTI-SCAN

Data completeness= 0.999 Theta(max)= 27.880

R(reflections)= 0.0288(10007) wR2(reflections)= 0.0780(10944)

S = 1.059 Npar= 514

The following ALERTS were generated. Each ALERT has the format
test-name_ALERT_alert-type_alert-level.
Click on the hyperlinks for more details of the test.

Alert level G

```
PLAT005_ALERT_5_G No _iucr_refine_instructions_details in CIF ....          ?
PLAT232_ALERT_2_G Hirshfeld Test Diff (M-X)  Ru1    --  Cl1     ..       11.5 su
PLAT232_ALERT_2_G Hirshfeld Test Diff (M-X)  Ru1    --  Cl2     ..        8.0 su
```

```
PLAT232_ALERT_2_G Hirshfeld Test Diff (M-X)  Ru1    --  C1      ..        6.1 su
PLAT232_ALERT_2_G Hirshfeld Test Diff (M-X)  Ru1    --  C24     ..        9.8 su
PLAT431_ALERT_2_G Short Inter HL..A Contact  Cl3    ..  Cl3     ..       3.21 Ang.
```

```
   0 ALERT level A = Most likely a serious problem - resolve or explain
   0 ALERT level B = A potentially serious problem, consider carefully
   0 ALERT level C = Check. Ensure it is not caused by an omission or oversight
   6 ALERT level G = General information/check it is not something unexpected

   0 ALERT type 1 CIF construction/syntax error, inconsistent or missing data
   5 ALERT type 2 Indicator that the structure model may be wrong or deficient
   0 ALERT type 3 Indicator that the structure quality may be low
   0 ALERT type 4 Improvement, methodology, query or suggestion
   1 ALERT type 5 Informative message, check
```

It is advisable to attempt to resolve as many as possible of the alerts in all categories. Often the minor alerts point to easily fixed oversights, errors and omissions in your CIF or refinement strategy, so attention to these fine details can be worthwhile. In order to resolve some of the more serious problems it may be necessary to carry out additional measurements or structure refinements. However, the purpose of your study may justify the reported deviations and the more serious of these should normally be commented upon in the discussion or experimental section of a paper or in the "special_details" fields of the CIF. checkCIF was carefully designed to identify outliers and unusual parameters, but every test has its limitations and alerts that are not important in a particular case may appear. Conversely, the absence of alerts does not guarantee there are no aspects of the results needing attention. It is up to the individual to critically assess their own results and, if necessary, seek expert advice.

Publication of your CIF in IUCr journals

A basic structural check has been run on your CIF. These basic checks will be run on all CIFs submitted for publication in IUCr journals (*Acta Crystallographica*, *Journal of Applied Crystallography*, *Journal of Synchrotron Radiation*); however, if you intend to submit to *Acta Crystallographica Section C* or *E*, you should make sure that full publication checks are run on the final version of your CIF prior to submission.

Publication of your CIF in other journals

Please refer to the *Notes for Authors* of the relevant journal for any special instructions relating to CIF submission.

PLATON version of 18/07/2011; check.def file version of 04/07/2011

Datablock shelxl - ellipsoid plot

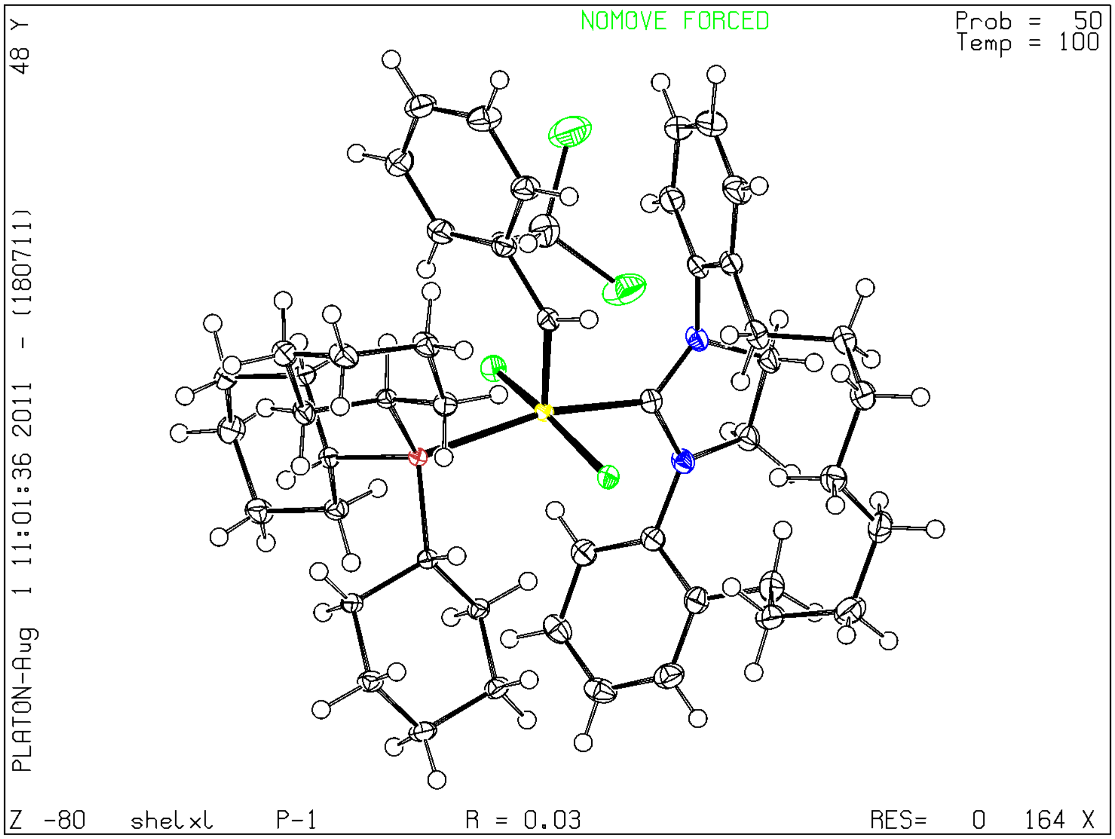

Supplement: Supplementary file 3 — Supporting Information [file ANIE-61-0-s005.pdf]
