# Supplementary material for: Ruthenium Olefin Metathesis Catalysts Bearing a Macrocyclic N‐Heterocyclic Carbene Ligand: Improved Stability and Activity
Source: Angew Chem Int Ed Engl. 2022 Apr 13;61(24):e202201472. doi: 10.1002/anie.202201472 (PMC9322543; doi:10.1002/anie.202201472)
Supplement: Supplementary file 5 — Supporting Information [file ANIE-61-0-s004.pdf]

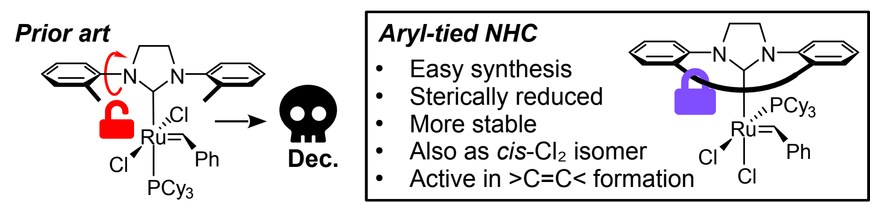
Prior art
N
N
Cl
Ru
Cl
Ph
PCy3
Dec.
Aryl-tied NHC
Easy synthesis
Sterically reduced
More stable
Also as cis-Cl2 isomer
Active in >C=C< formation
N
N
PCy3
Ru
Cl
Ph
Cl
