# Supplementary material for: Ruthenium Olefin Metathesis Catalysts Bearing a Macrocyclic N‐Heterocyclic Carbene Ligand: Improved Stability and Activity
Source: Angew Chem Int Ed Engl. 2022 Apr 13;61(24):e202201472. doi: 10.1002/anie.202201472 (PMC9322543; doi:10.1002/anie.202201472)
Supplement: Supplementary file 7 — Supporting Information [file ANIE-61-0-s008.pdf]

Angewandte Chemie
Eine Zeitschrift der Gesellschaft Deutscher Chemiker

Supporting Information

Ruthenium Olefin Metathesis Catalysts Bearing a Macrocyclic N-Heterocyclic Carbene Ligand: Improved Stability and Activity

W. Kośnik, D. Lichosyt, M. Śnieżek, A. Janaszkiewicz, K. Woźniak, M. Malińska, B. Trzaskowski, A. Kajetanowicz, K. Grela**

Table of Contents

S1. General remarks 3

S2. Preparation of ligand precursor, catalysts and substrates for metathesis reactions 3

S2.1. Synthesis of 1,8-bis(2-nitrophenyl)octane 3

S2.2. Synthesis of 2,2'-(octane-1,8-diyl)dianiline (4) 5

S2.3. Synthesis of diimine derivative 5

S2.4. Synthesis of diamine derivative (5) 6

S2.5. Synthesis of (*E*)-2^4,2^5-dihydro-2^1*H*-2(1,3)-imidazol-3-iuma-1,3(1,2)-dibenzenacyclo-undecaphan-2^3-ium chloride (6-Cl) 6

S2.6. Synthesis of (*E*)-2^4,2^5-dihydro-2^1*H*-2(1,3)-imidazol-3-iuma-1,3(1,2)-dibenzenacyclo-undecaphan-2^3-ium tetrafluoroborate (6-BF_4) 7

S2.7. Synthesis of complex *trans*-Ru6 8

S2.8. Synthesis of complex *cis*-Ru6 9

S2.9. Synthesis of complex Ru8 10

S2.10. Synthesis of compound 1g 10

S2.11. Synthesis of compound 15a 11

S3. RCM and Ene-Yne reactions 11

S3.1. General procedures 11

S3.2. Preparation of product 2g 12

S3.3. Preparation of product 15b 13

S4. CM reactions 14

S4.1. General procedures 14

S4.2. Results for model CM reaction of allylbenzene (S1) with 8a 15

S4.3. Preparation of product 9b 15

S5. DRRM reactions 16

S5.1. General procedure for DRRM 16

S3.1. Results for model DRRM reaction of *tert*-butyl((1-(cyclopent-3-en-1-yl)but-3-en-1-yl)oxy)dimethylsilane (S3) 16

S6. X-ray diffraction 17

S7. CSD survey 26

S8. Dreiding Models of NHC ligands bearing chains of different length 29

S9. Computational studies 30

S9.1 Computational details 30

S9.2 Computational results 31

S10. Reaction profiles for RCM of 1a 34

S11. Copy of NMR and MS spectra 37

S1. General remarks

The catalyst preparation was carried out under argon in pre-dried glassware using Schlenk techniques. The anhydrous solvents were dried by distillation over the following drying agents and were transferred under argon: THF (K/benzophenone), toluene (Na), *n*-pentane, *n*-hexane, CH_2Cl_2 (CaH_2). Flash column chromatography was performed using ALDRICH silica gel 60 (230-400 mesh). Analytical thin-layer chromatography (TLC) was performed using silica gel 60 F_{254} precoated plates (0.25 mm thickness) with a fluorescent indicator. NMR spectra were recorded on Varian Unity Plus 200 MHz and Agilent 400 MHz in $CDCl_3$, CH_2Cl_2; chemical shifts (δ) are given in ppm relative to TMS, coupling constants are (*J*) in Hz. IR spectra were recorded on JASCO FT/IR-6200: wavenumbers are in cm^{-1}. MS (ESI) spectra were recorded by Quattro LC (triple quadrupole mass spectrometer). GC measurements were done on PE Clarus 580 with InertCap 5MS-Sil column and GC/MS on PE Clarus 680/SQ8C with InertCap 5MS-Sil column. Model substrates (**1a-f**, **1h-k**, **7a**, **10a**, **11a-14a**) and products (**2a-f**, **2h-k**, **7b**, **10b**, **11b-14b**) were previously obtained and characterised.[1,2,3,4,5] All other commercially available chemicals were used as received.

S2. Preparation of ligand precursor, catalysts and substrates for metathesis reactions

S2.1. Synthesis of 1,8-bis(2-nitrophenyl)octane

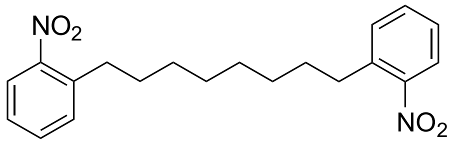

To a solution of **2-nitrophenylacetic acid** (14 g, 75 mmol) in methanol (600 mL), H_2SO_4 (4.2 mL, 95%) was added. The reaction mixture was stirred at 70 °C for 5 hours. The solvent was evaporated and the residue was dissolved in CH_2Cl_2, washed with saturated $NaHCO_3$ solution, water, and brine, dried over $MgSO_4$, and concentrated under vacuum. The crude product was purified by column chromatography (SiO_2, 50% EtOAc : *c*-hex) to afford **methyl 2-(2-nitrophenyl)acetate** (14 g, 96%) as a light yellow solid.

---

[1] M. Bieniek, R. Bujok, M. Cabaj, N. Lugan, G. Lavigne, D. Arlt, K. Grela, *J. Am. Chem. Soc.*, **2006**, *128*, 13652-13653.
[2] A. Michrowska, K. Mennecke, U. Kunz, A. Kirschning , K. Grela, *J. Am. Chem. Soc.*, **2006**, *128*, 13261-13267.
[3] K. Grela, A. Michrowska, M. Bieniek, M. Kim , R. Klajn, *Tetrahedron*, **2003**, *59*, 4525-4531.
[4] S. Zhang, Z. Shi, W. Cao, T. Gao, H. Deng, *J. Chem. Res.*, **2009**, 381-383.
[5] S. Kotha, A. Ch. Deb, R. V. Kumar, *Bioorg. Med. Chem. Lett.*, **2005**, *15*,1039-1043.

^{1}H NMR (200 MHz, $CDCl_3$): δ = 8.08 (dd, *J* = 8.1, 1.4 Hz, 1H, ArH), 7.65 – 7.39 (m, 2H, ArH), 7.34 (dd, *J* = 7.4, 1.6 Hz, 1H, ArH), 4.00 (s, 2H, CH_2), 3.68 (s, 3H, OCH_3) ppm; ^{13}C NMR (50 MHz, $CDCl_3$): δ = 170.6, 133.8, 133.5, 129.9, 128.8, 125.4, 52.4, 39.7 ppm; DEPT (50 MHz, $CDCl_3$): δ = 133.8, 133.5, 128.8, 125.4, 52.4, 39.7 ppm.

To a solution of **methyl 2-(2-nitrophenyl)acetate** (5.47 g, 28 mmol) in DMSO (94 mL), K_2CO_3 (11.6 g, 84 mmol) was added. When the reaction mixture had achieved a dark blue color, 1,6-diiodohexane (2.3 mL, 14 mmol) was added dropwise. The reaction mixture was stirred at room temperature for 3 days. Next, it was concentrated and then diluted with MTBE, washed with water and brine, dried over $MgSO_4$, and concentrated under vacuum. The residue was purified by column chromatography (SiO_2, 20% then 50% EtOAc : *c*-hex) to afford **dimethyl 2,9-bis(2-nitrophenyl)decanedioate** (5.3 g, 80%) as a light yellow solid.

^{1}H NMR (200 MHz, $CDCl_3$): δ = 7.84 (dd, *J* = 8.1, 1.2 Hz, 2H, ArH), 7.68 – 7.28 (m, 6H, ArH), 4.13 (t, *J* = 7.4 Hz, 2H, CH), 3.64 (s, 6H, OCH_3), 2.27 – 1.94 (m, 2H, CH_2), 1.94 – 1.65 (m, 2H, CH_2), 1.26 (br s, 8H, CH_2) ppm; ^{13}C NMR (50 MHz, $CDCl_3$) δ 173.2, 149.5, 133.5, 132.9, 129.7, 128.0, 124.6, 52.2, 45.9, 32.9, 28.9, 27.4 ppm; DEPT (50 MHz, $CDCl_3$): δ = 132.9, 129.7, 127.9, 124.5, 52.2, 45.9, 32.9, 28.9, 27.4 ppm; IR (film): $\tilde{\nu}$ = 2949, 2933, 2859, 1738, 1527, 1353, 1209, 1169, 853, 786, 740 cm^{-1}; MS (ESI): 495.0 $[M+Na]^+$.

To solution of **dimethyl 2,9-bis(2-nitrophenyl)decanedioate** (2.96 g, 6.27 mmol) in THF (55 mL) and water (14 mL) $LiOH{\cdot}H_2O$ (0.79 g, 18.81 mmol) was added. The reaction mixture was stirred at room temperature for 20 hours. The solvents were evaporated and the residue was dissolved in CH_2Cl_2, washed with 10% HCl, water, and brine, dried over $NaSO_4$, and concentrated under vacuum. The crude product **2,9-bis(2-nitrophenyl)decanedioic acid** was dissolved in DMF (55 mL) and K_2CO_3 (1.73 g, 12.54 mmol) was added. The reaction mixture was stirred at 50 °C for 2 hours. The solvents were evaporated, the residue was dissolved in MTBE, and washed with water and brine, dried over $MgSO_4$, and concentrated under vacuum. The residue was purified by column chromatography (SiO_2, 10% Et_2O:*c*-hex) to afford **1,8-bis(2-nitrophenyl)octane** (1.89 g, 85%) as a light yellow solid.

^{1}H NMR (400 MHz, $CDCl_3$): δ = 7.85 (d, *J* = 8.0 Hz, 2H, ArH), 7.50 (td, *J* = 7.7, 1.2 Hz, 2H, ArH), 7.37 – 7.28 (m, 4H, ArH), 2.90 – 2.81 (m, 4H, CH_2), 1.70 – 1.55 (m, 4H, CH_2), 1.45 – 1.25 (m, 8H, CH_2) ppm; ^{13}C NMR (101 MHz, $CDCl_3$): δ 149.4, 137.6, 132.7, 131.8, 126.8, 124.5, 32.9, 30.7, 29.5, 29.2 ppm; DEPT (101 MHz, $CDCl_3$): δ 132.7, 131.8, 126.8, 124.5, 32.9, 30.7, 29.5, 29.2 ppm; IR (film from CH_2Cl_2): $\tilde{\nu}$ = 2929, 2856, 1609, 1526, 1463, 1348, 859, 786, 742, 703, 664 cm^{-1}; HRMS

(ESI): m/z: calcd for $C_{20}H_{24}N_2O_4Na$: 379.1634 $[M+Na]^+$, found: 379.1621; elemental analysis calcd (%) for $C_{20}H_{24}N_2O_4$: C 67.40, H 6.79, N 7.86; found: C 67.38, H 6.71, N 7.80.

S2.2. Synthesis of 2,2'-(octane-1,8-diyl)dianiline (4)

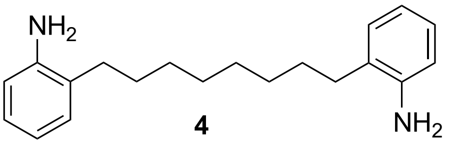

4

The solution of **1,8-bis(2-nitrophenyl)octane** (8.41 g, 23.6 mmol) and $Cu(OAc)_2$ (1.29 g, 7.08 mmol) in EtOH (236 mL), was cooled to 0 °C, and $NaBH_4$ (5.36 g, 142 mmol) was added portionwise. The reaction mixture was warmed to room temperature and stirred overnight. After this time, water was added and the reaction mixture was filtered by Celite®545. The solvents were evaporated and the residue was dissolved in CH_2Cl_2, washed with water and brine, dried over $NaSO_4$, and concentrated under vacuum. The residue was purified by column chromatography (SiO_2, 50% EtOAc:*c*-hex) to afford **4** (6.58 g, 94%) as a light yellow solid.

^1H NMR (400 MHz, $CDCl_3$): δ = 7.16 – 6.98 (m, 4H, ArH), 6.77 (td, J = 7.4, 1.1 Hz, 2H, ArH), 6.70 (d, J = 7.5 Hz, 2H, ArH), 3.63 (s, 4H, NH_2), 2.60 – 2.42 (m, 4H, CH_2), 1.79 – 1.53 (m, 4H, CH_2), 1.51 – 1.30 (m, 8H, CH_2) ppm; ^{13}C NMR (101 MHz, $CDCl_3$): δ = 144.0, 129.4, 126.8, 126.8, 118.7, 115.5, 31.2, 29.6, 29.5, 28.7 ppm; DEPT (101 MHz, $CDCl_3$): δ = 129.4, 126.8, 118.7, 115.5, 31.2, 29.6, 29.5, 28.7 ppm; IR (film from $CHCl_3$): $\tilde{\nu}$ = 3463, 3374, 3021, 2926, 2854, 1618, 1583, 1497, 1457, 1311, 1270, 749 cm^{-1}; HRMS (ESI): m/z: calcd for $C_{20}H_{29}N_2$: 297.2331 $[M+H]^+$, found: 297.2321; elemental analysis calcd (%) for $C_{20}H_{28}N_2$: C 81.03, H 9.52, N 9.45; found: C 81.15, H 9.51, N 9.57.

S2.3. Synthesis of diimine derivative

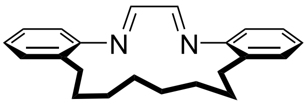

To a solution of diamine **4** (1.58 g, 5.32 mmol) in CH_3CN (20 mL), glyoxal (0.61 mL, 5.32 mmol, 40 wt. % in H_2O) was added dropwise. Next, 0.03 mL of HCOOH was added and a yellow solid was precipitated. To the reaction mixture, 5 mL of CH_2Cl_2 was added. The reaction was stirred very intensive at room temperature for 1 hour and left overnight in the fridge. The crude solid product was filtered and washed with CH_3CN and pentane to afford **diimine derivative** (1.518 g, 90%) as a yellow solid.

^1H NMR (400 MHz, $CDCl_3$): δ = 8.20 (s, 2H, CH=N), 7.34 – 7.15 (m, 6H, ArH), 6.94 – 6.80 (m, 2H, ArH), 2.85 – 2.71 (m, 4H, CH_2), 1.78 – 1.45 (m, 4H, CH_2), 1.38 – 1.09 (m, 4H, CH_2), 1.09 –

0.82 (m, 4H, CH_2) ppm; ^{1}H NMR (400 MHz, THF-*d*$_8$): δ = 8.30 – 8.04 (m, 2H, CH=N), 7.32 – 7.07 (m, 6H, ArH), 6.98 – 6.81 (m, 2H, ArH), 2.91 – 2.66 (m, 4H, CH_2), 1.67 – 1.52 (m, 4H, CH_2), 1.28 – 1.10 (m, 4H, CH_2), 1.07 – 0.84 (m, 4H, CH_2) ppm; ^{13}C NMR (101 MHz, $CDCl_3$): δ = 160.9, 150.9, 136.2, 130.2, 127.0, 126.9, 117.2, 31.9, 29.0, 28.1, 26.7 ppm; DEPT (101 MHz, $CDCl_3$): δ = 160.90, 130.2, 127.0, 126.9, 117.2, 31.9, 29.0, 28.0, 26.7 ppm; IR (KBr): $\tilde{\nu}$ = 3064, 3049, 3018, 2958, 2922, 2848, 1611, 1482, 1444, 776, 736 cm^{-1}; HRMS (ESI): *m*/*z*: calcd for $C_{22}H_{26}N_2Na$: 341.1994 $[M+Na]^+$, found: 341.1996; elemental analysis calcd (%) for $C_{22}H_{26}N_2 \cdot 1/5\ H_2O$: C 82.05, H 8.26, N 8.70; found: C 82.04, H 8.18, N 8.71.

S2.4. Synthesis of diamine derivative (5)

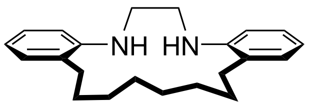

5

The solution of macrocyclic diamine derivative (2.08 g, 6.53 mmol) in Et_2O (30 mL) and MeOH (30 mL) was cooled to 0 °C and $NaBH_4$ (1.48 g, 39.2 mmol) was added portionwise. After this time, the reaction was warmed to room temperature and stirred overnight. The solvents were evaporated and the residue was dissolved in CH_2Cl_2, washed with water and brine, dried over $NaSO_4$, and concentrated under vacuum. The residue was purified by column chromatography (SiO_2, 20% EtOAc:*c*-hex) to afford **5** (1.79 g, 85%) as a light yellow solid.

^{1}H NMR (400 MHz, $CDCl_3$): δ = 7.17 (td, *J* = 7.8, 1.5 Hz, 2H, ArH), 7.10 (dd, *J* = 7.3, 1.4 Hz, 2H, ArH), 6.84 – 6.68 (m, 4H, ArH), 3.99 (br s, 2H, NH), 3.50 (br s, 4H, CH_2), 2.59 – 2.40 (m, 4H, CH_2), 1.82 – 1.59 (m, 4H, CH_2), 1.54 – 1.28 (m, 8H, CH_2) ppm; ^{13}C NMR (101 MHz, $CDCl_3$): δ = 145.6, 129.4, 127.4, 127.0, 117.8, 110.7, 43.4, 30.1, 28.7, 27.2, 26.9 ppm; DEPT (101 MHz, $CDCl_3$): δ = 129.4, 127.0, 117.8, 110.7, 43.4, 30.1, 28.7, 27.2, 26.9 ppm; IR (KBr): $\tilde{\nu}$ = 3422, 3060, 3033, 3009, 2915, 2850, 1600, 1580, 1504, 1466, 1454, 1444, 1425, 1304, 1261, 746 cm^{-1}; HRMS (ESI): *m*/*z*: calcd for $C_{22}H_{31}N_2$: 323.2487 $[M+H]^+$, found: 323.2473; elemental analysis calcd (%) for $C_{22}H_{30}N_2$: C 81.94, H 9.38, N 8.69; found: C 82.07, H 9.36, N 8.55.

S2.5. Synthesis of (*E*)-2^4,2^5-dihydro-2^1*H*-2(1,3)-imidazol-3-iuma-1,3(1,2)-dibenzenacyclo-undecaphan-2^3-ium chloride (6-Cl)

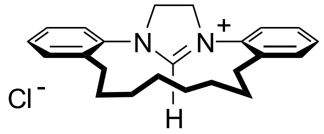

To a solution of diamine **5** (1.12 g, 3.47 mmol) in triethyl orthoformate (15 mL), NH_4Cl (0.19 g, 3.47 mmol) was added. The reaction mixture was stirred at 90 °C for 48 hours. After this time the reaction was cooled to room temperature and the product crystallized. After filtration, the

precipitate was dissolved in CH_2Cl_2, washed with water, dried over $MgSO_4$, and concentrated under vacuum. The crude product was re-crystallized from CH_2Cl_2/Et_2O to afford **6-Cl** (0.7 g, 55%) as a light beige solid.

^1H NMR (400 MHz, $CDCl_3$): δ = 8.33 (s, 1H, CHN), 8.27 – 8.14 (m, 2H, ArH), 7.41 – 7.28 (m, 2H, ArH), 7.28 – 7.17 (m, 4H, ArH), 4.67 (s, 4H, CH_2N), 2.70 – 2.51 (m, 4H, CH_2), 1.80 – 1.54 (m, 4H, CH_2), 1.54 – 1.40 (m, 4H, CH_2), 1.40 – 1.21 (m, 4H, CH_2) ppm; ^1H NMR (400 MHz, THF-d_8): δ = 9.52 (s, 1H, CHN), 8.56 (d, *J* = 7.8 Hz, 2H, ArH), 7.31 – 7.21 (m, 4H, ArH), 7.21 – 7.10 (m, 2H, ArH), 4.63 (s, 4H, CH_2N), 2.79 – 2.68 (m, 4H, CH_2), 1.69 – 1.59 (m, 4H, CH_2), 1.54 – 1.24 (m, 8H, CH_2) ppm; ^{13}C NMR (101 MHz, $CDCl_3$): δ = 157.4, 138.2, 133.9, 130.3, 130.2, 128.5, 128.1, 54.1, 29.9, 29.4, 26.9, 25.7 ppm; DEPT (101 MHz, $CDCl_3$): δ = 157.4, 130.3, 130.2, 128.5, 128.1, 54.1, 29.9, 29.4, 26.9, 25.7 ppm; IR (KBr): $\tilde{\nu}$ = 3627, 3302, 3220, 2925, 2854, 1622, 1269, 776, 763 cm^{-1}; HRMS (ESI): *m/z*: calcd for $C_{23}H_{29}N_2$: 333.2331 $[M-Cl]^+$, found: 333.2335; elemental analysis calcd (%) for $C_{23}H_{29}ClN_2 \cdot H_2O$: C 71.39, H 8.07, N 7.24, Cl 9.16; found: C 71.47, H 8.09, N 7.28, Cl 9.00.

S2.6. Synthesis of (*E*)-2^4,2^5-dihydro-2^1*H*-2(1,3)-imidazol-3-iuma-1,3(1,2)-dibenzenacyclo-undecaphan-2^3-ium tetrafluoroborate (6-BF_4)

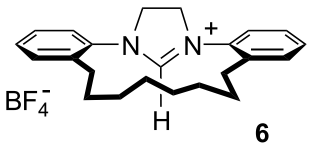

6

To a solution of diamine **5** (0.484 g, 1.5 mmol) in triethyl orthoformate (10 mL), well-powdered NH_4BF_4 (0.157 g, 1.5 mmol) was added under argon. The reaction mixture was stirred at 120 °C for 50 minutes in the MW reactor. After this time, the reaction was cooled to ~0 °C and the product crystallized. After filtration, the precipitate was dissolved in CH_2Cl_2 and washed with water, dried over $MgSO_4$, and concentrated under vacuum. The crude product was re-crystallized from CH_2Cl_2/*n*-pentane to afford **6-BF_4** (0.538 g, 85%) as an off-white solid.

^1H NMR (400 MHz, $CDCl_3$): δ = 7.82 (s, 1H, CHN), 7.77 (dd, *J* = 7.8, 1.2 Hz, 2H, ArH), 7.44 – 7.20 (m, 6H, ArH), 4.56 (s, 4H, CH_2N), 2.78 – 2.46 (m, 4H, CH_2), 1.79 – 1.56 (m, 4H, CH_2), 1.55 – 1.41 (m, 4H, CH_2), 1.41 – 1.27 (m, 4H, CH_2) ppm; ^{13}C NMR (101 MHz, $CDCl_3$): δ = 156.8, 138.3, 133.56, 130.6, 130.4, 128.3, 127.8, 53.7, 29.7, 29.3, 26.9, 25.6 ppm; IR (KBr): $\tilde{\nu}$ = 3062, 2930, 2861, 1631, 1267, 1092, 1055, 771 cm^{-1}; HRMS (ESI): *m/z*: calcd for $C_{23}H_{29}N_2$: 333.2331 $[M-BF_4]^+$, found: 333.2338; elemental analysis calcd (%) for $C_{23}H_{29}BF_4N_2$: C 65.73, H 6.95, N 6.67, F 18.08; found: C 65.49, H 6.87, N 6.58, F 17.84.

S2.7. Synthesis of complex *trans*-Ru6

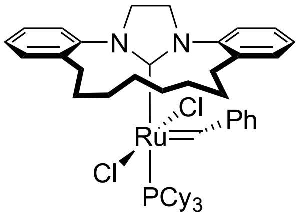

trans-**Ru6**

Potassium *tert*-pentoxide (1.7 M in toluene, 0.84 mL, 1.43mmol) was added at room temperature to a suspension of **6-BF$_4$** (0.6 g, 1.43 mmol) in dry toluene (60 mL) under argon. Reaction mixture was stirred at room temperature for 30 minutes, then I generation Grubbs catalyst (0.98 g, 1.19 mmol) was added, the stirring was continued at 60 C for 30 minutes, and next at 80 °C for 1 hour. After cooling down, the reaction mixture was filtered through a silica gel (10% EtOAc : *n*-hexane) under argon. Solvents were evaporated and the product was dissolved in *n*-hexane and concentrated (three times). The complex was redissolved in hexane and allowed to stand for 30 minutes to precipitate. The catalyst was dried under vacuum to afford complex ***trans*-Ru6** (0.57 g, 55%) as a pink–brown solid.

^{1}H NMR (400 MHz, CD_2Cl_2): δ = 19.10 (s, 1H, Ru=CH), 8.13 (dd, *J* = 5.6, 3.6 Hz, 1H, ArH), 7.53 – 7.28 (m, 4H, ArH), 7.22 – 6.98 (m, 4H, ArH), 6.98 – 6.22 (m, 4H, ArH), 4.27 – 3.91 (m, 4H), 3.37 (td, *J* = 13.6, 3.6 Hz, 1H), 3.10 (br s, 1H), 2.76 – 2.40 (m, 1H), 2.14 – 1.82 (m, 5H), 1.78 – 1.34 (m, 24H), 1.34 – 1.19 (m, 3H), 1.15 – 0.93 (m, 10H), 0.93 – 0.73 (m, 4H) ppm; ^{13}C NMR (101 MHz, CD_2Cl_2): δ = 296.9, 220.6, 151.8, 144.4, 142.8, 140.9, 139.4, 132.5, 131.5, 131.1, 130.2, 129.4, 129.1, 128.6, 128.1, 127.1, 56.7, 56.6, 56.1, 56.0, 33.1, 32.9, 32.6, 32.4 (*n*-Hex), 31.9, 31.1, 31.0, 30.0, 29.6, 29.5, 29.4, 29.1, 28.6, 28.5, 28.5, 28.4, 28.3, 28.1, 26.9, 23.5 (*n*-Hex), 14.7 (*n*-Hex) ppm; DEPT (101 MHz, CD_2Cl_2): δ = 296.9, 132.5, 131.5, 131.1, 130.2, 129.4, 129.1, 128.6, 128.1, 127.1, 56.7, 56.6, 56.1, 56.0, 33.1, 32.9, 32.6, 32.4 (*n*-Hex), 31.9, 31.1, 31.0, 30.0, 29.6, 29.5, 29.4, 29.1, 28.6, 28.5, 28.5, 28.4, 28.3, 28.1, 26.9, 23.5 (*n*-Hex), 14.7 (*n*-Hex) ppm; ^{31}P NMR (162 MHz, CD_2Cl_2): δ = 26.61 ppm; IR (KBr): $\tilde{\nu}$ = 3053, 2922, 2848, 1490, 1445, 1424, 1279, 759, 737 cm^{-1}; HRMS (ESI): *m*/*z*: calcd for $C_{48}H_{67}N_2PClRu$: 839.3774 [M-Cl]$^+$, found: 839.3813; elemental analysis calcd (%) for $C_{48}H_{67}N_2PCl_2Ru$ · 3/5 *n*-C_6H_{14}: C 66.88, H 8.20, N 3.02, Cl 7.65; found: C 66.97, H 8.39, N 3.01, Cl 7.51. CCDC 2087688.

S2.8. Synthesis of complex *cis*-Ru6

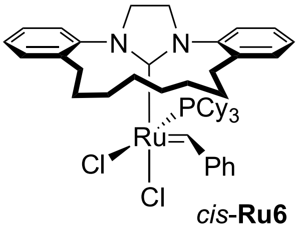

cis-**Ru6**

Procedure A: All following operations were performed in the argon-filled glove-box. The complex ***trans*-Ru6** (0.10 g, 0.114 mmol) was dissolved in a minimal amount (~1 mL) of mixture of DCM/MeOH/H_2O (94:5:1) and slowly passed through a silica gel column (24 g) using mixture of DCM/MeOH/H_2O (94:5:1) as an eluent. The slow elution of the initially purple complex resulted in the formation of a green fraction, which was collected and evaporated. The residue was suspended in a minimal (~1 mL) amount of DCM and filtered off, washed with a minimal amount (~1 mL) of cold DCM and dried under vacuum to afford ***cis*-Ru6** (0.045 g, 45%) as a green solid.

Procedure B: All following operations were performed in the argon-filled glove-box. Potassium *tert*-pentoxide (1.7 M in toluene, 0.235 mL, 0.40 mmol) was added at room temperature to a suspension of **6-BF4** (0.168 g, 0.40 mmol) in dry toluene (15 mL). The reaction mixture was stirred at room temperature for 30 minutes, then I generation Grubbs catalyst (0.277 g, 0.337 mmol) was added, the stirring was continued at 60 °C for 30 minutes, and next at 80 °C for 1 hour. After cooling down, the precipitate was filtered off and the filtrate was adsorbed on the top of the silica gel column (48 g of SiO_2, preconditioned with solution of 5% MeOH in CH_2Cl_2 and slowly eluted with a solution of 5% MeOH in CH_2Cl_2. The green fraction was collected and evaporated. The residue was suspended in a minimal amount of (~2 mL) DCM and filtered off, washed with a minimal amount (~1 mL) of cold DCM and dried under vacuum to afford ***cis*-Ru6** (0.108 g, 37%) as a green solid.

^{1}H NMR (400 MHz, CD_2Cl_2): δ = 16.66 (d, *J* = 19.9 Hz, 1H, Ru=CH), 8.26 (dd, *J* = 7.5, 1.2 Hz, 1H, ArH), 7.79 (dd, *J* = 5.7, 3.6 Hz, 2H, ArH), 7.63 – 7.14 (m, 9H, ArH), 6.98 (dd, *J* = 5.6, 3.8 Hz, 1H, ArH), 4.23 – 3.61 (m, 4H), 2.94 – 2.67 (m, 1H), 2.43 – 2.30 (m, 3H), 2.29 – 2.16 (m, 1H), 2.14 – 2.03 (m, 2H), 1.98 – 1.77 (m, 12H), 1.74 – 1.68 (m, 3H), 1.61 – 1.53 (m, 3H), 1.50 – 1.38 (m, 5H), 1.36 – 1.24 (m, 13H), 1.21 – 1.10 (m, 3H), 1.07 – 0.96 (m, 2H), 0.87 – 0.80 (m, 1H) ppm; ^{13}C NMR (101 MHz, CD_2Cl_2): δ = 217.1, 148.8, 143.7, 141.6, 141.4, 140.8, 133.6, 131.4, 130.2, 129.6, 129.2, 129.1, 127.6, 127.1, 127.1, 124.7, 57.6, 54.3, 54.1, 53.8, 52.9, 37.2, 37.0, 30.3, 30.1, 30.1, 29.0, 28.8, 28.7, 28.4, 28.3, 28.2, 26.9, 25.2, 24.3 ppm; DEPT (101 MHz, CD_2Cl_2): δ = 133.6, 131.4, 130.2, 129.6, 129.2, 129.1, 127.6, 127.2, 127.1, 124.7, 57.6, 54.3, 54.1, 53.8, 52., 50.9 (CH_3OH), 37.2, 37.0, 30.3, 30.1, 30.1, 27.0, 28.8, 28.7, 28.4, 28.3, 28.2, 26.9, 25.2, 24.3 ppm; ^{31}P NMR (162 MHz, CD_2Cl_2): δ = 32.72 ppm; IR (KBr): $\tilde{\nu}$ = 3430, 3060, 2925, 2848, 1624, 1488, 1447, 1397, 1270, 1246, 1207, 1174, 753 cm^{-1}; HRMS (ESI): *m*/*z*: calcd for $C_{48}H_{67}N_2PClRu$: 839.3774 [M-Cl]$^+$, found: 839.3785;

elemental analysis calcd (%) for $C_{48}H_{67}N_2PCl_2Ru \cdot 9/4\ CH_3OH \cdot 1/4\ CH_2Cl_2$: C 62.64, H 7.96, N 2.89, Cl 9.15; found: C 62.43, H 7.73, N 2.96, Cl 8.97. CCDC 2087687

S2.9. Synthesis of complex Ru8

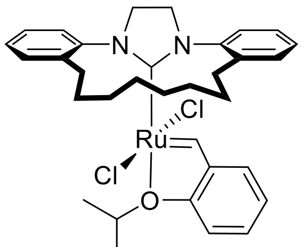

Ru8

LiHMDS (221 mg, 1.32 mmol) was added at room temperature to a suspension of **6-BF4** (0.555 g, 1.32 mmol) in dry THF (5 mL) under argon. Then I generation Hoveyda-Grubbs catalyst (360 mg, 0.60 mmol) was added and reaction mixture was stirred at room for 5 minutes. Next, CuCl (66 mg, 0.66 mmol) was added and stirring was continued for 1h. After completion, the solvent was evaporated and the residue was purified by column chromatography (SiO_2, 20% EtOAc:*n*-hexane) to afford **Ru8** (178 mg, 45%) as a green solid.

^{1}H NMR (400 MHz, CD_2Cl_2): δ =16.35 (s, 1H, Ru=CH), 8.65 (s, 1H), 7.76 – 7.14 (m, 8H), 7.01 – 6.77 (m, 3H), 4.98 (hept, *J* = 6.1 Hz, 1H), 4.40 (s, 2H), 4.28 – 4.01 (m, 2H), 3.11 – 2.44 (m, 4H), 2.00 – 1.17 (m, 18H) ppm; ^{13}C NMR (101 MHz, CD_2Cl_2): δ = 293.9, 210.5, 153.0, 144.3, 143.2, 142.6, 141.8, 141.0, 131.5, 130.4, 130.1, 130.1, 129.6, 129.2, 128.9, 128.2, 128.0, 122.9, 122.4, 113.5, 75.3, 55.8, 53.0, 32.2, 30.9, 30.7, 29.7, 28.3, 27.7, 27.6, 27.2, 22.5, 22.2 ppm.

S2.10. Synthesis of compound 1g

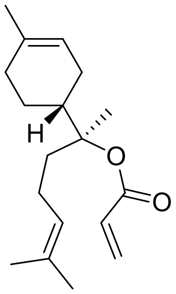

1g

α-(–)-Bisabolol (2.6 mL, 93%, 10 mmol) and Et_3N (2.78 mL, 20 mmol) were dissolved in 50 mL of dry CH_2Cl_2 and the solution was cooled to 0 °C under an argon atmosphere; then a solution of acryloyl chloride (1.27 mL, 15 mmol, 97%) in 10 mL of dry DCM was added dropwise. The resulting mixture was stirred overnight. Then additional portion of acryolyl chloride (1.27 mL, 15 mmol, 97%) was added and stirred for 2 h. Next reaction mixture was poured into 30 mL of cold water, and extracted with CH_2Cl_2 (3 x 30 mL). The combined organic phases were dried over Na_2SO_4. The solvent was removed under reduced pressure, and the residue was purified on a silica gel column eluted with hexane/EtOAc (gradient, 100:0 to 95:5, v/v) to afford the desired acrylate **1g** as an colorless liquid (1.15 g, 42%).

^{1}H NMR (400 MHz, $CDCl_3$): δ = 6.29 (dd, *J* = 17.3, 1.6 Hz, 1H), 6.05 (dd, *J* = 17.3, 10.3 Hz, 1H), 5.73 (dd, *J* = 10.3, 1.6 Hz, 1H), 5.42 – 5.30 (m, 1H), 5.15 – 5.01 (m, 1H), 2.34 – 2.18 (m, 1H), 2.12 – 1.75 (m, 9H), 1.71 – 1.61 (m, 6H), 1.58 (s, 3H), 1.44 (s, 3H), 1.42 – 1.24 (m, 1H) ppm; ^{13}C NMR

(101 MHz, $CDCl_3$): δ = 165.5, 134.3, 131.8, 130.4, 129.4, 124.2, 120.3, 87.5, 40.6, 35.7, 31.0, 26.5, 25.8, 23.8, 23.5, 22.1, 20.7, 17.7ppm; HRMS (ESI): *m*/*z*: calcd for $C_{18}H_{28}O_2Na$: 299.1987 $[M+Na]^+$, found: 299.1977.

S2.11. Synthesis of compound 15a

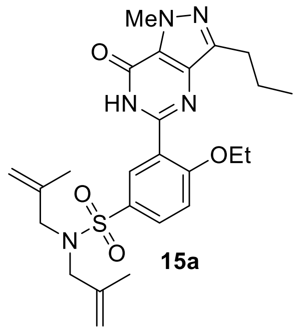

15a

2-Methyl-*N*-(2-methyl-2-propenyl)-2-propen-1-amine (250 mg, 2 mmol) was added to a solution of 4-ethoxy-3-(6,7-dihydro-1-methyl-7-oxo-3-propyl-1*H*-pyrazolo[4,3-d]pyrimidin-5-yl)benzene-1-sulfonyl chloride[6] (847 mg, 2 mmol) and triethylamine (0.556 mL, 4 mmol) in methylene chloride (15 mL) at 0 °C. Then, reaction mixture was stirred overnight at 40°C. After completion, reaction mixture was concentrated and product was purified using column chromatography (SiO_2, hexane/EtOAc gradient, 100:0 to 50:50, v/v) to afford the product **15a** as an colorless solid (536 mg, 54%).

^1H NMR (400 MHz, $CDCl_3$): δ = 10.87 (s, 1H), 8.87 (d, *J* = 2.4 Hz, 1H), 7.88 (dd, *J* = 8.8, 2.4 Hz, 1H), 7.10 (d, *J* = 8.8 Hz, 1H), 4.85 (d, *J* = 22.9 Hz, 4H), 4.35 (q, *J* = 7.0 Hz, 2H), 4.25 (s, 3H), 3.75 (s, 4H), 2.91 (t, *J* = 7.6 Hz, 2H), 1.90 – 1.77 (m, 2H), 1.69 – 1.56 (m, 9H), 1.00 (t, *J* = 7.4 Hz, 3H) ppm; ^{13}C NMR (101 MHz, $CDCl_3$): δ = 159.0, 153.8, 147.1, 146.7, 140.0, 138.5, 133.8, 131.4, 130.8, 124.6, 120.9, 114.9, 112.9, 66.1, 53.4, 38.3, 27.9, 22.5, 20.2, 14.7, 14.2 ppm; HRMS (ESI): *m*/*z*: calcd for $C_{25}H_{34}N_5O_4S$: 500.2326 $[M+H]^+$, found: 500.2328.

S3. RCM and Ene-Yne reactions

S3.1. General procedures

<u>Procedure C:</u>

Comparative experiments with model substrates (**1a-g**, **1j-k, 13a** and **14a**) were performed in a dry solvent under argon at 40, 60 or 80 °C with an initial concentration of substrates c = 0.1 M and catalyst loading 0.1, 0.25, 0.5 or 1 mol%. To a stirred solution of the substrate (1 equiv.) and durene (0.5-1 equiv., used as an internal standard) in a dry solvent, a solution of catalyst (**Ru2,**

[6] H. A. Flores Toque, F. B. M. Priviero, C. E. Teixeira, E. Perissutti, F. Fiorino, B. Severino, F. Frecentese, R. Lorenzetti, J. S. Baracat, V. Santagada, G. Caliendo, E. Antunes, G. De Nucci, *J. Med. Chem. 2008*, *51*, 2807-2815.

trans-**Ru6**, *cis*-**Ru6**) in dry solvent was added in a single portion at a given temperature. The reaction mixture was stirred for an appropriate time at the same temperature. Conversion for compounds **1a-e, 1j-k** and **14a** was determined by taking aliquot (0.1 mL) which was quenched immediately with an ice-cold solution of ethyl-vinyl ether (0.1 mL) and analyzed by GC, using GC EP Clarus 580 chromatograph with InertCap MS5/Sil column. Conversion for compounds **1f-g** and **13a** was determined by ^{1}H NMR (Agilent 400 MHz spectrometer).

Procedure D:

Comparative experiments with model substrates (**1a, 1h-g**, and **15a**) were performed in a dry perfluorinated toluene (PFT) under argon at 120 °C with an initial concentration of substrates c = 0.2 M and appropriate catalyst loading (1 or 2 mol%). Reaction was performed in 10 mL vial sealed by cap. In the argon filed glove-box, appropriate substrate was added to the vial containing the PFT (0.5–1 mL) followed by addition of catalyst (**Ru2,** ***trans*-Ru6, *cis*-Ru6** or **Ru8**). Next, the mixture was heated using microwave irradiation (200W) at 120 °C for 15 min. In case of portionwise addition of catalyst (2x2 mol%), after completion of first irradiation the vial was transferred to the glove-box to charge it with second portion of catalyst, followed by irradiation for another 15 min. (200 W, 120 °C). After completion the solvent was evaporated, internal standard was added (durene, 0.5 equiv.) and residue was dissolved in $CDCl_3$ (1-2 mL) to determine conversion by by ^{1}H NMR (Agilent 400 MHz spectrometer).

S3.2. Preparation of product 2g

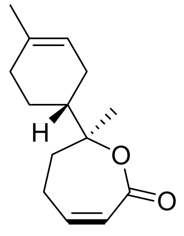

2g

Compound **2g** was obtained by adaptation of **Procedure D** by RCM reaction of **1g** (1 mmol) catalysed by *trans*-**Ru6** (1 mol%) and purified by column chromatography (SiO_2, hexane/EtOAc gradient, 100:0 to 80:20, v/v) to give 59.8 mg as colorless oil (27%). Formation of the main product **2g** was accompanied by formation of other (macrocyclic) products with similar polarity.

^{1}H NMR (400 MHz, $CDCl_3$): δ = 6.37 (dt, *J* = 12.3, 4.1 Hz, 1H), 6.02 (dt, *J* = 12.3, 2.1 Hz, 1H), 5.35 (dd, *J* = 3.9, 1.3 Hz, 1H), 2.65 – 2.35 (m, 2H), 2.16 – 1.80 (m, 8H), 1.63 (s, 3H), 1.46 – 1.27 (m, 4H) ppm; ^{13}C NMR (101 MHz, $CDCl_3$): δ = 167.3, 144.9, 134.3, 124.0, 119.9, 84.4, 42.9, 33.9, 30.9, 28.0, 27.1, 23.4, 23.4, 20.7 ppm; HRMS (ESI): *m*/*z*: calcd for $C_{14}H_{21}O_2$: 221.1536 $[M+H]^+$, found: 221.1539.

S3.3. Preparation of product 15b

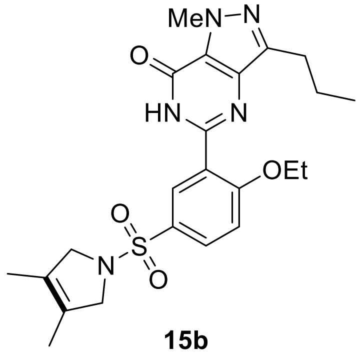

15b

Compound **15b** was obtained by adaptation of **Procedure D** by RCM reaction of **15a** (1.6 mmol) catalysed by *cis*-**Ru6** (2x0.5 mol%). Formed suspension was filtered off and the precipitate was washed with cold PFT (5 ml) and cold EtOAc (2 ml). Product **15b** was dissolved in the minimal amount of hot EtOAc and diluted with hot hexane until the precipitate was formed. After cooling down to rt the formed precipitate was filtered off and dried under vacum to afford the product **15b** as an colorless solid (635 mg, 84%).

^{1}H NMR (400 MHz, $CDCl_3$): δ = 10.84 (s, 1H), 8.83 (d, *J* = 2.4 Hz, 1H), 7.88 (dd, *J* = 8.7, 2.4 Hz, 1H), 7.13 (d, *J* = 8.8 Hz, 1H), 4.35 (q, *J* = 7.0 Hz, 2H), 4.25 (s, 3H), 4.02 (s, 4H), 2.92 (t, *J* = 7.5 Hz, 2H), 1.94 – 1.77 (m, 2H), 1.61 (t, *J* = 7.0 Hz, 3H), 1.54 (s, 6H), 1.01 (t, *J* = 7.4 Hz, 3H) ppm; ^{13}C NMR (101 MHz, $CDCl_3$): δ = 159.2, 153.7, 147.0, 146.8, 138.5, 131.5, 130.9, 130.7, 126.3, 124.6, 121.3, 113.1, 66.1, 59.1, 38.3, 27.8, 22.4, 14.7, 14.2, 11.2 ppm; HRMS (ESI): *m*/*z*: calcd for $C_{23}H_{30}N_5O_4S$: 472.20130 $[M+H]^+$, found: 472.20133.

S4. CM reactions

S4.1. General procedures

<u>Procedure E</u>

A comparative experiments with the model substrates (**11a**-**12a** and **S1**) were performed in dry solvent (DCM or C_7D_8) under argon at 40 or 80 °C with an initial concentration of model substrate c = 0.1 M and a catalyst loading 1 mol%. To a stirred solution of a model substrate (**11a**-**12a** or **S1**, 1 equiv.) and (*Z*)-1,4-diacetoxy-2-butene (**9a**) or *tert*-butyl acrylate (3 equiv.) in a appropriate solvent the solution of catalyst (**Ru2**, ***trans*-Ru6**, ***cis*-Ru6**) was added in a single portion at 40 or 80 °C. The reaction mixture was stirred for an appropriate time at a given temperature.

In the case of **S1**, aliquots (0.1 mL) were taken in regular intervals and quenched immediately with an ice-cold solution of ethyl-vinyl ether (0.1 mL) and analyzed by GC, using EP Clarus 580 chromatograph with InertCap MS5/Sil column with durene as an internal standard.

In the case of **11a**, after completion of the reaction internal standard was added (durene, 0.5 equiv.) and the yield of the product was determined by ^{1}H NMR (Agilent 400 MHz spectrometer).

In the case of **12a**, after completion of the reaction, the mixture was immediately cooled down to room temperature, ethyl vinyl ether was added, and the mixture was stirred for 15 minutes. Next, the solvent was evaporated, and the residue was purified by column chromatography (10% EtOAc/*c*-hexane), and isomers **12b** and **12b'** were isolated.

<u>Procedure F</u>

Comparative experiments for model substrates (**7a** and **9a**-**10a**) were performed in a dry perfluorinated toluene (PFT) under argon at 120 °C with an initial concentration of model substrates c = 0.2 M with compound **8a** (3 equiv.) and appropriate catalyst loading (1 or 2 mol%). Reaction was performed in 10 mL vial sealed by cap. In the argon filed glove-box, appropriate substrate was added to the vial containing the PFT (0.5 mL – 10 mL) followed by addition of catalyst (**Ru2**, ***trans*-Ru6**, ***cis*-Ru6** or **Ru8**). Next, the mixture was heated using microwave irradiation (200W) at 120 °C for 15 min. After completion the solvent was evaporated, internal standard was added (durene, 0.5 equiv.) and residue was dissolved in $CDCl_3$ (1-2 mL) to determine yield by ^{1}H NMR (Agilent 400 MHz spectrometer).

S4.2. Results for model CM reaction of allylbenzene (S1) with 8a

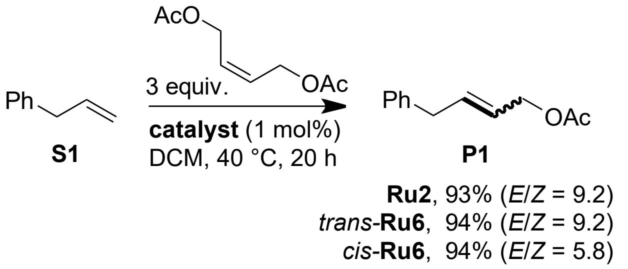

Scheme S1. Cross-metathesis for substrates **S1** and **8a**. Reactions perfomed according to **Procedure E**.

S4.3. Preparation of product 9b

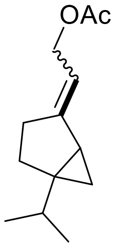

9b

Compound **9b** was obtained by adaptation of **Procedure F** by CM reaction of **15a** (1 mmol) with **8a** (3 mmol) catalysed by *trans*-**Ru6** (2 mol%) and purified by column chromatography (SiO_2, hexane/EtOAc gradient, 100:0 to 80:20, v/v) to give 196 mg as a colorless oil (94%). NMR reported to an equimolar mixture of E/Z isomers.

^{1}H NMR (400 MHz, $CDCl_3$): δ = 5.44 – 5.34 (m, 1H), 5.28 – 5.18 (m, 1H), 4.73 – 4.63 (m, 1H), 4.63 – 4.47 (m, 2H), 4.47 – 4.37 (m, 1H), 2.48 – 2.35 (m, 1H), 2.15 (m, 1H), 2.09 – 1.93 (m, 7H), 1.88 – 1.54 (m, 7H), 1.53 – 1.37 (m, 2H), 0.90 (d, *J* = 6.9 Hz, 6H), 0.84 (dd, *J* = 8.3, 6.9 Hz, 6H), 0.71 (dd, *J* = 7.9, 4.6 Hz, 1H), 0.68 – 0.59 (m, 2H), 0.54 (dd, *J* = 4.4, 3.4 Hz, 1H) ppm; ^{13}C NMR (101 MHz, $CDCl_3$): δ = 171.1, 171.0, 152.3, 152.2, 111.5, 111.1, 62.8, 62.5, 38.1, 36.9, 32.7, 32.4, 30.7, 29.7, 27.6, 26.5, 25.9, 24.5, 21.2, 21.1, 19.9, 19.8, 19.7, 19.7, 16.7, 16.5 ppm; HRMS (ESI): *m*/*z*: calcd for $C_{13}H_{20}O_2Na$: 231.1361 [M+Na]$^+$, found: 231.1354.

S5. DRRM reactions

S5.1. General procedure for DRRM

<u>Procedure G</u>

Comparative experiment with model substrate **S3** was performed in $CDCl_3$ saturated with ethylene at rt with the initial concentration of **S3** (c = 0.02 M, 1 equiv.), durene (0.5 equiv.), and catalyst loading 5 mol%. To a stirred solution of **S3**, a solution of catalyst (**Ru1, Ru2,** *trans*-**Ru6,** *cis*-**Ru6**) in $CDCl_3$ was added in a single portion at rt. The reaction mixture was left at rt for 1 hour and ethyl vinyl ether (100 µL) was added to the reaction mixture. The solution was analysed by GC chromatography, using EP Clarus 580 chromatograph with InertCap MS5/Sil column.

S3.1. Results for model DRRM reaction of *tert*-butyl((1-(cyclopent-3-en-1-yl)but-3-en-1-yl)oxy)dimethylsilane (S3)

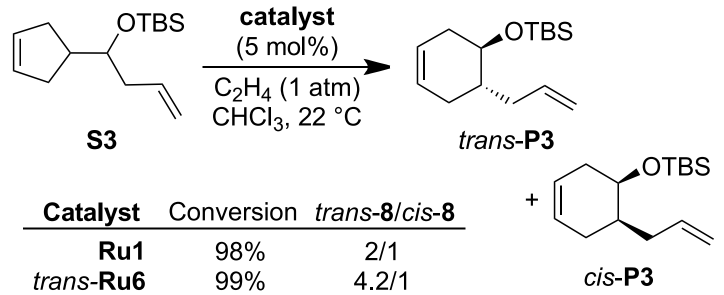

Catalyst	Conversion	*trans*-**8**/*cis*-**8**
**Ru1**	98%	2/1
trans-**Ru6**	99%	4.2/1
cis-**Ru6**	99%	9.1/1
**Ru2**	91%	15.4/1

Scheme S2. Diastereomeric ring-rearrangement metathesis for substrate S3. Reactions perfomed according to **Procedure G**.

S6. X-ray diffraction

Experiments were carried out at 100 K with Mo *Kα* radiation using a Kappa ApexII Ultra CCD. Absorption was corrected for by multi-scan methods, *SADABS2008*/1 - Bruker Nonius area detector scaling and absorption correction. CCDC 2087687 (cis-**Ru6**), 2087688 (trans-**Ru6**) and 2151885 (**Ru8**) contain the supplementary crystallographic data for this paper. These data are provided free of charge by The Cambridge Crystallographic Data Centre.

Table S1. Experimental details for SCXRD of *trans*-**Ru6** and *cis*-**Ru6**, **Ru8**.

	trans-**Ru6**	cis-**Ru6**	**Ru-8**
Crystal data			
Chemical formula	$C_{48}H_{67}Cl_2N_2PRu·CH_2Cl_2$	$C_{48}H_{67}Cl_2N_2PRu·3(CH_2Cl_2)$	$C_{33}H_{40}Cl_2N_2ORu·CH_4O$
M_r	959.90	1129.75	684.68
Crystal system, space group	Triclinic, $P^{-}1$	Monoclinic, $P2_1/n$	Triclinic, $P^{-}1$
a, *b*, *c* (Å)	9.6828 (9), 13.0687 (12), 19.1788 (17)	15.9530 (13), 21.6658 (17), 16.5514 (14)	10.6042 (7), 11.2650 (7), 15.0385 (10)
α, β, γ (°)	77.242 (3), 83.599 (3), 76.298 (2)	90, 109.213 (1), 90	83.636 (3), 84.196 (3), 64.031 (3)
V (Å^3)	2295.1 (4)	5402.1 (8)	1602.32 (18)
Z	2	4	2
(mm^{-1})	0.65	0.75	0.69
Crystal size (mm)	0.20 × 0.15 × 0.03	0.25 × 0.10 × 0.10	0.13 × 0.10 × 0.09
Data collection			
Diffractometer	Kappa ApexII Ultra CCD	Kappa ApexII Ultra CCD	CCD area detector
Absorption correction	Multi-scan *SADABS2008*/1 - Bruker Nonius area detector scaling and absorption correction	Multi-scan *SADABS2008*/1 - Bruker Nonius area detector scaling and absorption correction	Multi-scan Sheldrick, G. M. (1996). *SADABS*. University of Gttingen, Germany.
T_{min}, T_{max}	0.882, 0.981	0.834, 0.929	0.653, 0.745
No. of measured, independent and observed [*I* > 2 (*I*)] reflections	87428, 10944, 10007	75081, 10282, 8484	20710, 6137, 4567
R_{int}	0.026	0.042	0.061
(sin /)$_{max}$ (Å^{-1})	0.658	0.610	0.613
Refinement			
$R[F^2 > 2 (F^2)]$, $wR(F^2)$, S	0.029, 0.078, 1.06	0.033, 0.085, 1.05	0.042, 0.094, 0.96
No. of reflections	10944	10282	6137
No. of parameters	514	606	377
H-atom treatment	H-atom parameters constrained	H-atom parameters constrained	H atoms treated by a mixture of independent and constrained refinement

$\Delta\rho_{max}$, $\Delta\rho_{min}$ (e Å^{-3})	1.96, -1.29	1.68, -1.22	0.94, -0.74

Computer programs: Bruker *SMART*, Bruker *SAINT*, *SHELXS97* (Sheldrick, 1990), Bruker *SHELXTL*, *SHELXL97* (Sheldrick, 1997), *WinGX*, *Mercury*, *ORTEP-3 for Windows*: L. J. Farrugia, J. Appl. Cryst (1997), 30, 565.

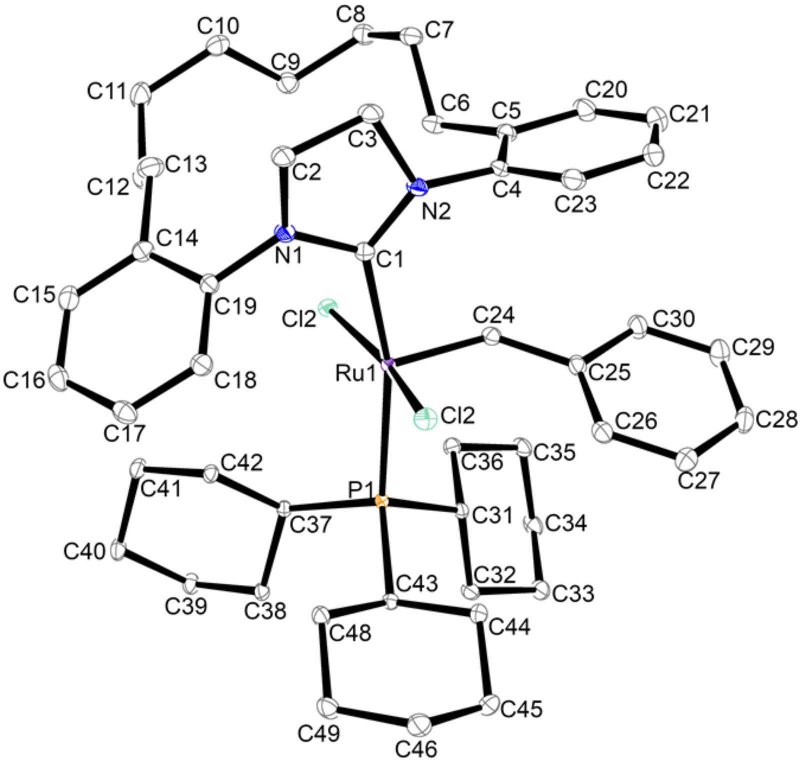

Figure S1. Molecular structure of *trans*-**Ru6** with atom labels. Ellipsoids are drawn at the 50% probability level. Hydrogen atoms and crystallisation solvent molecules are omitted for clarity.

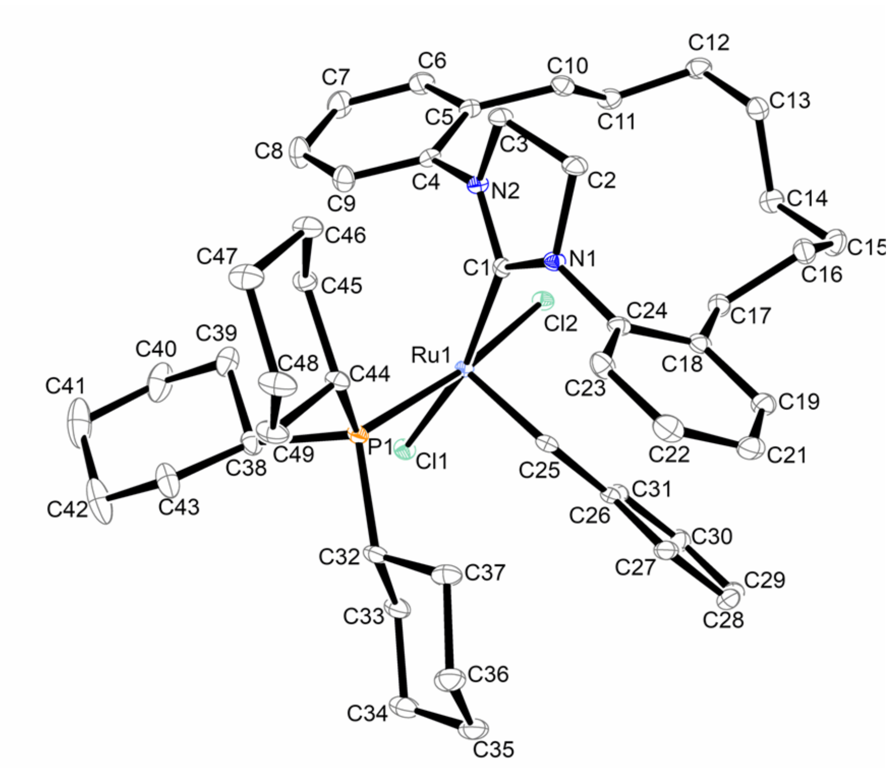

Figure S2. Molecular structure of *cis*-**Ru6** with atom labels. Ellipsoids are drawn at the 50% probability level. Hydrogen atoms and crystallisation solvent molecules are omitted for clarity.

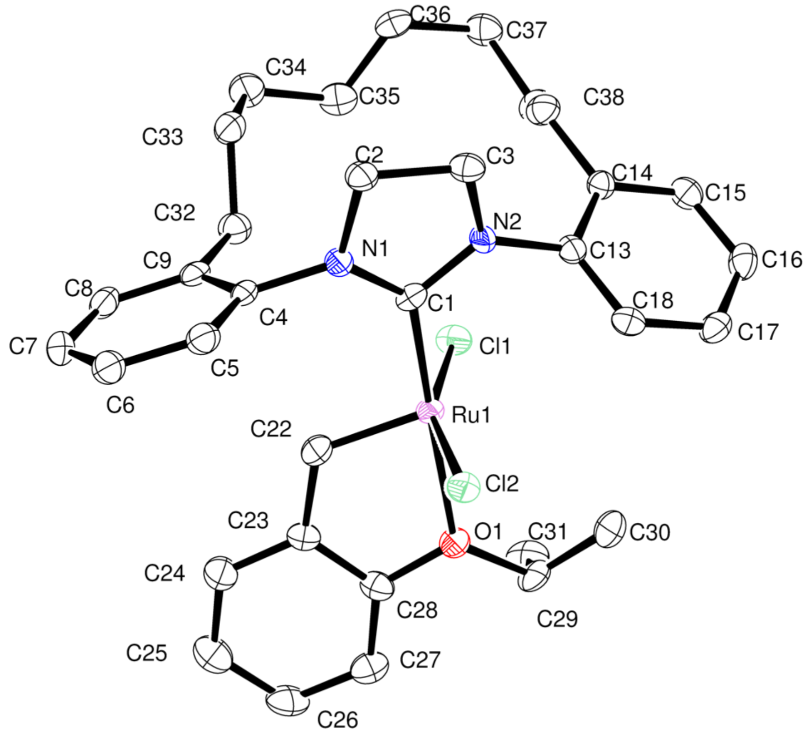

Figure S3. Molecular structure of **Ru8** with atom labels. Ellipsoids are drawn at the 50% probability level. Hydrogen atoms and crystallization solvent molecules are omitted for clarity

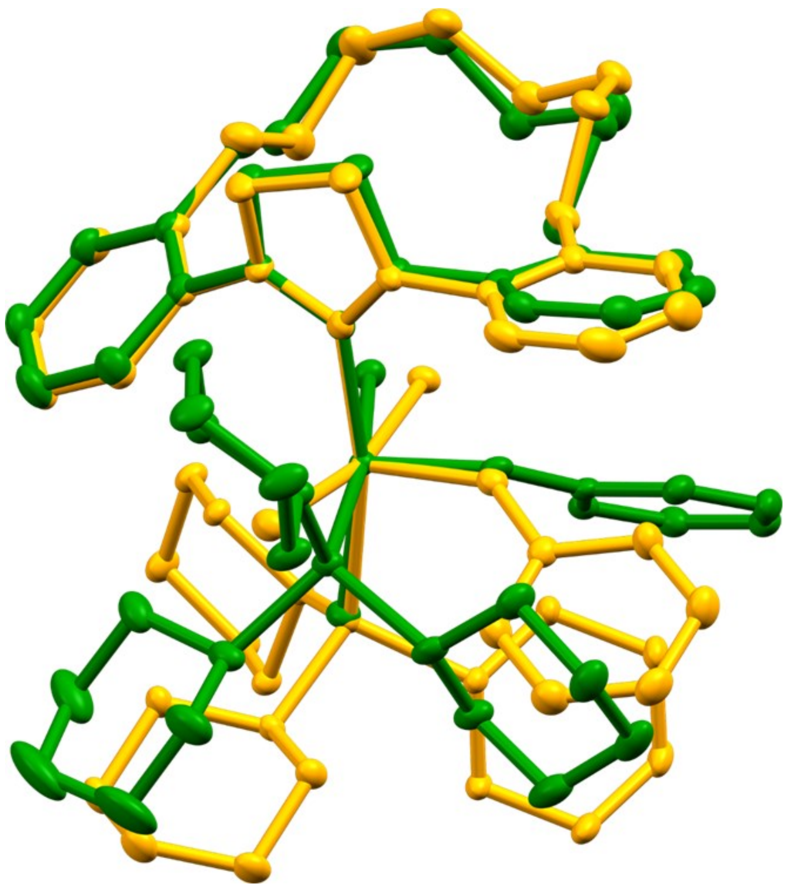

Figure S4. Structural overlay of molecules: *trans*-**Ru6** and *cis*-**Ru6**. Hydrogen atoms and crystallisation solvent molecules are omitted for clarity.

The structure of *trans*-**Ru6**, *cis*-**Ru6** and **Ru8** was confirmed by X-ray analysis (Table S1, Figures S1, S2, S3). Three ruthenium complexes adopt a characteristic distorted square bipyramid coordination around the central ruthenium atom. A top of these pyramids are the C1 carbon atoms of the NHC ligand and the second one is P1 phosphorus atom of the triphenylphosphine group for trans-Ru6 and Cl1 atom for cis-Ru6. Additionally, the overlay of the cis and trans molecules by Ru1, C1, and Cl2 atoms showed that the NHC ligand position is conserved, whereas benzylidine ring is flipped (Figure S4).

Table S2. Selected geometric parameters (Å, °)

trans-**Ru6**		*cis*-**Ru6**		**Ru8**	
Ru1—C24	1.8406 (18)	C2—N1	1.478 (3)	Ru1—C22	1.814 (4)
Ru1—C1	2.0559 (17)	C2—C3	1.510 (3)	Ru1—C1	1.955 (4)
Ru1—Cl2	2.4002 (5)	C3—N2	1.474 (3)	Ru1—O1	2.254 (3)
Ru1—Cl1	2.4036 (5)	C50—Cl5	1.717 (3)	Ru1—Cl1	2.3288 (10)
Ru1—P1	2.4638 (5)	C50—Cl6	1.725 (3)	Ru1—Cl2	2.3440 (10)
Cl3—C49	1.764 (2)	C51—Cl4A	1.693 (6)	O1—C28	1.373 (4)
Cl4—C49	1.756 (2)	C51—Cl3	1.750 (5)	O1—C29	1.474 (4)
P1—C43	1.8557 (17)	C51—Cl4B	1.784 (11)	N1—C1	1.354 (4)
P1—C37	1.8620 (17)	Ru1—C25	1.839 (2)	N1—C4	1.427 (4)
P1—C31	1.8681 (17)	Ru1—C1	2.041 (2)	N1—C2	1.470 (5)

N1—C1	1.348 (2)
N1—C19	1.434 (2)
N1—C2	1.472 (2)
N2—C1	1.346 (2)
N2—C4	1.434 (2)
N2—C3	1.478 (2)
C2—C3	1.520 (3)
C4—C23	1.389 (3)
C4—C5	1.398 (3)
C5—C20	1.397 (3)
C5—C6	1.508 (3)
C6—C7	1.532 (2)
C7—C8	1.532 (3)
C8—C9	1.531 (3)
C9—C10	1.526 (3)
C10—C11	1.534 (3)
C11—C12	1.536 (3)
C12—C13	1.523 (3)
C13—C14	1.512 (3)
C14—C15	1.395 (3)
C14—C19	1.405 (3)
C15—C16	1.386 (3)
C16—C17	1.382 (3)
C17—C18	1.397 (3)
C18—C19	1.386 (3)
C20—C21	1.378 (3)
C21—C22	1.377 (4)
C22—C23	1.389 (3)
C24—C25	1.471 (2)
C25—C26	1.391 (3)
C25—C30	1.407 (3)
C26—C27	1.389 (3)
C27—C28	1.389 (3)
C28—C29	1.383 (3)
C29—C30	1.393 (3)
C31—C36	1.537 (2)
C31—C32	1.537 (2)
C32—C33	1.531 (2)
C33—C34	1.525 (3)
C34—C35	1.522 (3)
C35—C36	1.529 (2)
C37—C38	1.536 (2)
C37—C42	1.539 (2)
C38—C39	1.533 (2)
C39—C40	1.525 (2)
C40—C41	1.525 (2)
C41—C42	1.530 (2)
C43—C44	1.537 (2)
C43—C48	1.538 (2)
C44—C45	1.529 (2)
C45—C46	1.526 (3)
C46—C47	1.528 (3)
C47—C48	1.531 (2)

Ru1—P1	2.3846 (7)
Ru1—Cl2	2.3908 (6)
Ru1—Cl1	2.4077 (6)
P1—C32	1.858 (2)
P1—C38	1.864 (3)
P1—C44	1.877 (3)
N2—C1	1.362 (3)
N2—C4	1.431 (3)
N1—C1	1.362 (3)
N1—C24	1.434 (3)
C31—C30	1.392 (4)
C31—C26	1.401 (3)
C30—C29	1.386 (4)
C13—C12	1.526 (4)
C13—C14	1.528 (4)
C37—C36	1.531 (4)
C37—C32	1.542 (3)
C6—C7	1.385 (4)
C6—C5	1.394 (4)
C4—C9	1.385 (4)
C4—C5	1.401 (4)
C23—C22	1.382 (4)
C23—C24	1.391 (4)
C48—C47	1.514 (4)
C48—C46	1.525 (4)
C22—C21	1.389 (4)
C26—C27	1.402 (4)
C26—C25	1.466 (3)
C10—C5	1.513 (4)
C10—C11	1.532 (4)
C19—C21	1.379 (4)
C19—C18	1.397 (4)
C24—C18	1.402 (4)
C18—C17	1.513 (4)
C27—C28	1.385 (4)
C14—C15	1.524 (4)
C15—C16	1.531 (4)
C17—C16	1.532 (4)
C38—C39	1.527 (4)
C38—C43	1.539 (4)
C45—C46	1.533 (4)
C45—C44	1.536 (4)
C9—C8	1.391 (4)
C32—C33	1.533 (3)
C33—C34	1.532 (3)
C34—C35	1.524 (4)
C28—C29	1.383 (4)
C44—C49	1.540 (3)
C49—C47	1.535 (4)
C35—C36	1.527 (4)
C7—C8	1.378 (4)
C39—C40	1.525 (4)
C11—C12	1.528 (4)
C41—C42	1.506 (6)
C41—C40	1.519 (5)
C43—C42	1.531 (5)
C52—Cl8A	1.735 (17)

N2—C1	1.347 (4)
N2—C13	1.433 (4)
N2—C3	1.467 (4)
C2—C3	1.507 (5)
C4—C5	1.383 (5)
C4—C9	1.394 (5)
C5—C6	1.375 (5)
C6—C7	1.373 (5)
C7—C8	1.379 (5)
C8—C9	1.391 (5)
C9—C32	1.509 (5)
C13—C18	1.383 (5)
C13—C14	1.391 (5)
C14—C15	1.391 (5)
C14—C39	1.505 (5)
C15—C16	1.377 (5)
C16—C17	1.374 (5)
C17—C18	1.386 (5)
C22—C23	1.438 (5)
C23—C28	1.394 (5)
C23—C24	1.399 (5)
C24—C25	1.370 (6)
C25—C26	1.391 (6)
C26—C27	1.380 (6)
C27—C28	1.377 (5)
C29—C30	1.497 (6)
C29—C31	1.509 (6)
C32—C33	1.514 (5)
C33—C34	1.523 (5)
C34—C35	1.523 (5)
C35—C36	1.506 (5)
C36—C37	1.518 (5)
C37—C38	1.524 (5)
C38—C39	1.520 (5)
O80—C80	1.336 (6)

		C52—Cl7A	1.793 (18)		
		Cl8B—C52B	1.719 (17)		
		C52B—Cl7B	1.69 (2)		
C24—Ru1—C1	96.36 (7)	N1—C2—C3	102.06 (19)	C22—Ru1—C1	102.71 (16)
C24—Ru1—Cl2	90.00 (6)	N2—C3—C2	101.15 (18)	C22—Ru1—O1	78.79 (14)
C1—Ru1—Cl2	88.48 (5)	Cl5—C50—Cl6	115.76 (19)	C1—Ru1—O1	174.22 (12)
C24—Ru1—Cl1	100.84 (6)	Cl4A—C51—Cl3	116.0 (5)	C22—Ru1—Cl1	100.48 (12)
C1—Ru1—Cl1	84.43 (5)	Cl4A—C51—Cl4B	22.2 (7)	C1—Ru1—Cl1	93.86 (11)
Cl2—Ru1—Cl1	167.623 (16)	Cl3—C51—Cl4B	102.7 (7)	O1—Ru1—Cl1	91.33 (7)
C24—Ru1—P1	96.97 (5)	C25—Ru1—C1	93.68 (10)	C22—Ru1—Cl2	99.32 (12)
C1—Ru1—P1	166.48 (5)	C25—Ru1—P1	88.07 (8)	C1—Ru1—Cl2	88.50 (10)
Cl2—Ru1—P1	89.292 (16)	C1—Ru1—P1	97.57 (7)	O1—Ru1—Cl2	85.75 (7)
Cl1—Ru1—P1	95.242 (16)	C25—Ru1—Cl2	101.24 (8)	Cl1—Ru1—Cl2	159.02 (3)
C43—P1—C37	104.47 (7)	C1—Ru1—Cl2	85.85 (7)	C28—O1—C29	118.9 (3)
C43—P1—C31	102.73 (7)	P1—Ru1—Cl2	169.89 (2)	C28—O1—Ru1	110.5 (2)
C37—P1—C31	105.96 (7)	C25—Ru1—Cl1	101.84 (8)	C29—O1—Ru1	129.7 (2)
C43—P1—Ru1	121.47 (6)	C1—Ru1—Cl1	163.76 (7)	C1—N1—C4	127.3 (3)
C37—P1—Ru1	109.18 (5)	P1—Ru1—Cl1	87.79 (2)	C1—N1—C2	113.7 (3)
C31—P1—Ru1	111.80 (5)	Cl2—Ru1—Cl1	86.47 (2)	C4—N1—C2	118.8 (3)
C1—N1—C19	126.39 (14)	C32—P1—C38	109.86 (12)	C1—N2—C13	126.1 (3)
C1—N1—C2	112.74 (15)	C32—P1—C44	102.26 (11)	C1—N2—C3	113.5 (3)
C19—N1—C2	119.95 (14)	C38—P1—C44	100.12 (12)	C13—N2—C3	119.5 (3)
C1—N2—C4	128.01 (15)	C32—P1—Ru1	116.62 (8)	N2—C1—N1	106.2 (3)
C1—N2—C3	112.40 (15)	C38—P1—Ru1	109.89 (9)	N2—C1—Ru1	120.3 (2)
C4—N2—C3	116.70 (14)	C44—P1—Ru1	116.67 (8)	N1—C1—Ru1	132.8 (3)
N2—C1—N1	107.17 (15)	C1—N2—C4	128.2 (2)	N1—C2—C3	102.5 (3)
N2—C1—Ru1	131.58 (13)	C1—N2—C3	113.10 (19)	N2—C3—C2	102.6 (3)
N1—C1—Ru1	120.93 (12)	C4—N2—C3	118.46 (19)	C5—C4—C9	121.7 (3)
N1—C2—C3	101.11 (14)	C1—N1—C24	129.0 (2)	C5—C4—N1	118.4 (3)
N2—C3—C2	101.87 (14)	C1—N1—C2	112.51 (19)	C9—C4—N1	119.8 (3)
C23—C4—C5	121.40 (17)	C24—N1—C2	114.08 (19)	C6—C5—C4	120.2 (3)
C23—C4—N2	118.07 (17)	C30—C31—C26	120.1 (2)	C7—C6—C5	119.4 (4)
C5—C4—N2	120.21 (16)	C29—C30—C31	120.5 (2)	C6—C7—C8	120.3 (4)
C20—C5—C4	117.19 (18)	C12—C13—C14	114.4 (2)	C7—C8—C9	121.9 (3)
C20—C5—C6	119.36 (17)	C36—C37—C32	110.0 (2)	C8—C9—C4	116.5 (3)
C4—C5—C6	123.37 (16)	C7—C6—C5	122.0 (3)	C8—C9—C32	120.0 (3)
C5—C6—C7	115.44 (15)	C9—C4—C5	121.1 (2)	C4—C9—C32	123.5 (3)
C8—C7—C6	112.09 (16)	C9—C4—N2	119.6 (2)	C18—C13—C14	121.4 (3)
C7—C8—C9	115.63 (16)	C5—C4—N2	119.3 (2)	C18—C13—N2	119.7 (3)
C10—C9—C8	113.70 (17)	C22—C23—C24	120.5 (3)	C14—C13—N2	118.8 (3)
C9—C10—C11	113.53 (18)	C47—C48—C46	110.5 (2)	C13—C14—C15	117.3 (3)
C10—C11—C12	114.62 (17)	C23—C22—C21	119.3 (3)	C13—C14—C39	122.5 (3)
C13—C12—C11	112.66 (18)	N1—C1—N2	105.8 (2)	C15—C14—C39	120.2 (3)
C14—C13—C12	113.93 (17)	N1—C1—Ru1	132.66 (17)	C16—C15—C14	121.6 (4)
C15—C14—C19	117.52 (17)	N2—C1—Ru1	121.49 (17)	C17—C16—C15	120.4 (3)
C15—C14—C13	120.11 (18)	C31—C26—C27	118.3 (2)	C16—C17—C18	119.4 (4)
C19—C14—C13	122.36 (17)	C31—C26—C25	125.0 (2)	C17—C18—C13	120.0 (3)
C16—C15—C14	121.76 (18)	C27—C26—C25	116.7 (2)	C23—C22—Ru1	119.6 (3)
C17—C16—C15	119.68 (18)	C5—C10—C11	115.1 (2)	C28—C23—C24	118.1 (4)
C16—C17—C18	120.18 (18)	C21—C19—C18	122.3 (3)	C28—C23—C22	118.5 (3)
C19—C18—C17	119.51 (18)	C23—C24—C18	121.1 (2)	C24—C23—C22	123.4 (4)
C18—C19—C14	121.32 (17)	C23—C24—N1	117.6 (2)	C25—C24—C23	121.0 (4)
C18—C19—N1	119.64 (16)	C18—C24—N1	121.0 (2)	C24—C25—C26	119.1 (4)
C14—C19—N1	118.92 (16)	C19—C18—C24	116.9 (2)	C27—C26—C25	121.6 (4)
C21—C20—C5	121.7 (2)	C19—C18—C17	118.8 (2)	C28—C27—C26	118.4 (4)
C22—C21—C20	120.2 (2)	C24—C18—C17	124.3 (2)	O1—C28—C27	125.8 (4)
C21—C22—C23	119.8 (2)	C28—C27—C26	121.4 (2)	O1—C28—C23	112.4 (3)

C22—C23—C4	119.67 (19)
C25—C24—Ru1	135.35 (13)
C26—C25—C30	117.75 (17)
C26—C25—C24	124.57 (16)
C30—C25—C24	117.65 (16)
C27—C26—C25	120.93 (18)
C26—C27—C28	120.45 (19)
C29—C28—C27	119.91 (18)
C28—C29—C30	119.48 (19)
C29—C30—C25	121.46 (18)
C36—C31—C32	109.02 (14)
C36—C31—P1	112.01 (11)
C32—C31—P1	117.76 (11)
C33—C32—C31	110.84 (14)
C34—C33—C32	111.73 (15)
C35—C34—C33	110.85 (15)
C34—C35—C36	111.47 (14)
C35—C36—C31	110.69 (14)
C38—C37—C42	109.03 (13)
C38—C37—P1	116.93 (11)
C42—C37—P1	112.38 (11)
C39—C38—C37	110.20 (14)
C40—C39—C38	111.33 (14)
C41—C40—C39	111.05 (15)
C40—C41—C42	112.00 (14)
C41—C42—C37	110.72 (13)
C44—C43—C48	109.26 (14)
C44—C43—P1	112.19 (11)
C48—C43—P1	113.81 (11)
C45—C44—C43	111.23 (14)
C46—C45—C44	111.13 (15)
C45—C46—C47	111.32 (16)
C46—C47—C48	111.78 (15)
C47—C48—C43	110.70 (14)
Cl4—C49—Cl3	109.98 (12)

C19—C21—C22	119.9 (3)
C15—C14—C13	113.0 (2)
C14—C15—C16	115.3 (2)
C18—C17—C16	113.7 (2)
C39—C38—C43	109.3 (2)
C39—C38—P1	114.40 (18)
C43—C38—P1	118.3 (2)
C46—C45—C44	110.8 (2)
C4—C9—C8	120.5 (3)
C6—C5—C4	117.1 (2)
C6—C5—C10	121.3 (2)
C4—C5—C10	121.6 (2)
C33—C32—C37	108.3 (2)
C33—C32—P1	113.51 (17)
C37—C32—P1	118.33 (17)
C26—C25—Ru1	130.40 (19)
C15—C16—C17	112.5 (2)
C34—C33—C32	111.4 (2)
C35—C34—C33	111.4 (2)
C48—C46—C45	110.3 (2)
C29—C28—C27	119.6 (2)
C28—C29—C30	120.2 (2)
C45—C44—C49	110.6 (2)
C45—C44—P1	115.47 (17)
C49—C44—P1	112.85 (18)
C47—C49—C44	111.4 (2)
C34—C35—C36	110.9 (2)
C8—C7—C6	120.1 (3)
C7—C8—C9	119.2 (3)
C40—C39—C38	110.2 (3)
C12—C11—C10	112.4 (2)
C48—C47—C49	111.9 (2)
C13—C12—C11	116.0 (2)
C35—C36—C37	111.0 (2)
C42—C41—C40	112.1 (3)
C42—C43—C38	109.4 (3)
C41—C40—C39	111.8 (3)
C41—C42—C43	111.8 (3)
Cl8A—C52—Cl7A	107.4 (13)
Cl7B—C52B—Cl8B	115.3 (12)

C27—C28—C23	121.8 (4)
O1—C29—C30	105.6 (3)
O1—C29—C31	109.3 (3)
C30—C29—C31	112.7 (4)
C9—C32—C33	113.9 (3)
C32—C33—C34	112.7 (3)
C33—C34—C35	114.8 (3)
C36—C35—C34	112.4 (3)
C35—C36—C37	115.2 (3)
C36—C37—C38	114.6 (3)
C39—C38—C37	112.4 (3)
C14—C39—C38	114.3 (3)

C24—Ru1—P1—C43	108.16 (8)
C1—Ru1—P1—C43	−81.4 (2)
Cl2—Ru1—P1—C43	−161.93 (6)
Cl1—Ru1—P1—C43	6.54 (6)
C24—Ru1—P1—C37	−130.30 (8)
C1—Ru1—P1—C37	40.1 (2)
Cl2—Ru1—P1—C37	−40.38 (6)
Cl1—Ru1—P1—C37	128.08 (6)
C24—Ru1—P1—C31	−13.39 (8)
C1—Ru1—P1—C31	157.0 (2)
Cl2—Ru1—P1—C31	76.53 (6)
Cl1—Ru1—P1—C31	−115.00 (6)
C4—N2—C1—N1	−164.58 (16)
C3—N2—C1—N1	−4.7 (2)
C4—N2—C1—Ru1	22.0 (3)
C3—N2—C1—Ru1	−178.10 (13)
C19—N1—C1—N2	−179.22 (16)

N1—C2—C3—N2	−21.7 (2)
C25—Ru1—P1—C32	92.65 (12)
C1—Ru1—P1—C32	−0.80 (12)
Cl2—Ru1—P1—C32	−110.01 (15)
Cl1—Ru1—P1—C32	−165.41 (10)
C25—Ru1—P1—C38	−141.51 (12)
C1—Ru1—P1—C38	125.03 (11)
Cl2—Ru1—P1—C38	15.82 (17)
Cl1—Ru1—P1—C38	−39.58 (9)
C25—Ru1—P1—C44	−28.51 (12)
C1—Ru1—P1—C44	−121.97 (12)
Cl2—Ru1—P1—C44	128.82 (15)
Cl1—Ru1—P1—C44	73.42 (10)
C2—C3—N2—C1	20.5 (3)
C2—C3—N2—C4	−164.8 (2)
C3—C2—N1—C1	18.8 (3)
C3—C2—N1—C24	177.4 (2)

C22—Ru1—O1—C28	−4.3 (2)
Cl1—Ru1—O1—C28	−104.7 (2)
Cl2—Ru1—O1—C28	96.1 (2)
C22—Ru1—O1—C29	164.7 (3)
Cl1—Ru1—O1—C29	64.3 (3)
Cl2—Ru1—O1—C29	−94.9 (3)
C13—N2—C1—N1	178.7 (3)
C3—N2—C1—N1	10.1 (4)
C13—N2—C1—Ru1	7.4 (5)
C3—N2—C1—Ru1	−161.2 (2)
C4—N1—C1—N2	171.4 (3)
C2—N1—C1—N2	−2.7 (4)
C4—N1—C1—Ru1	−18.9 (5)
C2—N1—C1—Ru1	167.0 (3)
C22—Ru1—C1—N2	−171.3 (3)
Cl1—Ru1—C1—N2	−69.6 (3)
Cl2—Ru1—C1—N2	89.5 (3)

C2—N1—C1—N2	−10.3 (2)	C26—C31—C30—C29	1.3 (4)	C22—Ru1—C1—N1	20.1 (4)
C19—N1—C1—Ru1	−5.0 (2)	C1—N2—C4—C9	68.6 (3)	Cl1—Ru1—C1—N1	121.8 (3)
C2—N1—C1—Ru1	163.90 (12)	C3—N2—C4—C9	−105.1 (3)	Cl2—Ru1—C1—N1	−79.1 (3)
C24—Ru1—C1—N2	−17.92 (17)	C1—N2—C4—C5	−114.7 (3)	C1—N1—C2—C3	−5.0 (4)
Cl2—Ru1—C1—N2	−107.76 (16)	C3—N2—C4—C5	71.6 (3)	C4—N1—C2—C3	−179.7 (3)
Cl1—Ru1—C1—N2	82.40 (16)	C24—C23—C22—C21	1.0 (4)	C1—N2—C3—C2	−12.9 (4)
P1—Ru1—C1—N2	171.64 (12)	C24—N1—C1—N2	−161.2 (2)	C13—N2—C3—C2	177.7 (3)
C24—Ru1—C1—N1	169.44 (14)	C2—N1—C1—N2	−6.5 (3)	N1—C2—C3—N2	9.9 (3)
Cl2—Ru1—C1—N1	79.60 (14)	C24—N1—C1—Ru1	17.6 (4)	C1—N1—C4—C5	111.0 (4)
Cl1—Ru1—C1—N1	−90.24 (14)	C2—N1—C1—Ru1	172.30 (18)	C2—N1—C4—C5	−75.2 (4)
P1—Ru1—C1—N1	−1.0 (3)	C4—N2—C1—N1	176.5 (2)	C1—N1—C4—C9	−72.5 (5)
C1—N1—C2—C3	20.0 (2)	C3—N2—C1—N1	−9.5 (3)	C2—N1—C4—C9	101.4 (4)
C19—N1—C2—C3	−170.34 (16)	C4—N2—C1—Ru1	−2.5 (3)	C9—C4—C5—C6	1.5 (6)
C1—N2—C3—C2	16.6 (2)	C3—N2—C1—Ru1	171.53 (16)	N1—C4—C5—C6	178.0 (3)
C4—N2—C3—C2	178.95 (15)	C25—Ru1—C1—N1	−15.7 (2)	C4—C5—C6—C7	−0.3 (6)
N1—C2—C3—N2	−20.24 (18)	P1—Ru1—C1—N1	72.9 (2)	C5—C6—C7—C8	−0.4 (6)
C1—N2—C4—C23	−110.7 (2)	Cl2—Ru1—C1—N1	−116.7 (2)	C6—C7—C8—C9	−0.1 (6)
C3—N2—C4—C23	90.1 (2)	Cl1—Ru1—C1—N1	−178.64 (13)	C7—C8—C9—C4	1.3 (5)
C1—N2—C4—C5	75.6 (2)	C25—Ru1—C1—N2	162.98 (19)	C7—C8—C9—C32	179.5 (3)
C3—N2—C4—C5	−83.5 (2)	P1—Ru1—C1—N2	−108.48 (18)	C5—C4—C9—C8	−1.9 (5)
C23—C4—C5—C20	0.4 (3)	Cl2—Ru1—C1—N2	61.96 (18)	N1—C4—C9—C8	−178.4 (3)
N2—C4—C5—C20	173.86 (16)	Cl1—Ru1—C1—N2	0.0 (4)	C5—C4—C9—C32	179.8 (3)
C23—C4—C5—C6	177.34 (16)	C30—C31—C26—C27	−1.5 (4)	N1—C4—C9—C32	3.4 (5)
N2—C4—C5—C6	−9.2 (3)	C30—C31—C26—C25	177.7 (2)	C1—N2—C13—C18	−66.8 (5)
C20—C5—C6—C7	−67.9 (2)	C22—C23—C24—C18	−2.8 (4)	C3—N2—C13—C18	101.1 (4)
C4—C5—C6—C7	115.2 (2)	C22—C23—C24—N1	−176.3 (2)	C1—N2—C13—C14	117.3 (4)
C5—C6—C7—C8	159.80 (16)	C1—N1—C24—C23	−116.5 (3)	C3—N2—C13—C14	−74.8 (4)
C6—C7—C8—C9	57.8 (2)	C2—N1—C24—C23	89.1 (3)	C18—C13—C14—C15	−0.9 (5)
C7—C8—C9—C10	74.0 (2)	C1—N1—C24—C18	69.9 (3)	N2—C13—C14—C15	174.9 (3)
C8—C9—C10—C11	−179.43 (17)	C2—N1—C24—C18	−84.4 (3)	C18—C13—C14—C39	179.7 (3)
C9—C10—C11—C12	68.9 (3)	C21—C19—C18—C24	−1.1 (4)	N2—C13—C14—C39	−4.5 (5)
C10—C11—C12—C13	62.8 (2)	C21—C19—C18—C17	−178.8 (2)	C13—C14—C15—C16	0.4 (5)
C11—C12—C13—C14	173.66 (17)	C23—C24—C18—C19	2.8 (4)	C39—C14—C15—C16	179.8 (3)
C12—C13—C14—C15	−59.9 (2)	N1—C24—C18—C19	176.1 (2)	C14—C15—C16—C17	0.7 (6)
C12—C13—C14—C19	120.8 (2)	C23—C24—C18—C17	−179.6 (2)	C15—C16—C17—C18	−1.3 (6)
C19—C14—C15—C16	−0.7 (3)	N1—C24—C18—C17	−6.3 (4)	C16—C17—C18—C13	0.8 (5)
C13—C14—C15—C16	−179.98 (18)	C31—C26—C27—C28	0.8 (4)	C14—C13—C18—C17	0.3 (5)
C14—C15—C16—C17	−0.9 (3)	C25—C26—C27—C28	−178.4 (2)	N2—C13—C18—C17	−175.5 (3)
C15—C16—C17—C18	1.9 (3)	C18—C19—C21—C22	−0.6 (4)	C1—Ru1—C22—C23	−171.0 (3)
C16—C17—C18—C19	−1.5 (3)	C23—C22—C21—C19	0.7 (4)	O1—Ru1—C22—C23	3.3 (3)
C17—C18—C19—C14	−0.1 (3)	C12—C13—C14—C15	179.1 (2)	Cl1—Ru1—C22—C23	92.6 (3)
C17—C18—C19—N1	175.83 (16)	C13—C14—C15—C16	66.2 (3)	Cl2—Ru1—C22—C23	−80.4 (3)
C15—C14—C19—C18	1.1 (3)	C19—C18—C17—C16	−61.4 (3)	Ru1—C22—C23—C28	−2.1 (5)
C13—C14—C19—C18	−179.57 (17)	C24—C18—C17—C16	121.1 (3)	Ru1—C22—C23—C24	176.1 (3)
C15—C14—C19—N1	−174.83 (16)	C32—P1—C38—C39	83.6 (2)	C28—C23—C24—C25	0.0 (6)
C13—C14—C19—N1	4.5 (3)	C44—P1—C38—C39	−169.28 (19)	C22—C23—C24—C25	−178.2 (4)
C1—N1—C19—C18	67.4 (2)	Ru1—P1—C38—C39	−46.0 (2)	C23—C24—C25—C26	1.1 (7)
C2—N1—C19—C18	−100.7 (2)	C32—P1—C38—C43	−47.4 (2)	C24—C25—C26—C27	−1.3 (7)
C1—N1—C19—C14	−116.5 (2)	C44—P1—C38—C43	59.7 (2)	C25—C26—C27—C28	0.4 (6)
C2—N1—C19—C14	75.3 (2)	Ru1—P1—C38—C43	−177.02 (19)	C29—O1—C28—C27	14.6 (5)
C4—C5—C20—C21	−0.4 (3)	C5—C4—C9—C8	1.8 (4)	Ru1—O1—C28—C27	−175.0 (3)
C6—C5—C20—C21	−177.40 (19)	N2—C4—C9—C8	178.5 (2)	C29—O1—C28—C23	−166.1 (3)
C5—C20—C21—C22	0.4 (3)	C7—C6—C5—C4	−1.1 (4)	Ru1—O1—C28—C23	4.3 (4)
C20—C21—C22—C23	−0.4 (3)	C7—C6—C5—C10	178.6 (3)	C26—C27—C28—O1	−180.0 (4)
C21—C22—C23—C4	0.5 (3)	C9—C4—C5—C6	0.0 (4)	C26—C27—C28—C23	0.8 (6)
C5—C4—C23—C22	−0.5 (3)	N2—C4—C5—C6	−176.7 (2)	C24—C23—C28—O1	179.7 (3)
N2—C4—C23—C22	−174.07 (17)	C9—C4—C5—C10	−179.7 (2)	C22—C23—C28—O1	−2.1 (5)

C1—Ru1—C24—C25	115.04 (18)	N2—C4—C5—C10	3.6 (3)	C24—C23—C28—C27	−1.0 (6)
Cl2—Ru1—C24—C25	−156.49 (18)	C11—C10—C5—C6	−46.1 (3)	C22—C23—C28—C27	177.3 (4)
Cl1—Ru1—C24—C25	29.53 (18)	C11—C10—C5—C4	133.6 (2)	C28—O1—C29—C30	−167.2 (3)
P1—Ru1—C24—C25	−67.20 (18)	C36—C37—C32—C33	−60.0 (3)	Ru1—O1—C29—C30	24.6 (4)
Ru1—C24—C25—C26	1.0 (3)	C36—C37—C32—P1	169.1 (2)	C28—O1—C29—C31	71.3 (4)
Ru1—C24—C25—C30	178.95 (15)	C38—P1—C32—C33	−65.5 (2)	Ru1—O1—C29—C31	−97.0 (3)
C30—C25—C26—C27	−1.6 (3)	C44—P1—C32—C33	−171.16 (18)	C8—C9—C32—C33	94.4 (4)
C24—C25—C26—C27	176.33 (18)	Ru1—P1—C32—C33	60.3 (2)	C4—C9—C32—C33	−87.4 (4)
C25—C26—C27—C28	0.3 (3)	C38—P1—C32—C37	63.0 (2)	C9—C32—C33—C34	−165.4 (3)
C26—C27—C28—C29	1.3 (3)	C44—P1—C32—C37	−42.6 (2)	C32—C33—C34—C35	−70.1 (5)
C27—C28—C29—C30	−1.6 (3)	Ru1—P1—C32—C37	−171.10 (17)	C33—C34—C35—C36	−88.6 (4)
C28—C29—C30—C25	0.2 (3)	C31—C26—C25—Ru1	2.2 (4)	C34—C35—C36—C37	179.2 (3)
C26—C25—C30—C29	1.4 (3)	C27—C26—C25—Ru1	−178.60 (19)	C35—C36—C37—C38	−61.0 (5)
C24—C25—C30—C29	−176.70 (18)	C1—Ru1—C25—C26	−128.6 (2)	C36—C37—C38—C39	−55.9 (5)
C43—P1—C31—C36	167.99 (12)	P1—Ru1—C25—C26	134.0 (2)	C13—C14—C39—C38	−126.9 (4)
C37—P1—C31—C36	58.66 (13)	Cl2—Ru1—C25—C26	−42.1 (2)	C15—C14—C39—C38	53.7 (5)
Ru1—P1—C31—C36	−60.18 (12)	Cl1—Ru1—C25—C26	46.6 (2)	C37—C38—C39—C14	−176.5 (3)
C43—P1—C31—C32	40.40 (15)	C14—C15—C16—C17	60.4 (3)		
C37—P1—C31—C32	−68.93 (14)	C18—C17—C16—C15	160.8 (2)		
Ru1—P1—C31—C32	172.23 (11)	C37—C32—C33—C34	58.6 (3)		
C36—C31—C32—C33	57.82 (19)	P1—C32—C33—C34	−167.88 (18)		
P1—C31—C32—C33	−173.17 (13)	C32—C33—C34—C35	−56.3 (3)		
C31—C32—C33—C34	−56.6 (2)	C47—C48—C46—C45	−58.5 (3)		
C32—C33—C34—C35	54.4 (2)	C44—C45—C46—C48	58.6 (3)		
C33—C34—C35—C36	−54.9 (2)	C26—C27—C28—C29	0.0 (4)		
C34—C35—C36—C31	57.6 (2)	C27—C28—C29—C30	−0.3 (4)		
C32—C31—C36—C35	−58.37 (18)	C31—C30—C29—C28	−0.4 (4)		
P1—C31—C36—C35	169.50 (12)	C46—C45—C44—C49	−56.0 (3)		
C43—P1—C37—C38	−35.94 (14)	C46—C45—C44—P1	174.36 (18)		
C31—P1—C37—C38	72.15 (13)	C32—P1—C44—C45	−48.9 (2)		
Ru1—P1—C37—C38	−167.29 (11)	C38—P1—C44—C45	−161.95 (19)		
C43—P1—C37—C42	91.27 (12)	Ru1—P1—C44—C45	79.6 (2)		
C31—P1—C37—C42	−160.64 (11)	C32—P1—C44—C49	−177.40 (19)		
Ru1—P1—C37—C42	−40.08 (12)	C38—P1—C44—C49	69.5 (2)		
C42—C37—C38—C39	59.43 (18)	Ru1—P1—C44—C49	−48.9 (2)		
P1—C37—C38—C39	−171.74 (11)	C45—C44—C49—C47	53.6 (3)		
C37—C38—C39—C40	−58.27 (19)	P1—C44—C49—C47	−175.39 (19)		
C38—C39—C40—C41	54.7 (2)	C33—C34—C35—C36	53.9 (3)		
C39—C40—C41—C42	−53.6 (2)	C5—C6—C7—C8	0.4 (4)		
C40—C41—C42—C37	55.95 (19)	C6—C7—C8—C9	1.4 (5)		
C38—C37—C42—C41	−58.28 (18)	C4—C9—C8—C7	−2.5 (4)		
P1—C37—C42—C41	170.40 (11)	C43—C38—C39—C40	−59.7 (3)		
C37—P1—C43—C44	171.11 (12)	P1—C38—C39—C40	164.99 (19)		
C31—P1—C43—C44	60.66 (13)	C5—C10—C11—C12	174.9 (2)		
Ru1—P1—C43—C44	−65.12 (13)	C46—C48—C47—C49	56.6 (3)		
C37—P1—C43—C48	−64.20 (13)	C44—C49—C47—C48	−54.4 (3)		
C31—P1—C43—C48	−174.65 (12)	C14—C13—C12—C11	64.9 (3)		
Ru1—P1—C43—C48	59.57 (13)	C10—C11—C12—C13	60.2 (3)		
C48—C43—C44—C45	58.24 (19)	C34—C35—C36—C37	−55.8 (3)		
P1—C43—C44—C45	−174.59 (13)	C32—C37—C36—C35	59.5 (3)		
C43—C44—C45—C46	−56.9 (2)	C39—C38—C43—C42	60.0 (3)		
C44—C45—C46—C47	54.3 (2)	P1—C38—C43—C42	−166.7 (3)		
C45—C46—C47—C48	−54.3 (2)	C42—C41—C40—C39	−52.8 (4)		
C46—C47—C48—C43	56.3 (2)	C38—C39—C40—C41	56.0 (4)		
C44—C43—C48—C47	−57.58 (18)	C40—C41—C42—C43	53.5 (4)		
P1—C43—C48—C47	176.17 (12)	C38—C43—C42—C41	−57.1 (4)		

S7. CSD survey

A survey through CSD crystal structures database was made to observe any possible differences in NHC conformation between the newly obtained catalysts *trans*- and *cis*-**Ru6** and known Ru-complexes featuring NHC ligands bearing aryl arms with at least one *ortho* position free.[7]

The CSD (Version 5.41 plus Mar, May, and Aug 2010 updates) was surveyed for structures drawn explicitly in a query shown in Figure S5, with filters applied for coordinates determined, no disorder, absence of errors, and only single-crystal X-ray diffraction data. A total of 13 different crystal structures were found.

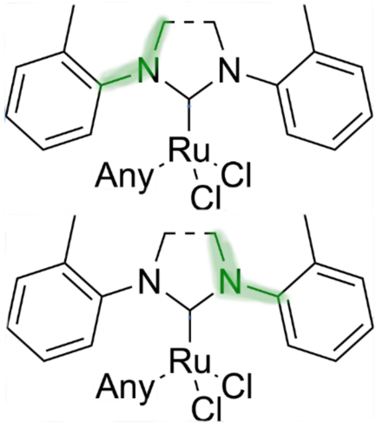

Figure S5. CSD queries with α_1 and α_2 valence angle definition (marked in green).

To compare structural features of NHC-Ru fragment in the reported solid state structures a specific valence angle (α_1 and α_2) was defined (Figure S5). The α_1 and α_2 values are listed in Table S3. Similar to the molecular structure of *trans*- and *cis*-**Ru6**, the methyl substituents in the *o*-tolil group are in the *syn* conformation except for Grubbs *o*-tolil derivative where both, *syn* and *anti,* conformations are present and α_1 and α_2 angles are 119.5(1)° and 119.8(1)°.[8] The average of α_1 and α_2 valence angle is 120(1)° and 121(1)°, whereas these angles are significantly smaller for *trans*-**Ru6** (114.1(2)° and 118.5(2)°) and for *cis*-**Ru6** (116.7(1)° and 119.9(2)°). Even smaller α_1 valence angle was found for pyridine indenylidene derivative[9] of 115.5(1)°, however, then α_2 is 121.4(2)°. Introduction of the aliphatic chain connecting the aryl groups in the NHC ligand forces the *syn* conformation and blocks the rotation.

---

[7] C. R. Groom, I. J. Bruno, M. P. Lightfoot, S. C. Ward, The Cambridge Structural Database. *Acta Crystallogr. Sect. B* **2016**, *72*, 171–179.

[8] I. C. Stewart, T. Ung, A. A. Pletnev, J. M. Berlin, R. H. Grubbs, Y. Schrodi, *Org. Lett.* **2007**, *9*, 1589–1592.

[9] C. Torborg, G. Szczepaniak, A. Zielinski, M. Malinska, K. Wozniak, K. Grela, *Chem. Commun.* **2013**, *49*, 3188–3190.

Table S3. Geometric parameters for selected molecules found in CSD (1-14) and new complexes *trans*-**Ru6** and *cis*-**Ru6**.

Index	Structure	NAME	Query	Fragment	α1	α2
1	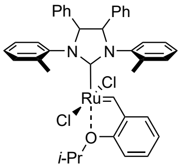	CIFWIZ	1	1	124.202	119.055
2	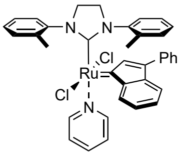	COGKEQ	1	1	119.525	122.566
3	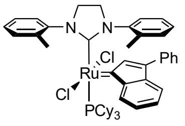	COGKIU	1	1	115.502	121.468
4	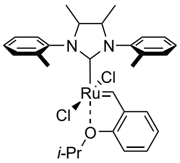	EXEVAF	1	1	122.027	120.085
5	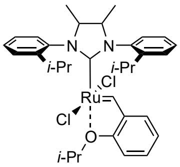	EXEVEJ	1	1	121.011	119.275
6[a]	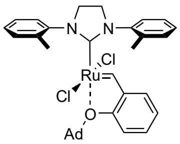	HIGGIQ	1	1	120.219	119.926
7[a]	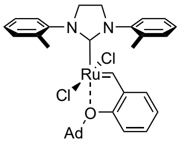	HIGJEP	1	1	119.126	119.922
8[b]	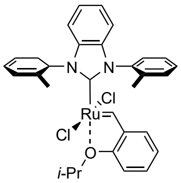	MOWREX	1	1	122.872	125.201
9[b]	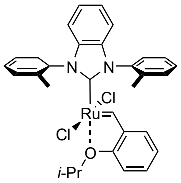	MOWREX01	1	1	122.206	123.005
10	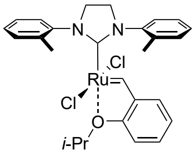 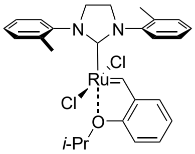	PIBXOO	1	1	119.527	119.8

11	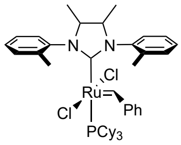	QUDDAV	1	1	120.144	122.066
12	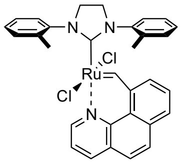	RACTAT	1	1	120.395	119.044
13	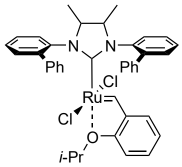	UXUGOL	1	1	119.076	120.765
14	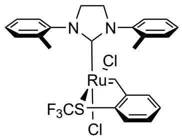	WEPHIL	1	1	123.126	120.349
15	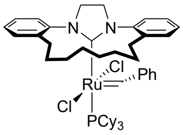 *trans*-**Ru6**	*trans*-**Ru6**			114.1(2)	118.5(2)
16	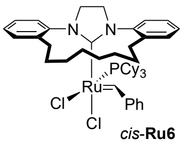 *cis*-**Ru6**	*cis*-**Ru6**			116.7(1)	119.9(2)
17	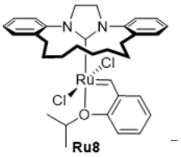 **Ru8**	**Ru8**			118.8(3)	119.5(3)

[a] HIGGIQ and HIGJEP are psudopolymorphs. [b] Same structure measured two times

S8. Dreiding Models of NHC ligands bearing chains of different length

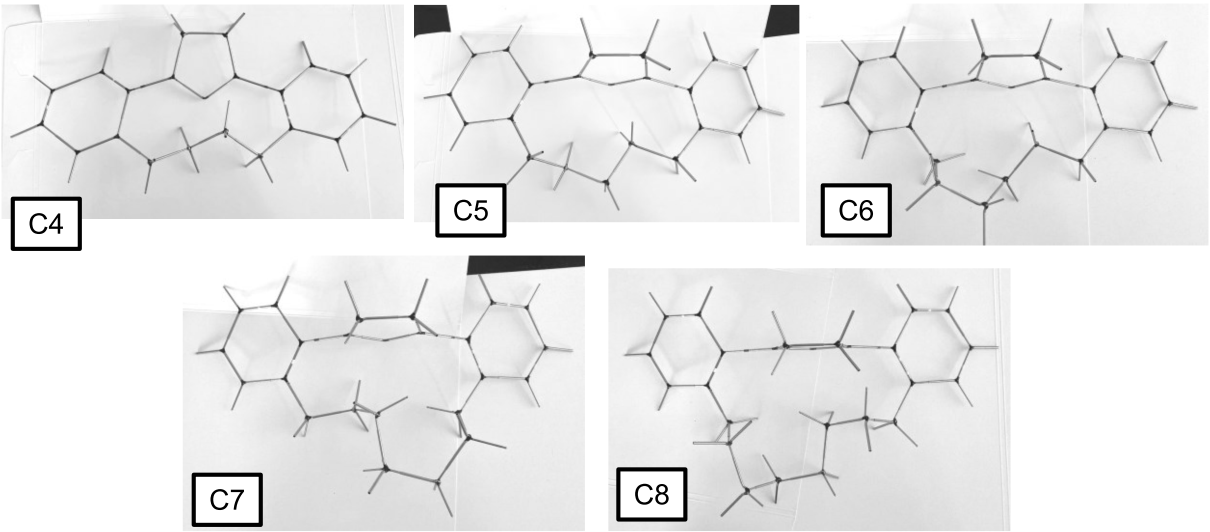

Figure S6. Dreiding models[10] of NHC ligands with chains of different length (from 4 to 8 -CH_2- groups).

[10] K. Wirth, A. S. Dreiding *J. Math. Chem.* **2014**, *52*, 1624-1638

S9. Computational studies

S9.1 Computational details

In this work, we have used a computational protocol similar to our previous computational investigations, which was previously shown to be a good choice for studying ruthenium catalysts of olefin metathesis.[11,12,13] Starting models for the precatalyst were prepared based on the crystal structure of a tolyl analogue of the studied complex.[14] All structures were optimised using the B3LYP density functional using the 6-31G* basis set for all atoms except the Ru atom, which was described by the Los Alamos angular momentum projected effective core potential (ECP) using the double-ζ contraction of valence functions (denoted as LACVP*). We have used the standard energy convergence criterion of $5x10^{-5}$ Hartree and the Jaguar v.9.5 software,[15] and for each structure, frequencies were calculated to verify the nature of each stationary point. The final energies were calculated with the M06-D3 single-point calculations with the 6-311++G** basis set for all atoms except Ru, which was described using the same ECP.[16] The Gibbs free energies discussed in this work for stationary points were calculated as the sum of electronic energy (single-point, using the M06-D3 functional), zero-point energy correction, thermal correction to enthalpy, negative product of temperature and entropy at 298 K and solvation energy calculated using the Poisson Boltzmann finite element method continuum solvation model with standard parameters for toluene, DCM, methanol and water.[17]

---

11 A. Zieliński, G. Szczepaniak, R. Gajda, K. Woźniak, B. Trzaskowski, D. Vidović, A. Kajetanowicz, K. Grela, *Eur. J. Inorg. Chem.* **2018**, *2018*, 3675-3685.
12 P. Małecki, K. Gajda, R. Gajda, K. Woźniak, B. Trzaskowski, A. Kajetanowicz, K. Grela, *ACS Catal.* **2019**, *9*, 587-598.
13 K. Grudzień, B. Trzaskowski, M. Smoleń, R. Gajda, K. Woźniak, K. Grela, *Dalton Trans.* **2017**, *46*, 11790-11799.
14 S. Planer, P. Małecki, B. Trzaskowski, A. Kajetanowicz, K. Grela, *ACS Catal.* **2020**, *10*, 11394-11404.
15 A. D. Bochevarov, E. Harder, T. F. Hughes, J. R. Greenwood, D. A. Braden, D. M. Philipp, D. Rinaldo, M. D. Halls, J. Zhang, R. A. Friesner, *Int. J. Quantum Chem.* **2013**, *113*, 2110-2142.
[16] Y. Zhao, D. G. Truhlar, *Theor. Chem. Acc.* **2008**, *120*, 215-241.
[17] D. J. Tannor, B. Marten, R. Murphy, R. A. Friesner, D. Sitkoff, A. Nicholls, B. Honig, M. Ringnalda, W. A. Goddard, *J. Am. Chem. Soc.* **1994**, *116*, 11875-11882.

S9.2 Computational results.

To further study the problem of *cis*-*trans* isomerization, we also considered in the computational part the standard 2nd generation Grubbs catalyst with mesityl side groups and its symmetric derivatives bearing relatively small tolyl as well as relatively bulky Dipp (2,6-diisopropylphenyl) moieties.[8,18,19,20] In all cases the solvation energy in all solvents favors the *cis* isomer, however, to a different extent. In the case of toluene the average *cis*-*trans* solvation energy difference of the studied complexes is 2.9 kcal/mol while for more polar solvents it is equal to 6.1 kcal/mol, 7.7 kcal/mol and 8.3 kcal/mol for DCM, methanol, and water, respectively. Since the no-solvent Gibbs free energy differences between *trans* and *cis* isomers are relatively large for mesityl (16.0 kcal/mol) and Dipp-substituted catalysts (19.3 kcal/mol), these solvation energy differences have no impact on the final preference for the *trans* isomer. In the case of tolyl-substituted complex the gas-phase difference between *trans* and *cis* isomers is only 8.3 kcal/mol, but it is also enough to make the *trans* isomer preferred in all solvents studied. On the other hand, the gas phase *trans*/*cis* difference of Ru6 is only 5.3 kcal/mol, suggesting a higher stability of the *trans* form in toluene and DCM, but a lower stability of this isomer in methanol and water.

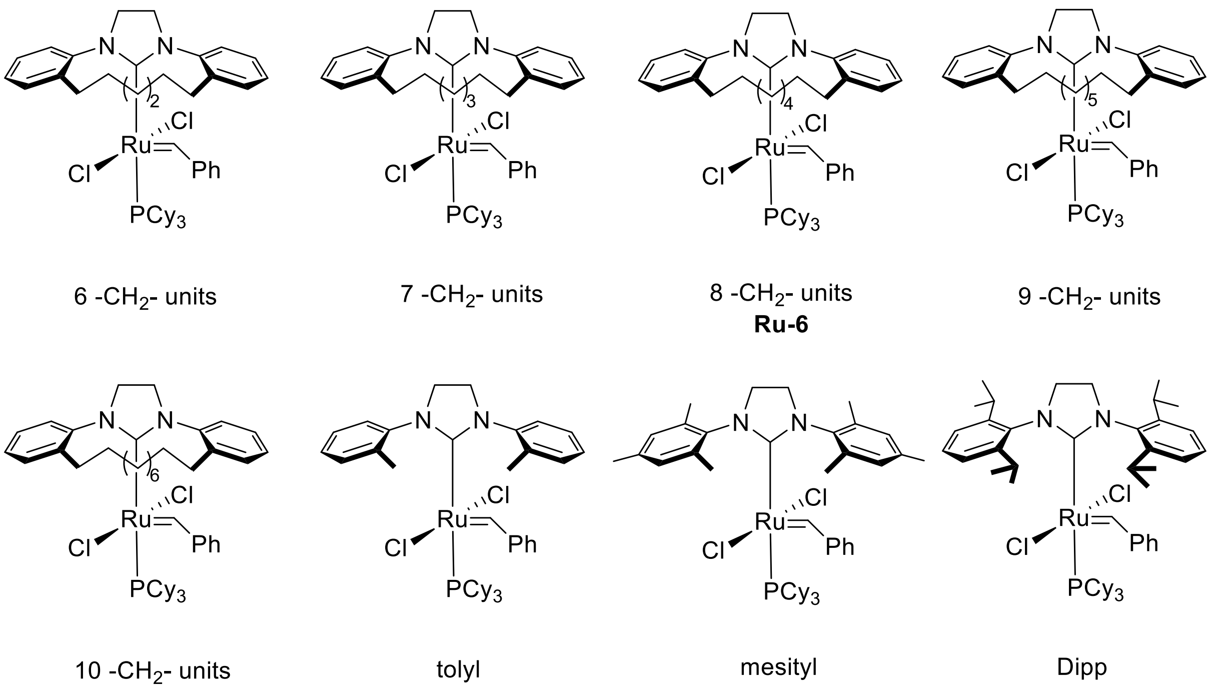

Figure S7. Structures of the ruthenium complexes studied computationally in this work.

[18] M. Scholl, T. M. Trnka, J. P. Morgan, R. H. Grubbs, *Tetrahedron Lett.* **1999**, *40*, 2247-2250.
[19] J. Huang, E. D. Stevens, S. P. Nolan, J. L. Petersen, *J. Am. Chem. Soc.* **1999**, *121*, 2674-2678.
[20] A. Fürstner, L. Ackermann, B. Gabor, R. Goddard, C. W. Lehmann, R. Mynott, F. Stelzer, O. R. Thiel, *Chem. Eur. J.* **2001**, *7*, 3236-3253.

Table S4. Estimated Gibbs free energy differences between the *trans* and *cis* isomers of selected Grubbs-like complexes in different solvents.

**Complex**	**Gibbs free energy difference (kcal/mol)**				
	no solvent	toluene	DCM	methanol	water
6 –CH_2– units	-6.1	-3.2	0.6	2.3	2.9
7 –CH_2– units	-8.3	-5.5	-2.0	-0.3	0.6
8 –CH_2– units	-5.5	-2.7	0.6	2.2	2.9
9 –CH_2– units	-8.6	-5.9	-2.8	-1.1	-0.4
10 –CH_2– units	-5.0	-2.4	1.1	2.8	3.5
tolyl	-8.3	-5.4	-2.0	-0.3	0.3
mesityl	-16.0	-12.5	-9.6	-7.7	-7.5
Dipp	-19.3	-16.2	-13.4	-12.2	-11.9

Table S5. Estimated Gibbs free energy differences between the *trans* and *cis* isomers of selected Grubbs-like complexes with the PCy_3 part replaced by PH_3 in different solvents.

**Complex**	**Gibbs free energy difference (kcal/mol)**				
	no solvent	toluene	DCM	methanol	water
6 –CH_2– units	-2.6	0.0	2.8	4.3	4.9
7 –CH_2– units	-2.7	-0.5	2.6	3.9	4.7
8 –CH_2– units	-0.7	1.7	4.5	5.7	6.5
9 –CH_2– units	-0.9	1.5	4.1	5.4	5.9
10 –CH_2– units	-0.6	1.4	-4.5	5.8	6.4
tolyl	-2.7	0.1	2.9	3.8	4.7
mesityl	-1.1	1.5	4.2	5.1	5.8
Dipp	1.9	4.5	7.5	8.7	8.5

In the search for the transition state for the *cis*/*trans* isomerisation, we first explored the concerted mechanism, where the *trans* isomers converts to a *cis* isomer through a single transition state. In this transition state, similarly to the Berry pseudorotation mechanism, the phosphine moves closer to NHC and in between the two chlorine atoms. The Gibbs free energy barrier of this transition state is in the range of 25.6 – 25.8 kcal/mol, depending on the solvent used in the calculations and is much lower than the barrier of the one-step mechanism, in which the phosphine is moved close to the *trans* position with respect to one chlorine atom, while the second chlorine atom shifts to the *trans* position with respect to the Ru=C bond (with the barrier of more than 48 kcal/mol). As a result, the Berry-like concerted mechanism seems to be the only feasible one, and the barrier of this transition state are in very good agreement with the experimental data where we observe a very slow isomerisation to the *cis* isomer.

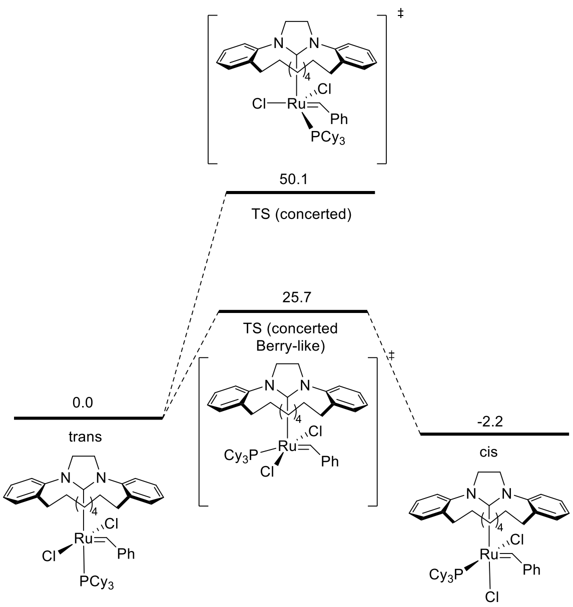

Scheme S3. Gibbs free energy profiles of the **Ru6** isomerisation in methanol.

S10. Reaction profiles for RCM of 1a

To provide insight into the differences in catalytic properties between cis/trans **Ru6** complexes, we determine the reaction profiles of catalyzed RCM process for DEDAM derivative (**1a**) using both *cis*-**Ru6** and *trans*-**Ru6** at rt with and without CuCl (Figure S8a).

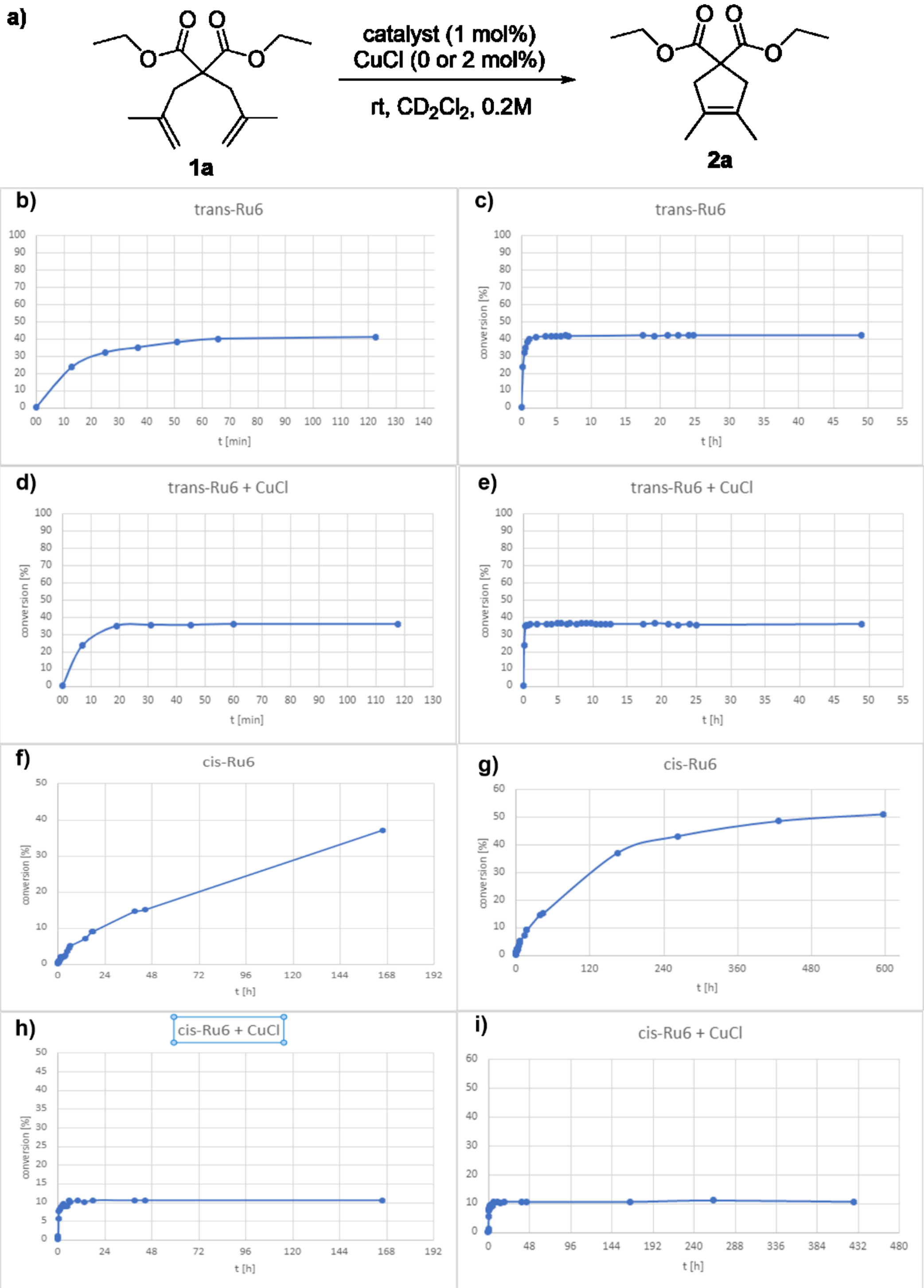

Figure S8. Reaction profiles of RCM reaction of **1a** (a) catalysed by **Ru6** catalysts. b) and c) *trans*-**Ru6** as a catalyst without CuCl. d) and e) *trans*-**Ru6** as a catalyst with 2 mol% of CuCl. f) and g) *cis*-**Ru6** as a catalyst without CuCl. h) and i) *cis*-**Ru6** as a catalyst with 2 mol% of CuCl.

Comment to the experiments described in Figure S8:

i) Reaction profile for RCM of **1a** catalyzed by *trans*-**Ru6** shows that after about 2h the reaction conversion reaches almost maximum level (41%). After this time the benzylidene signal which originates from *trans*-**Ru6** complex is not detectable (NMR) suggesting activation or decomposition of almost full amount of *trans*-**Ru6** used in this process. Further monitoring (24h) of reaction did not show any significant progress in term of conversion. This suggests that *trans*-**Ru6** can easily activate at rt to give 42% of conversion.

ii) Reaction profile for RCM of **1a** catalyzed by *trans*-**Ru6** in the presence of CuCl unrevealed that the reaction is completed in shorter (in comparison to analogous reaction without CuCl) period (after 20 min) reaching almost maximum conversion equals to 35%. After this time the benzylidene signal which originates from *trans*-**Ru6** complex is not detectable (NMR) suggesting activation or decomposition of almost full amount of *trans*-**Ru6** used in this process. Therefore faster activation of catalyst results small erosion of yield in comparison to analogous experiment without CuCl.

iii) Reaction profile for RCM of **1a** catalyzed by *cis*-**Ru6** shows that catalyst activate very slowly at rt reaching around 10% after 18h. Further monitoring of the reaction shows progress in the conversion reaching 51% after 25 days. Noteworthy, during the observation of reaction progress, the benzylidene signal which originates from *cis*-**Ru6** complex is detectable (NMR) suggesting that significant portion of *cis*-**Ru6** remained unactivated or not decomposed.

iii) Reaction profile for RCM of **1a** catalyzed by *cis*-**Ru6** in the presence of CuCl shows that the reaction is completed in shorter (in comparison to analogous reaction without CuCl) period (after 10h) reaching maximum conversion equals to 10%. After this time the benzylidene signal which originates from *cis*-**Ru6** complex is not detectable (NMR), suggesting activation or decomposition of almost full amount of *cis*-**Ru6** used in this process. Therefore forcing of faster activation of complex *cis*-**Ru6** by CuCl results in significant erosion of yield in comparison to the experiment with CuCl.

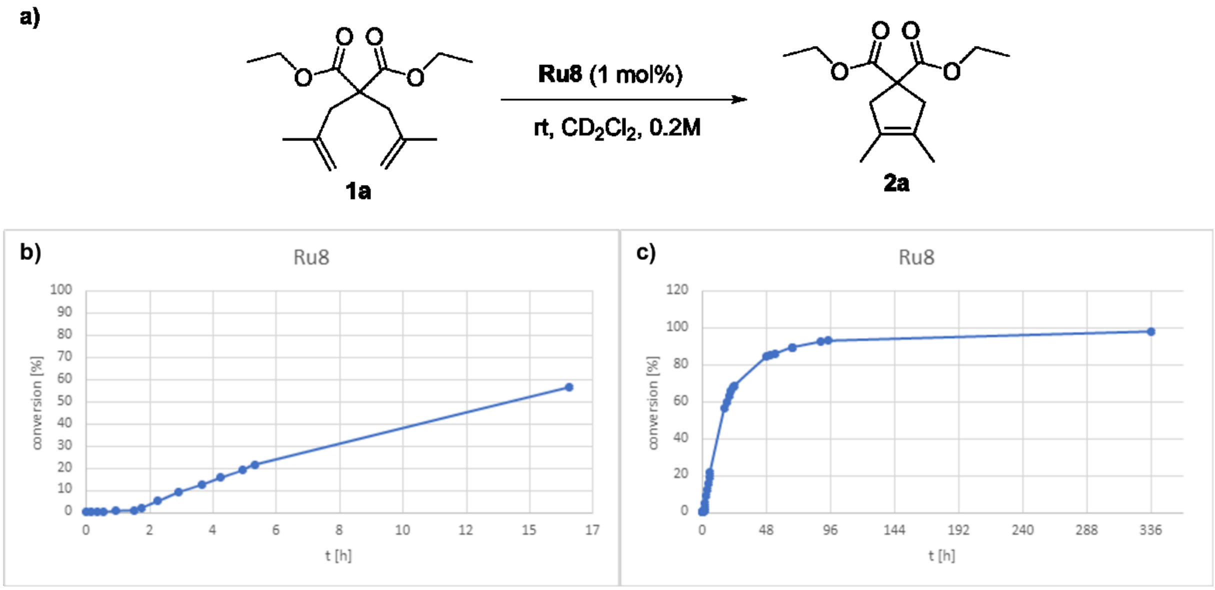

Figure S9. Reaction profiles of RCM reaction of **1a** (a) catalysed by **Ru8** catalyst at rt.

S11. Copy of NMR and MS spectra

1,8-bis(2-nitrophenyl)octane

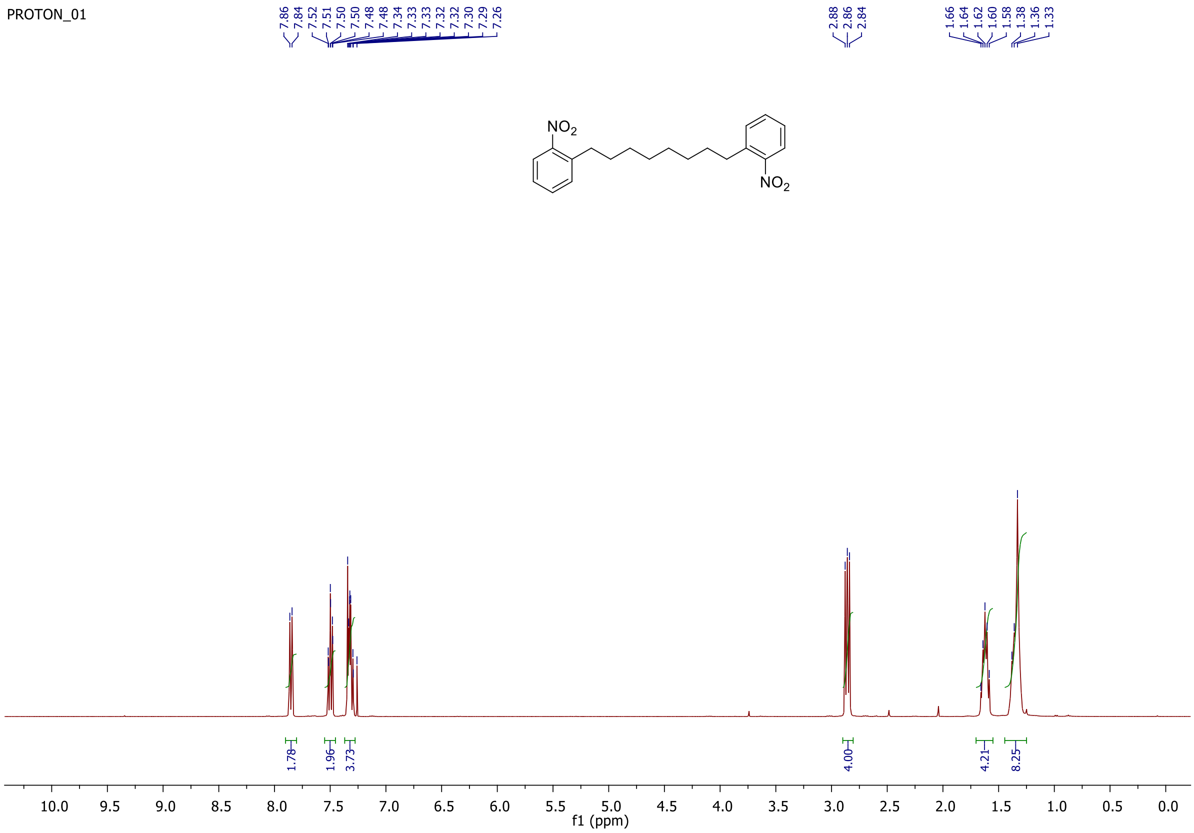

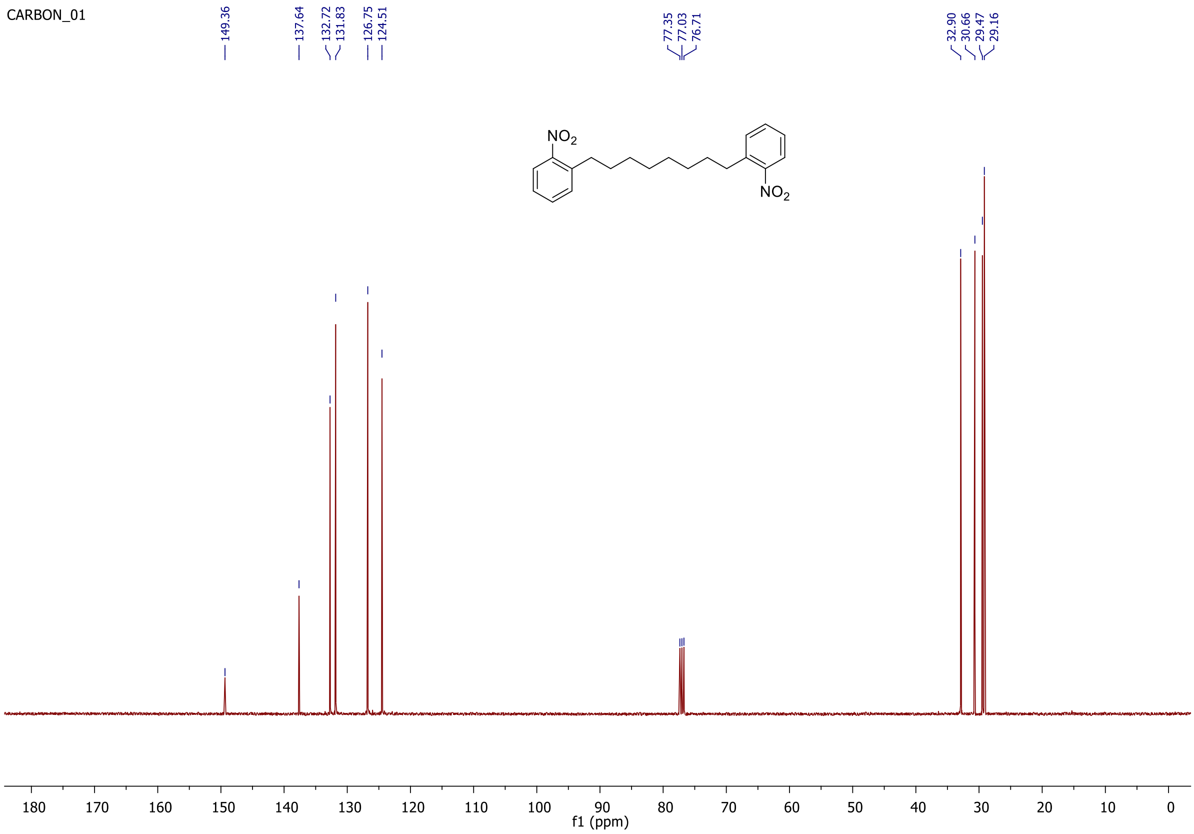
CARBON_01
149.36
137.64
132.72
131.83
126.75
124.51
77.35
77.03
76.71
32.90
30.66
29.47
29.16
NO_2
NO_2
180
170
160
150
140
130
120
110
100
90
80
70
60
50
40
30
20
10
0
f1 (ppm)

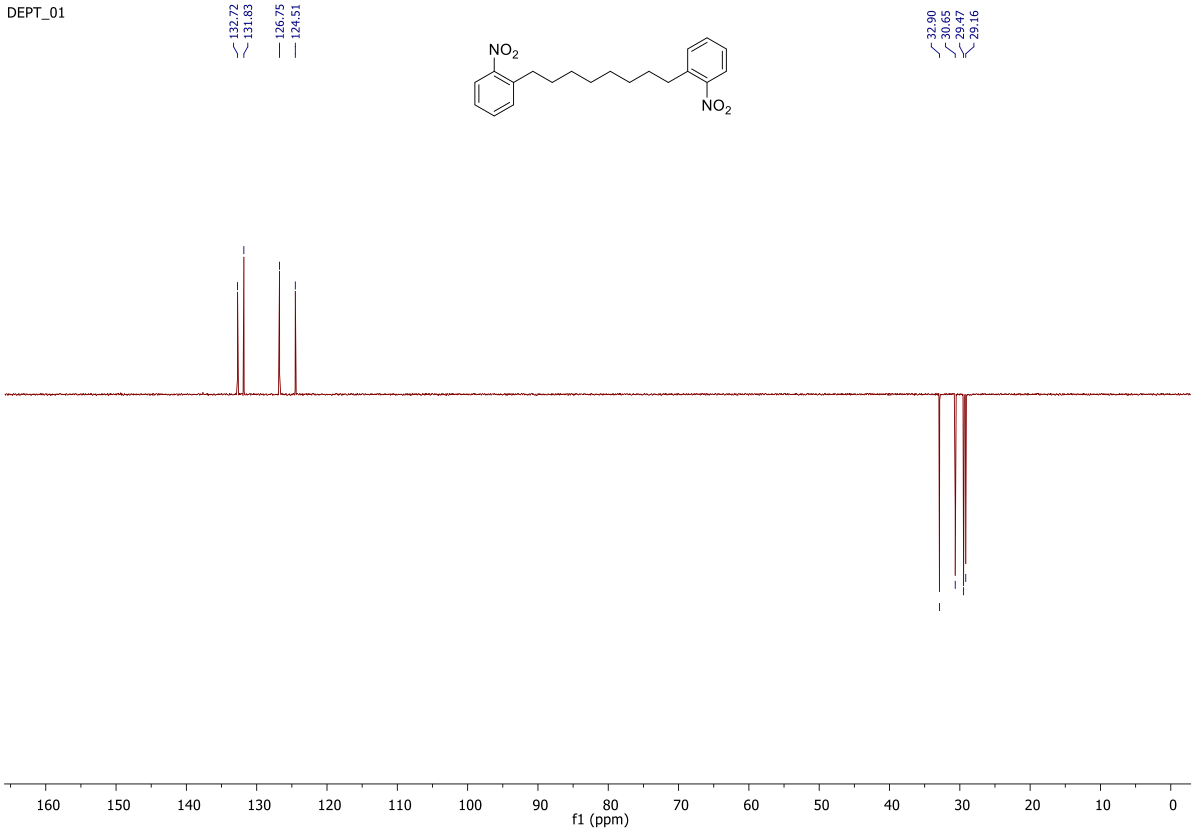
DEPT_01
132.72
131.83
126.75
124.51
32.90
30.65
29.47
29.16
NO_2
NO_2
160
150
140
130
120
110
100
90
80
70
60
50
40
30
20
10
0
f1 (ppm)

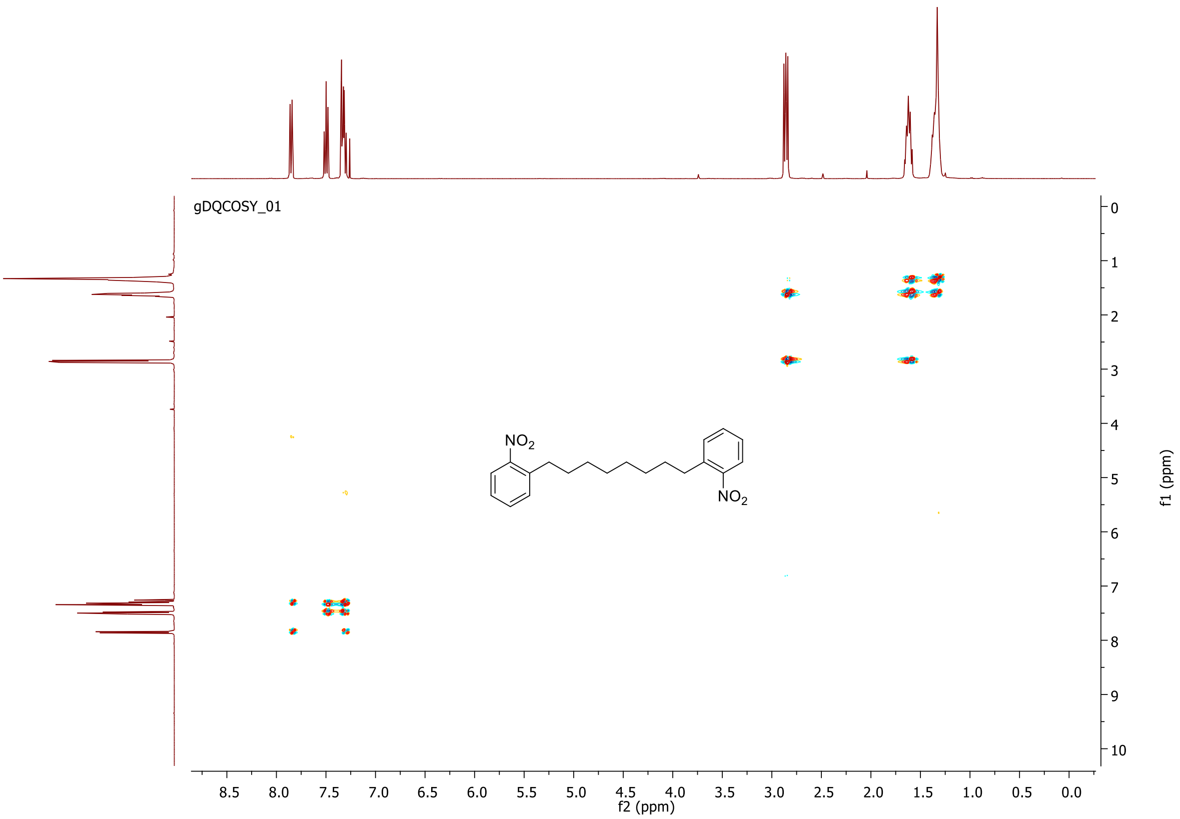
gDQCOSY_01
NO2
NO2
f1 (ppm)
f2 (ppm)

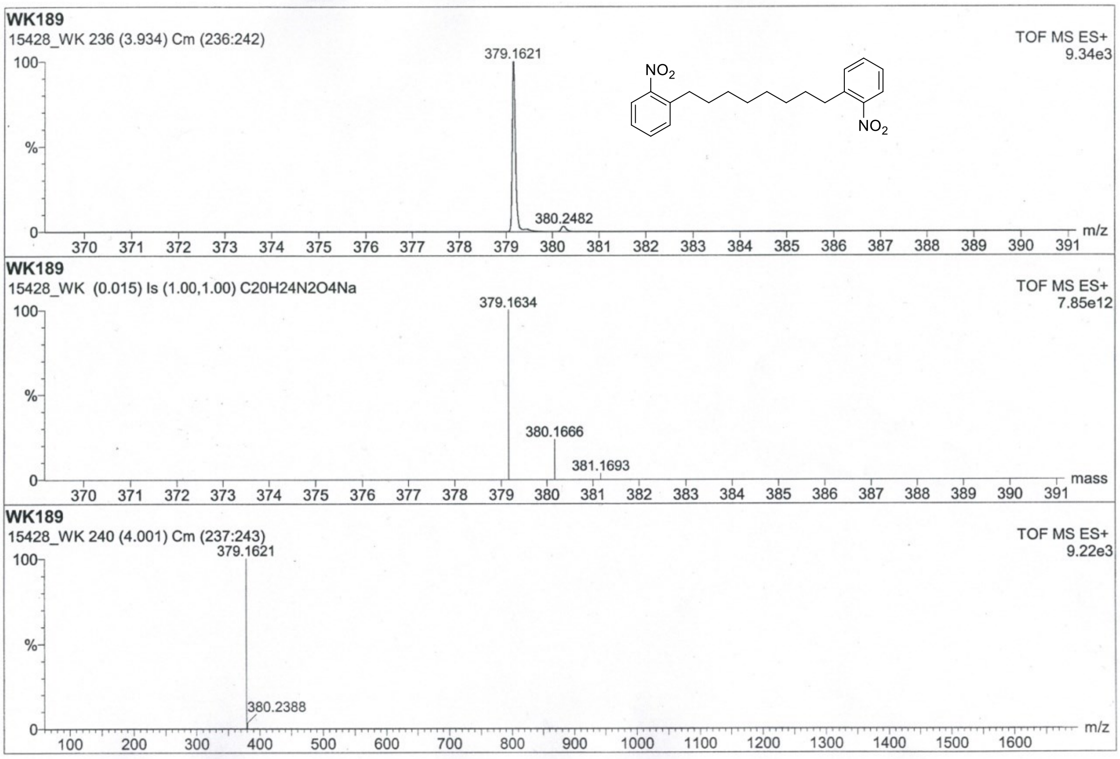
WK189
15428_WK 236 (3.934) Cm (236:242)
TOF MS ES+
9.34e3
379.1621
380.2482
m/z
WK189
15428_WK (0.015) Is (1.00,1.00) C20H24N2O4Na
TOF MS ES+
7.85e12
379.1634
380.1666
381.1693
mass
WK189
15428_WK 240 (4.001) Cm (237:243)
TOF MS ES+
9.22e3
379.1621
380.2388
m/z
NO2
NO2

Elemental Composition Report

Single Mass Analysis

Tolerance = 5.0 PPM / DBE: min = -0.5, max = 50.0

Monoisotopic Mass, Odd and Even Electron Ions

58 formula(e) evaluated with 1 results within limits (up to 30 closest results for each mass)

Minimum:				-0.5	
Maximum:		200.0	5.0	50.0	
Mass	Calc. Mass	mDa	PPM	DBE	Formula
379.1621	379.1634	-1.3	-3.4	9.5	C20 H24 N2 O4 Na

Synthesis of 2,2'-(octane-1,8-diyl)dianiline (4)

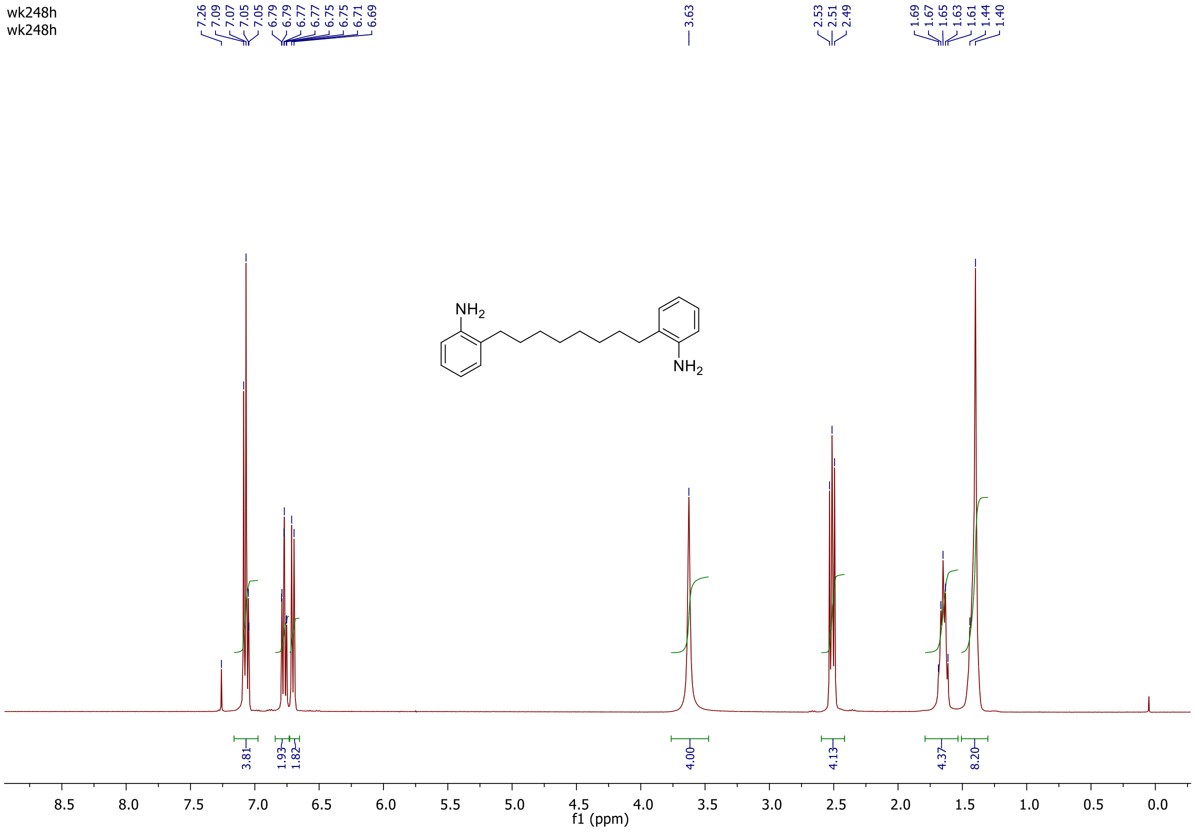

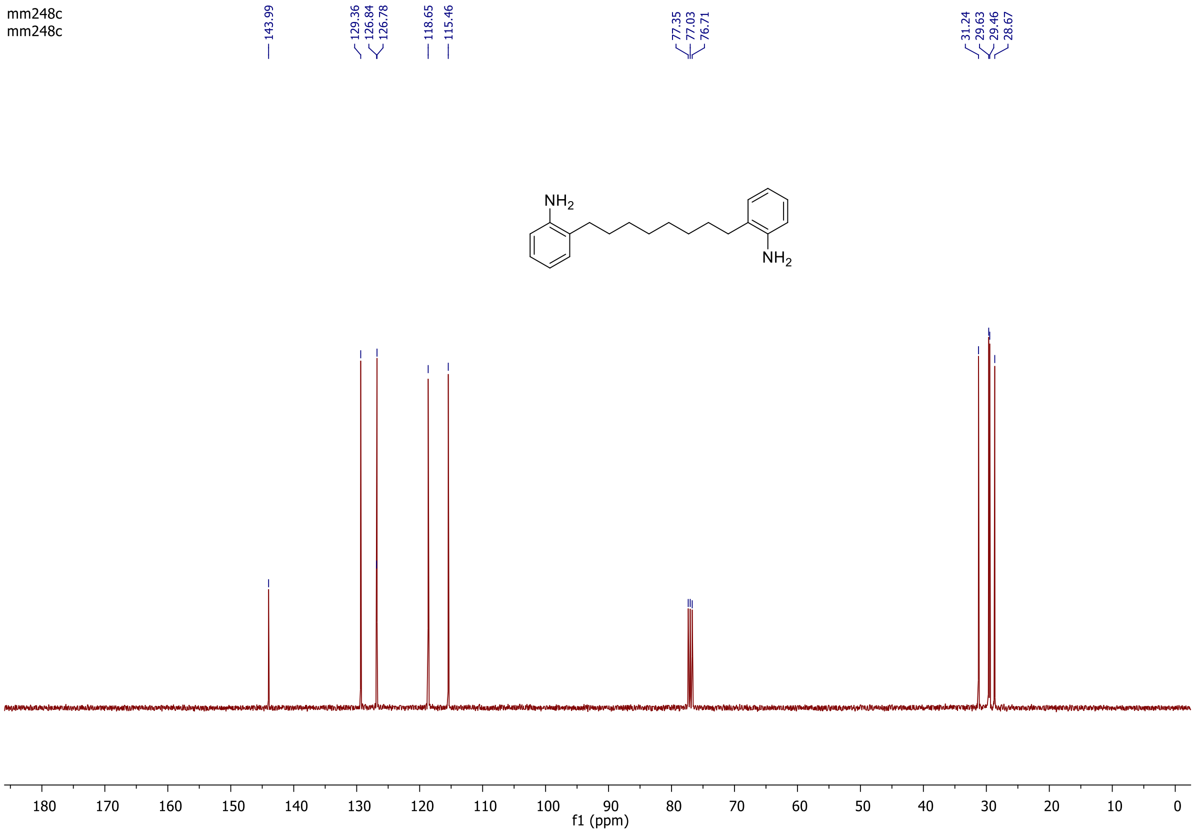

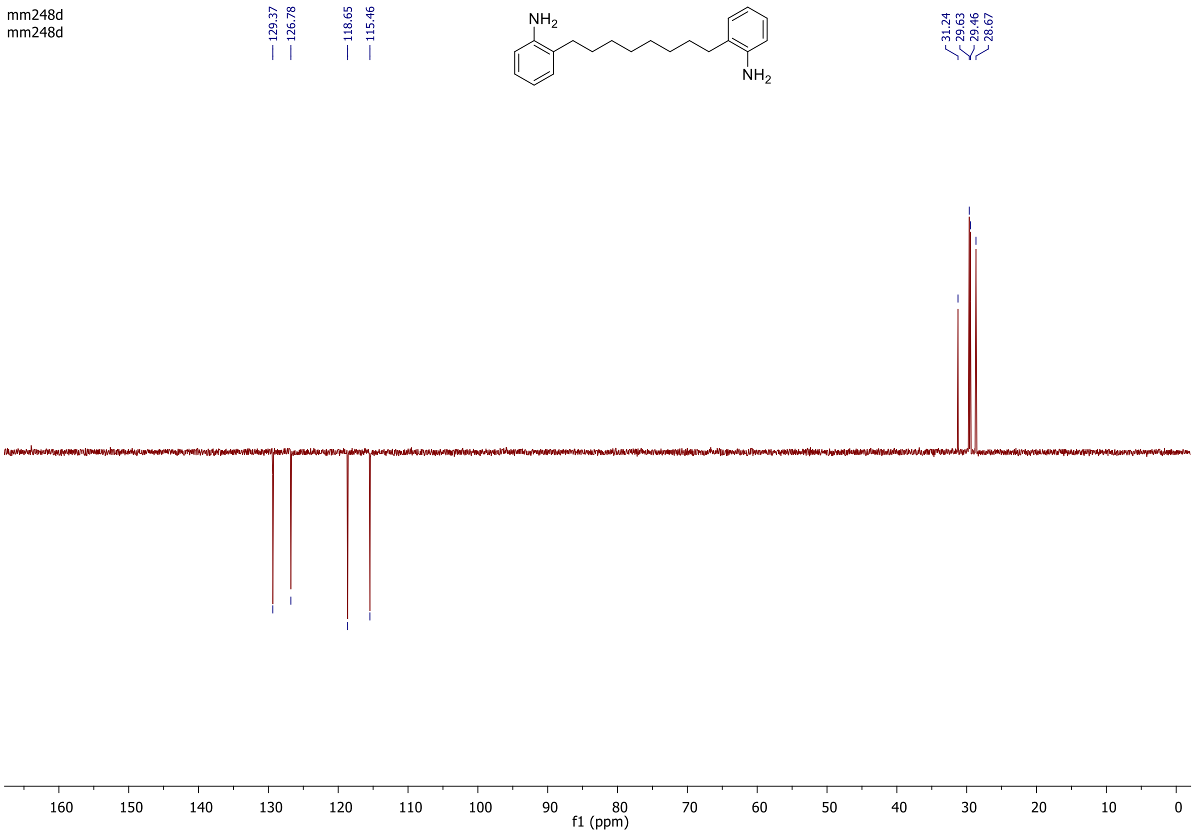

mm248d
mm248d
129.37
126.78
118.65
115.46
NH2
NH2
31.24
29.63
29.46
28.67
160 150 140 130 120 110 100 90 80 70 60 50 40 30 20 10 0
f1 (ppm)

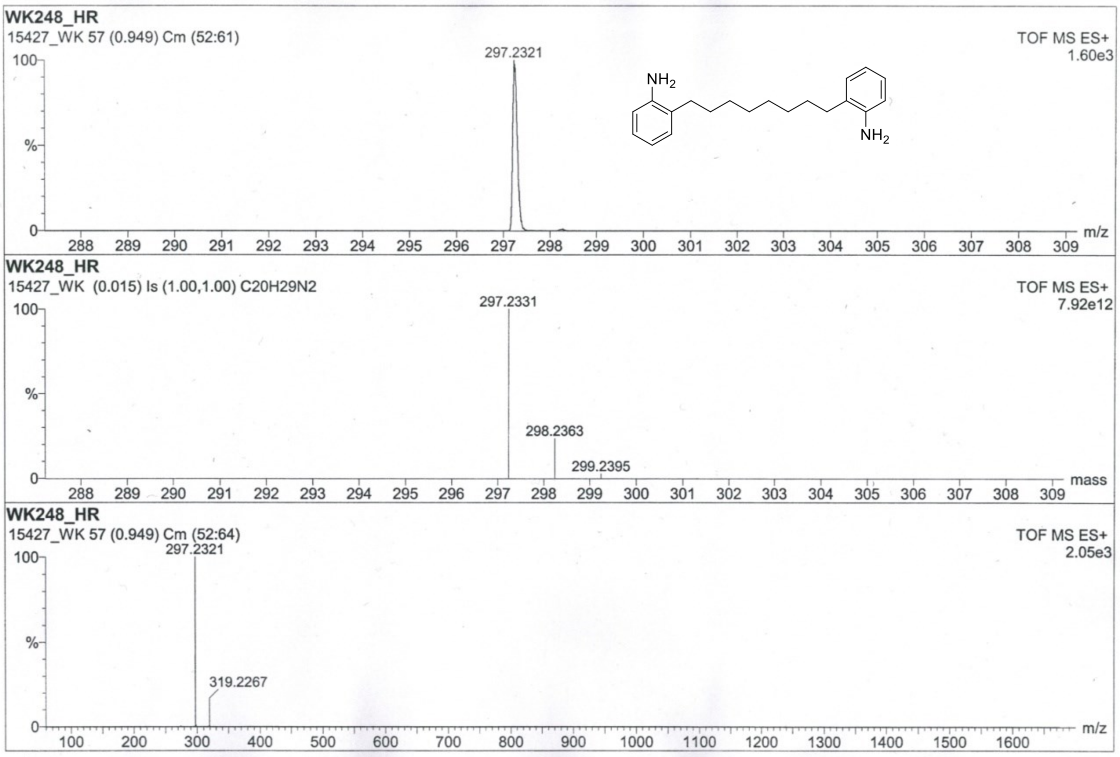

WK248_HR
15427_WK 57 (0.949) Cm (52:61)
TOF MS ES+
1.60e3
297.2321
NH2
NH2
m/z
WK248_HR
15427_WK (0.015) Is (1.00,1.00) C20H29N2
TOF MS ES+
7.92e12
297.2331
298.2363
299.2395
mass
WK248_HR
15427_WK 57 (0.949) Cm (52:64)
TOF MS ES+
2.05e3
297.2321
319.2267
m/z

Elemental Composition Report

Single Mass Analysis
Tolerance = 5.0 PPM / DBE: min = -0.5, max = 50.0

Monoisotopic Mass, Odd and Even Electron Ions
10 formula(e) evaluated with 1 results within limits (up to 30 closest results for each mass)

Minimum:				-0.5	
Maximum:		200.0	5.0	50.0	
Mass	Calc. Mass	mDa	PPM	DBE	Formula
297.2321	297.2331	-1.0	-3.4	7.5	C20 H29 N2

Macrocyclic diimine derivative

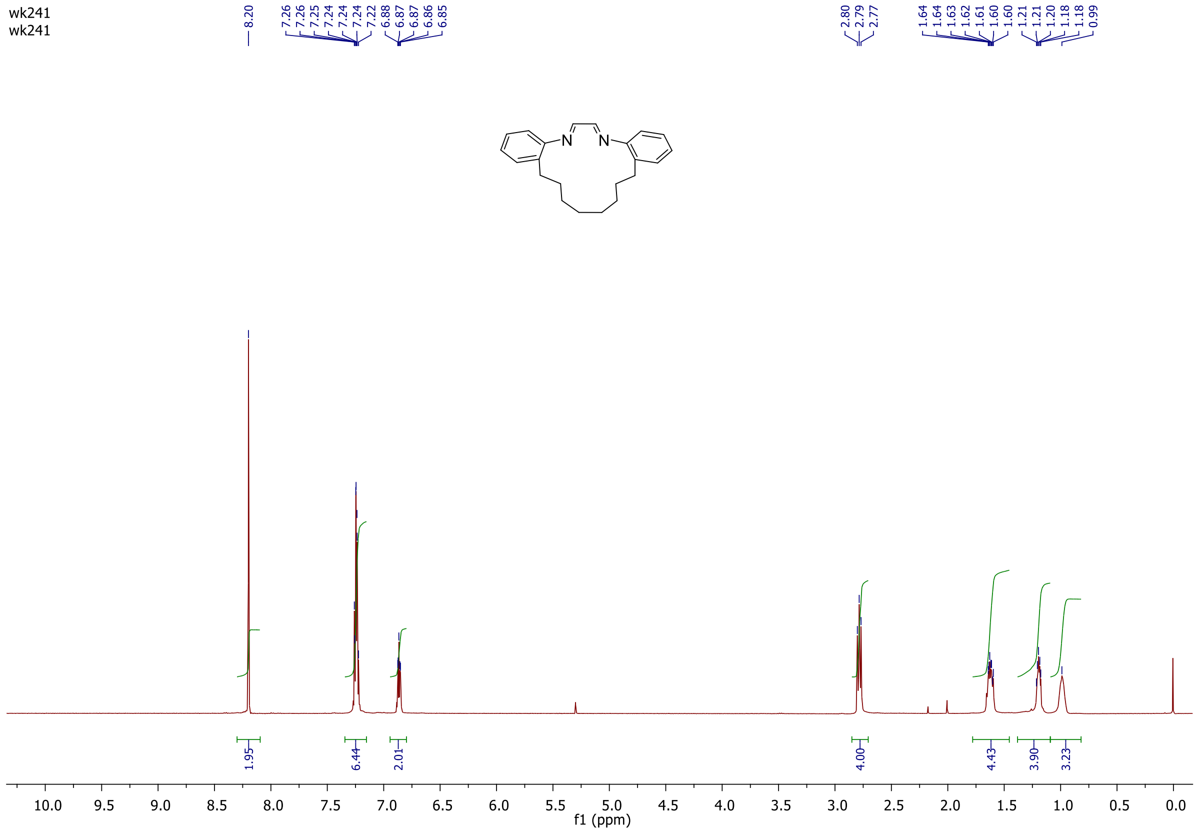

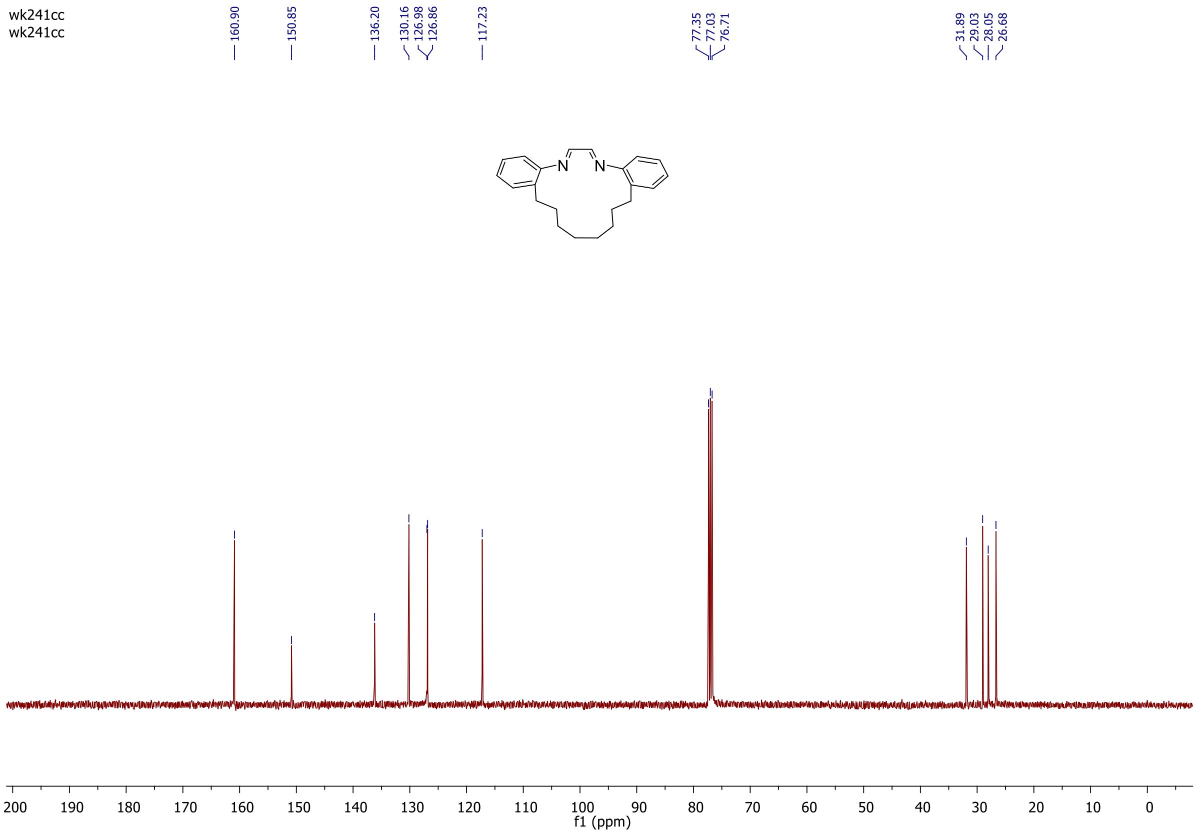

wk241cc
wk241cc
160.90
150.85
136.20
130.16
126.98
126.86
117.23
77.35
77.03
76.71
31.89
29.03
28.05
26.68
N
N
200
190
180
170
160
150
140
130
120
110
100
90
80
70
60
50
40
30
20
10
0
f1 (ppm)

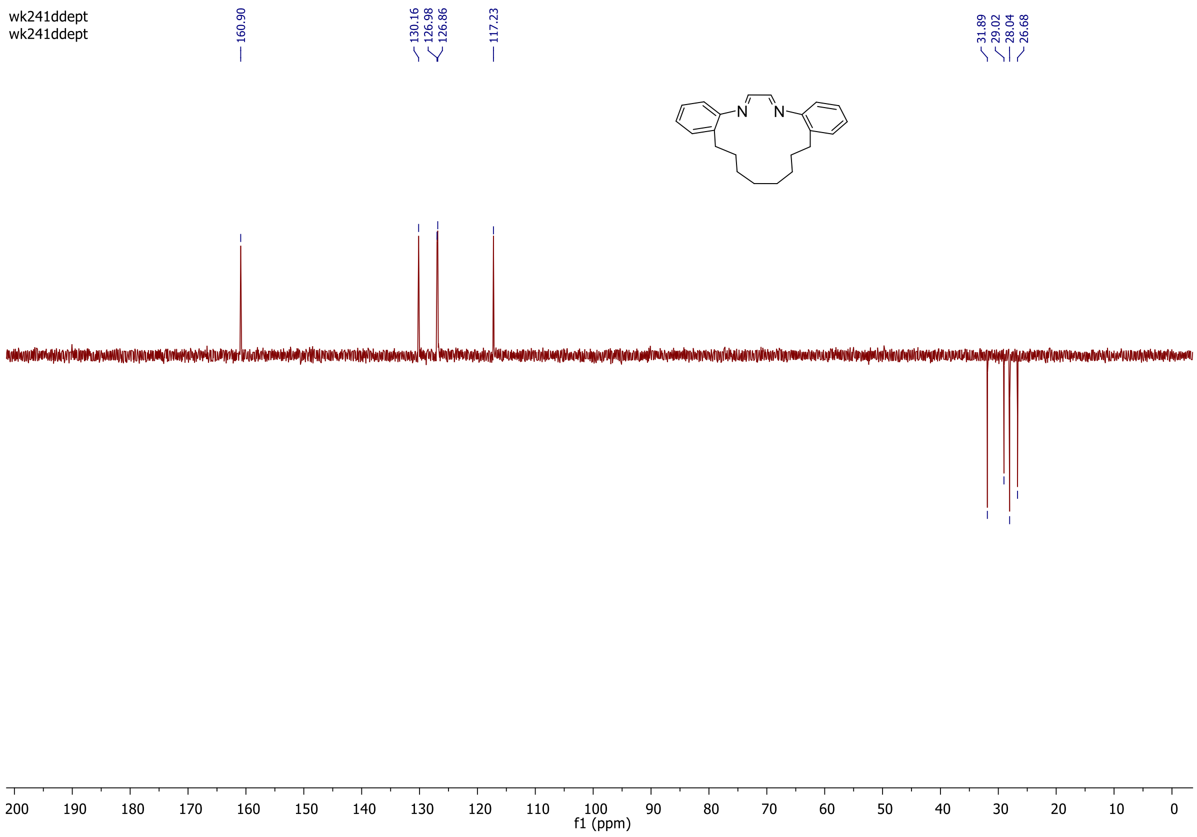

wk241ddept
wk241ddept
160.90
130.16
126.98
126.86
117.23
31.89
29.02
28.04
26.68
N
N
200
190
180
170
160
150
140
130
120
110
100
90
80
70
60
50
40
30
20
10
0
f1 (ppm)

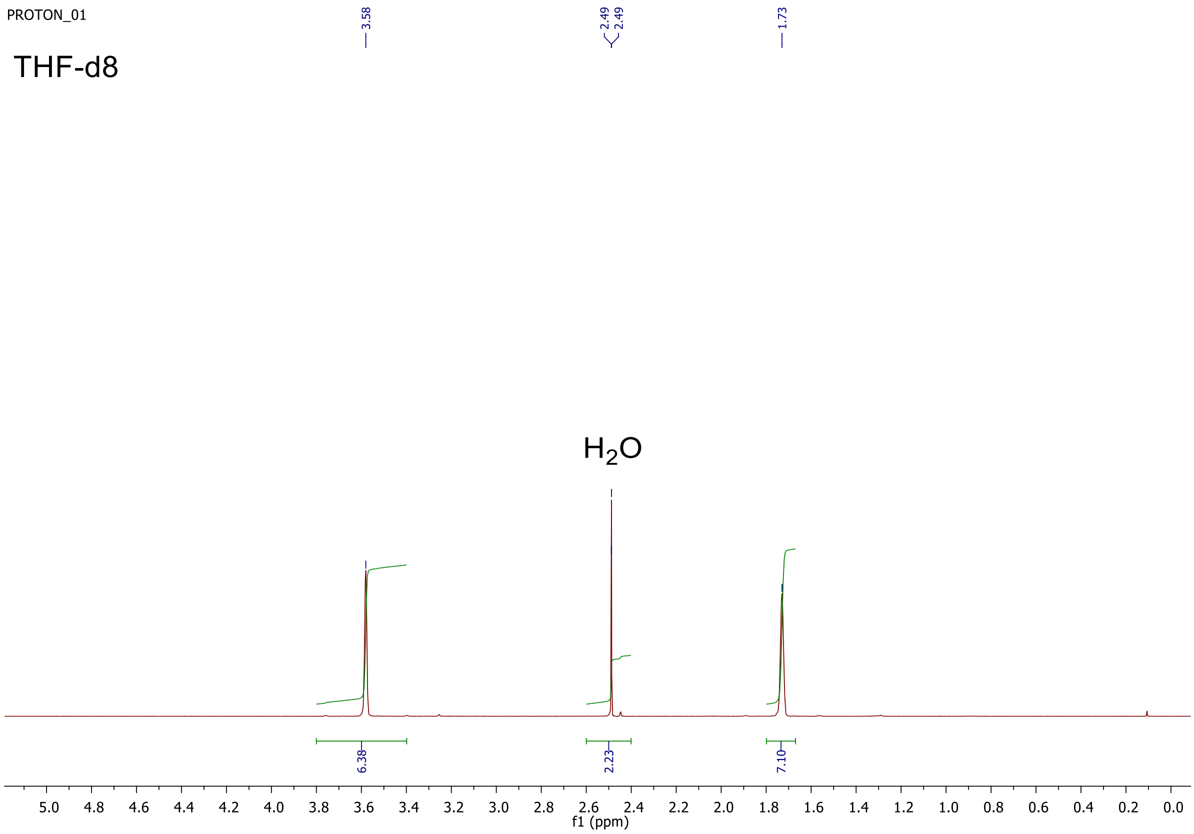
PROTON_01
THF-d8
H$_2$O
f1 (ppm)

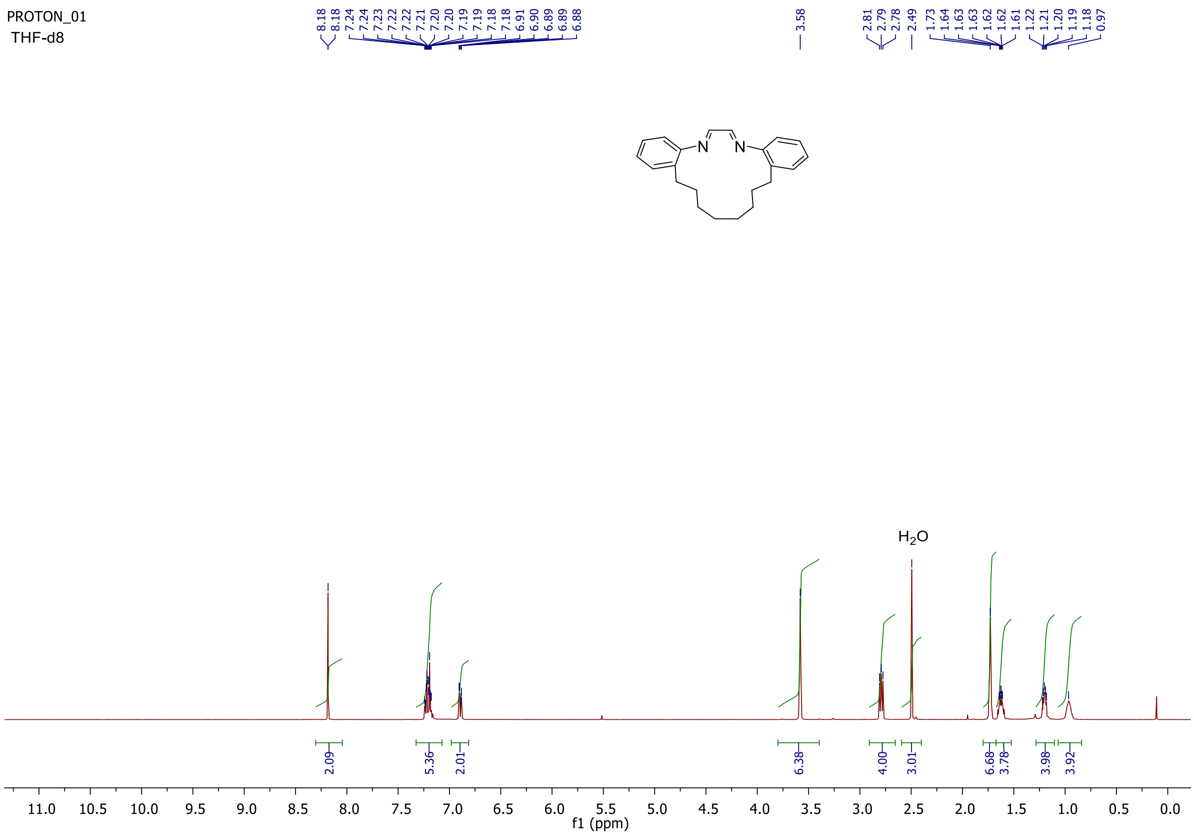
PROTON_01
THF-d8
N
N
H$_2$O
f1 (ppm)

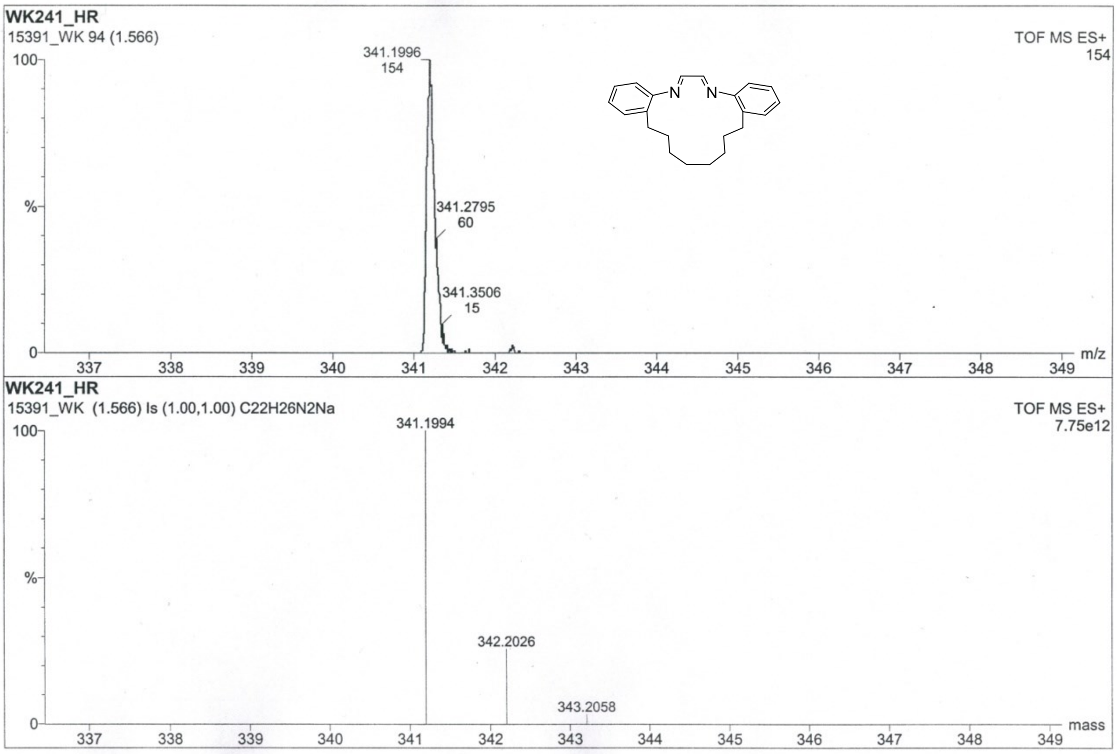

Elemental Composition Report

Page 1

Single Mass Analysis

Tolerance = 5.0 PPM / DBE: min = -0.5, max = 50.0

Monoisotopic Mass, Odd and Even Electron Ions
51 formula(e) evaluated with 1 results within limits (up to 30 closest results for each mass)

Minimum:				-0.5	
Maximum:		200.0	5.0	50.0	
Mass	Calc. Mass	mDa	PPM	DBE	Formula
341.1996	341.1994	0.2	0.7	10.5	C22 H26 N2 Na

Macrocyclic diamine derivative (5)

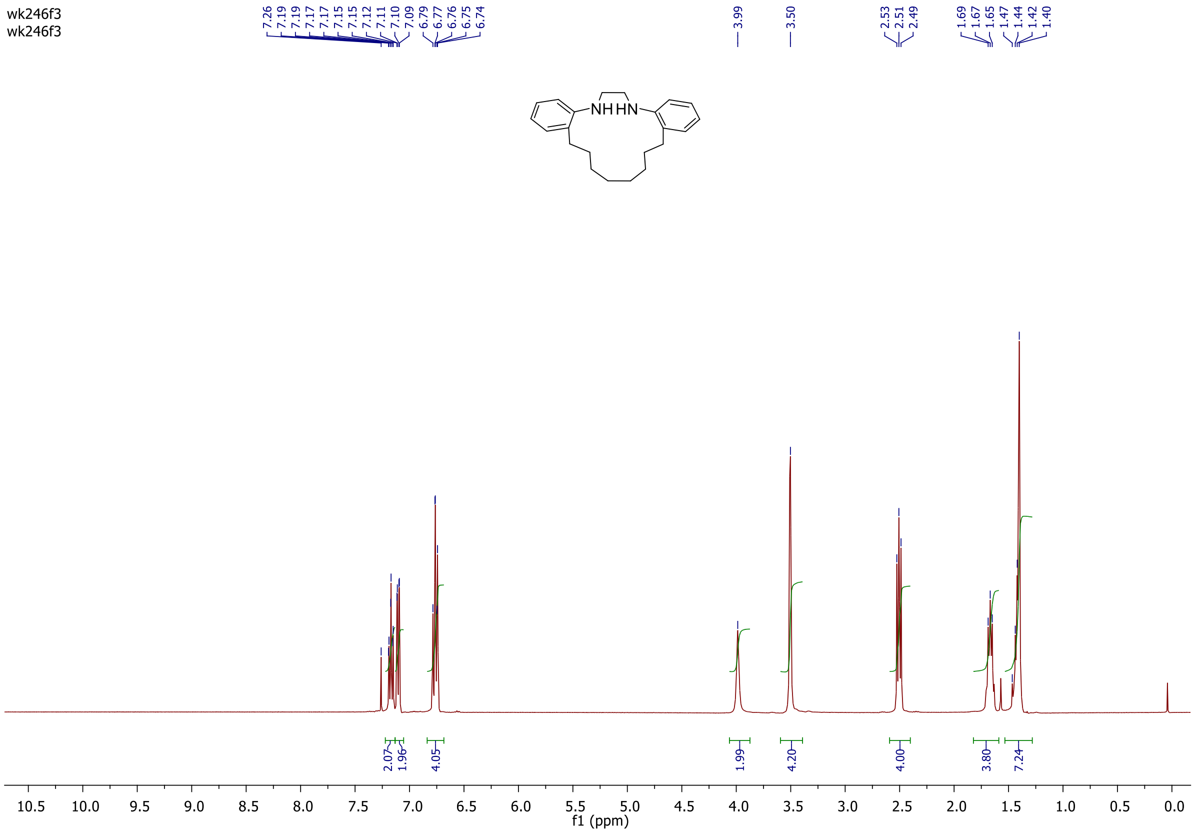

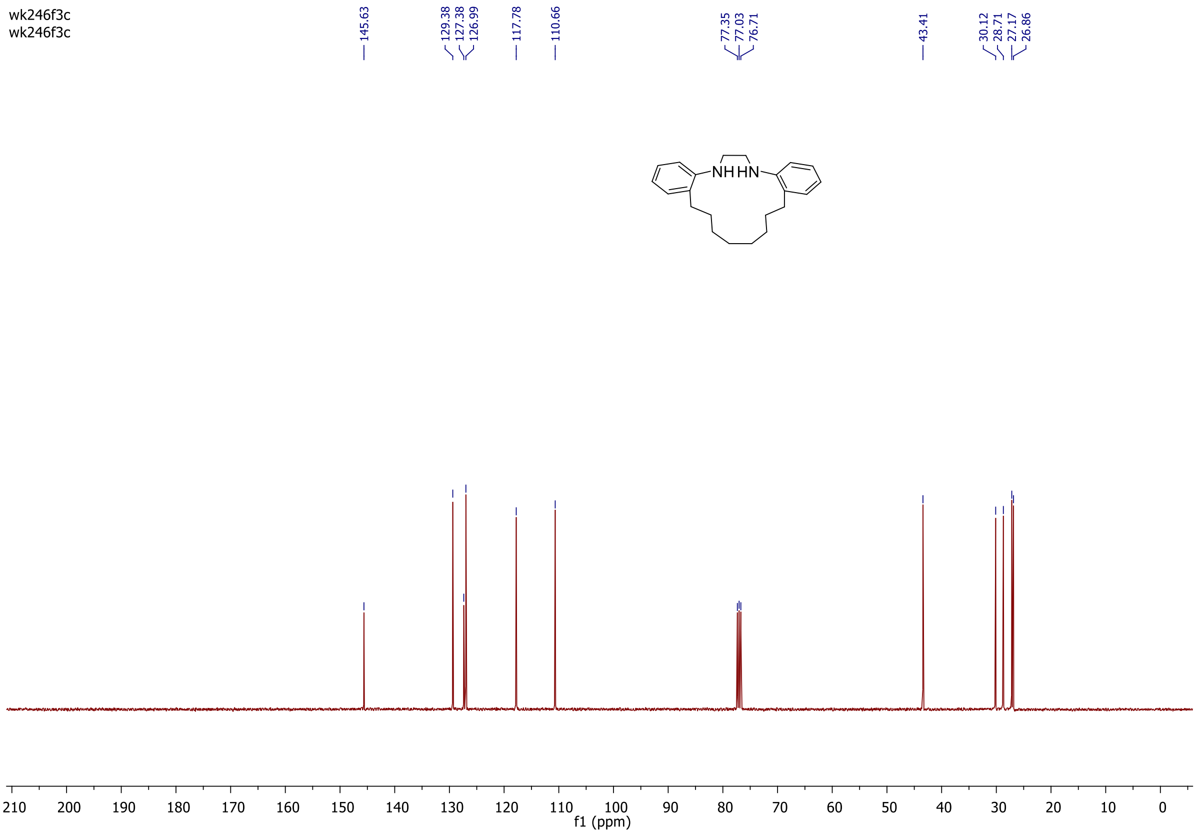

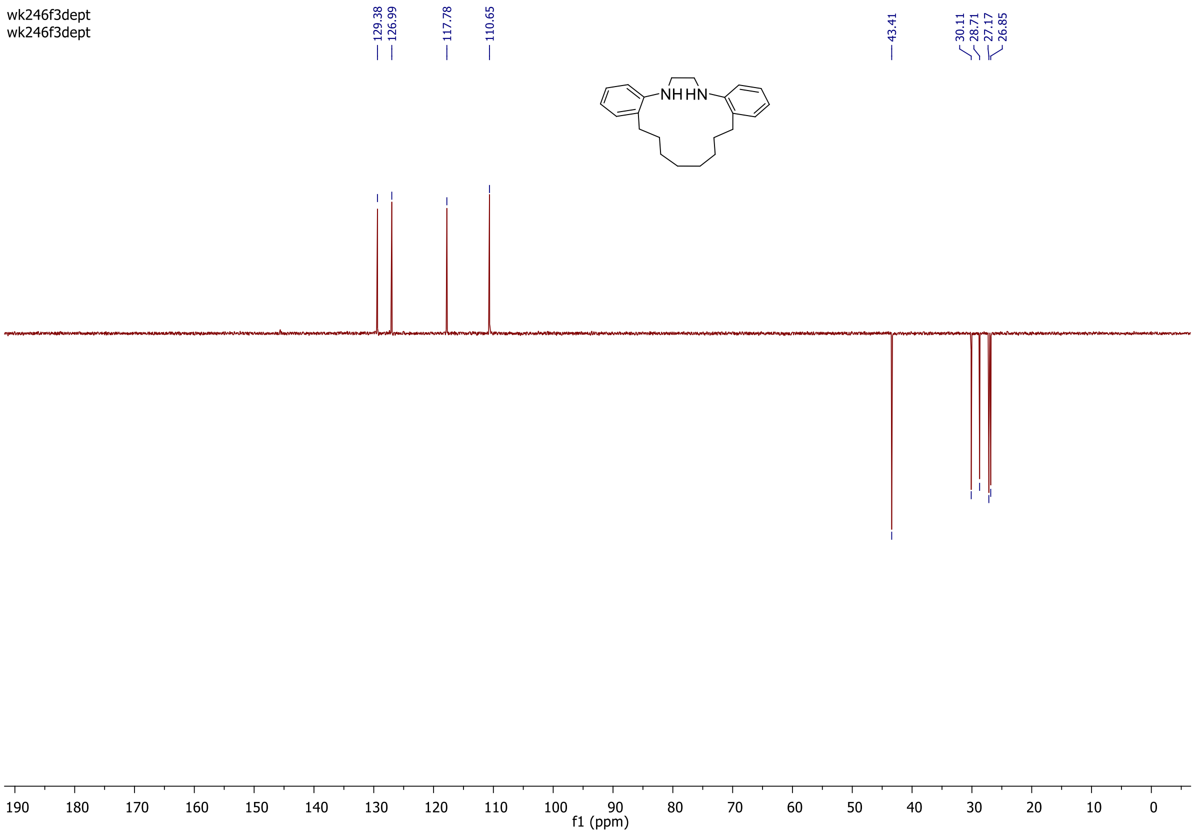
wk246f3dept
wk246f3dept
129.38
126.99
117.78
110.65
43.41
30.11
28.71
27.17
26.85
NH HN
190 180 170 160 150 140 130 120 110 100 90 80 70 60 50 40 30 20 10 0
f1 (ppm)

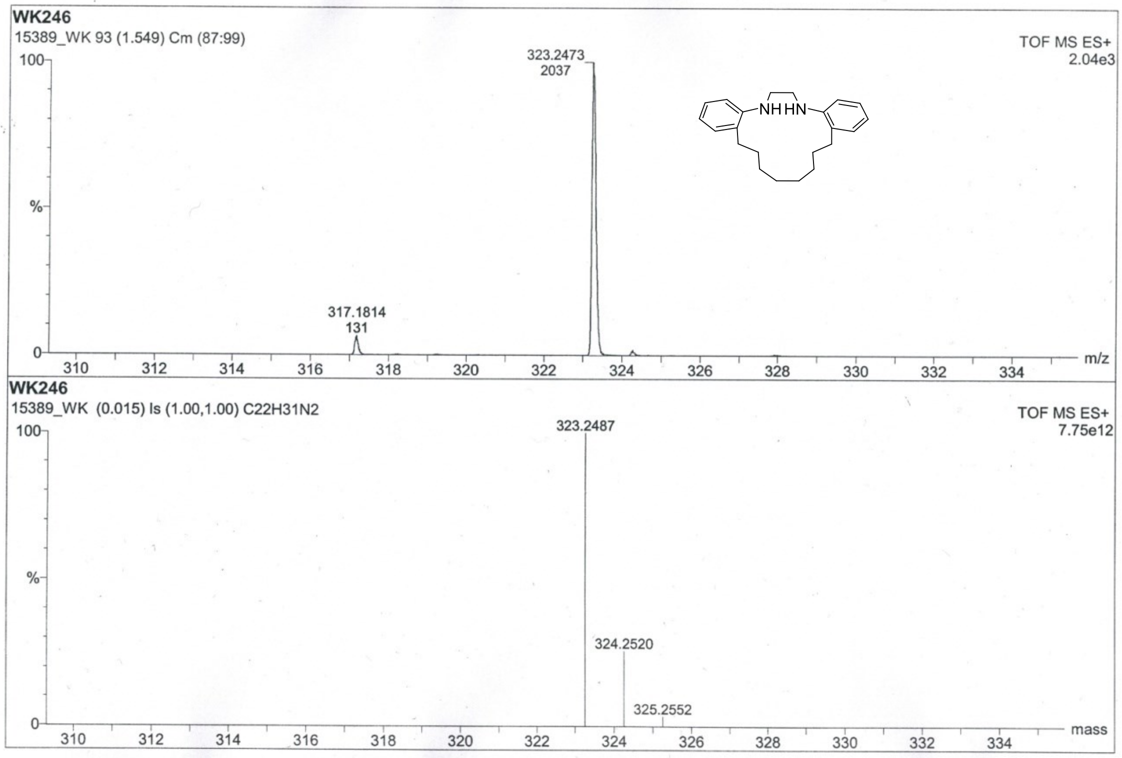
WK246
15389_WK 93 (1.549) Cm (87:99)
TOF MS ES+
2.04e3
323.2473
2037
317.1814
131
100
%
0
m/z
NH HN
WK246
15389_WK (0.015) Is (1.00,1.00) C22H31N2
TOF MS ES+
7.75e12
323.2487
324.2520
325.2552
mass

Elemental Composition Report

Page 1

Single Mass Analysis

Tolerance = 5.0 PPM / DBE: min = -0.5, max = 50.0

Monoisotopic Mass, Odd and Even Electron Ions
15 formula(e) evaluated with 1 results within limits (up to 30 closest results for each mass)

Minimum:				-0.5	
Maximum:		200.0	5.0	50.0	
Mass	Calc. Mass	mDa	PPM	DBE	Formula
323.2473	323.2487	-1.5	-4.6	8.5	C22 H31 N2

(*E*)-2^4,2^5-dihydro-2^1*H*-2(1,3)-imidazol-3-iuma-1,3(1,2)-dibenzenacycloundecaphan-2^3-ium chloride (6-Cl)

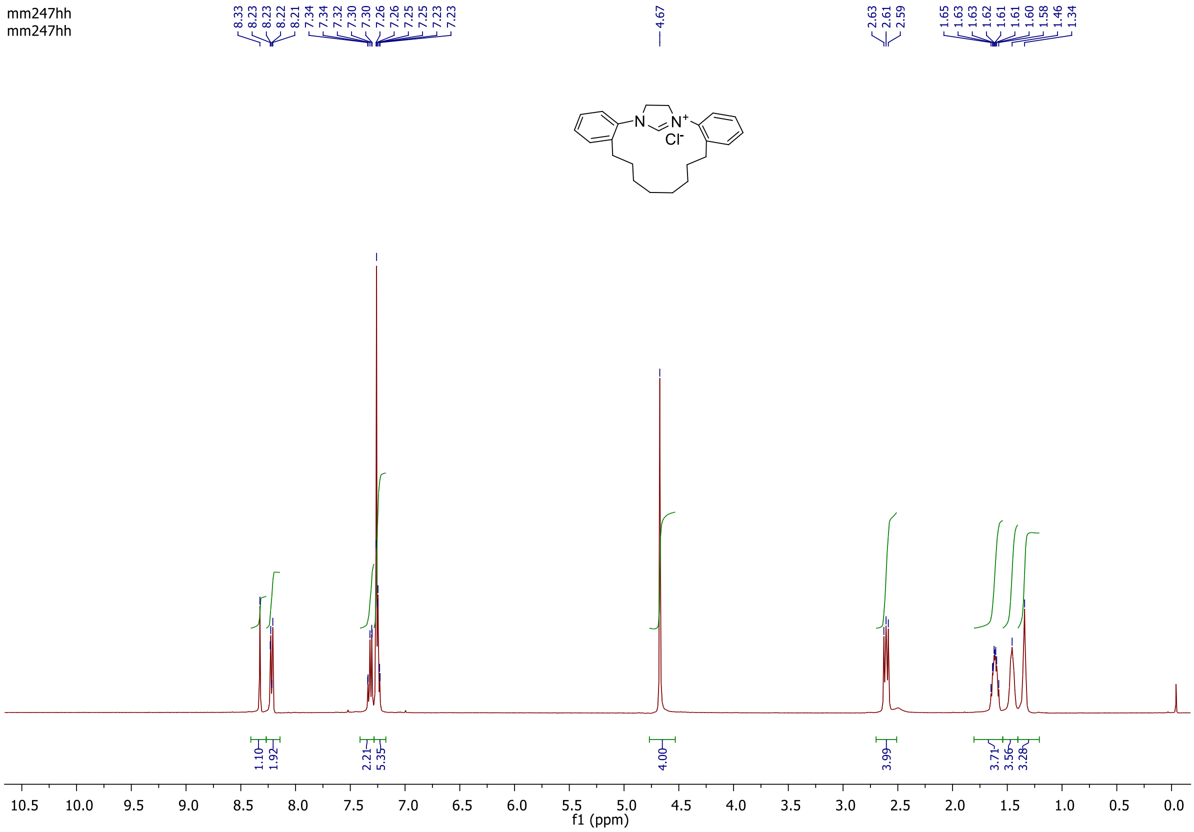

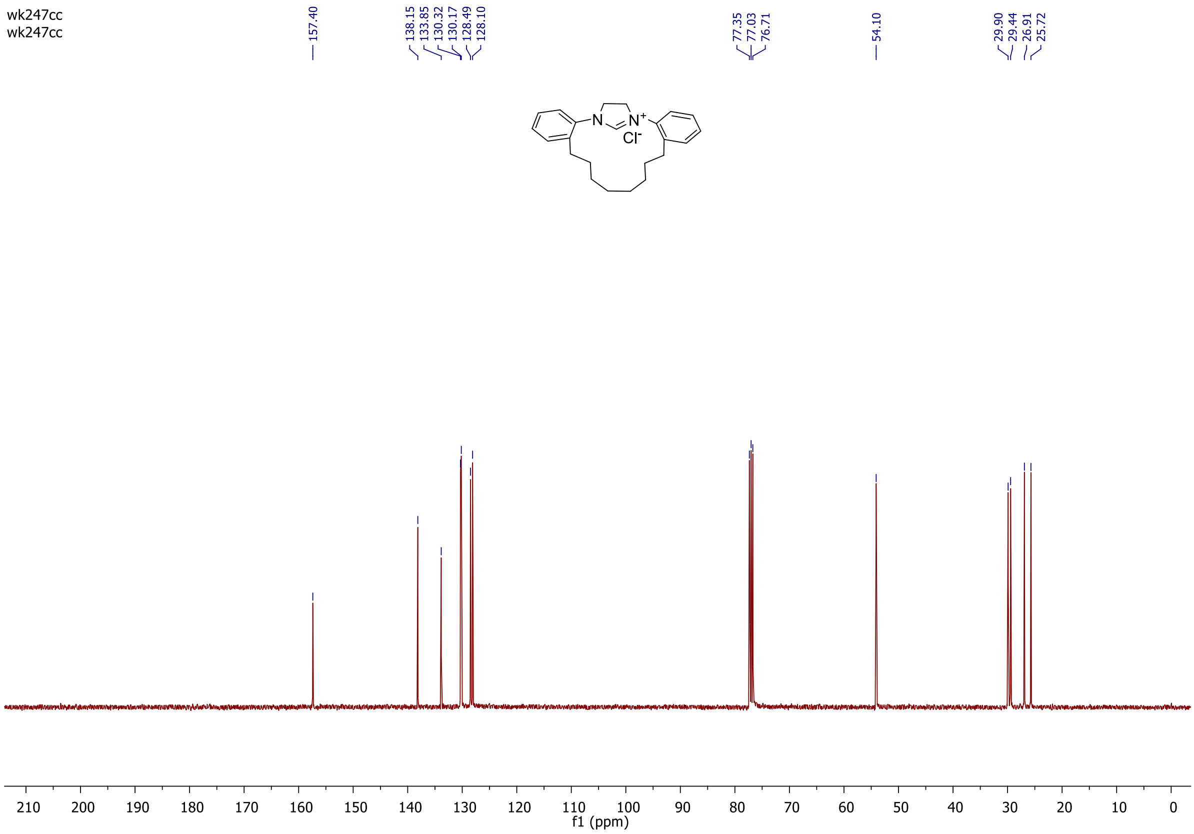

wk247cc
wk247cc
157.40
138.15
133.85
130.32
130.17
128.49
128.10
77.35
77.03
76.71
54.10
29.90
29.44
26.91
25.72
N
N+
Cl-
210 200 190 180 170 160 150 140 130 120 110 100 90 80 70 60 50 40 30 20 10 0
f1 (ppm)

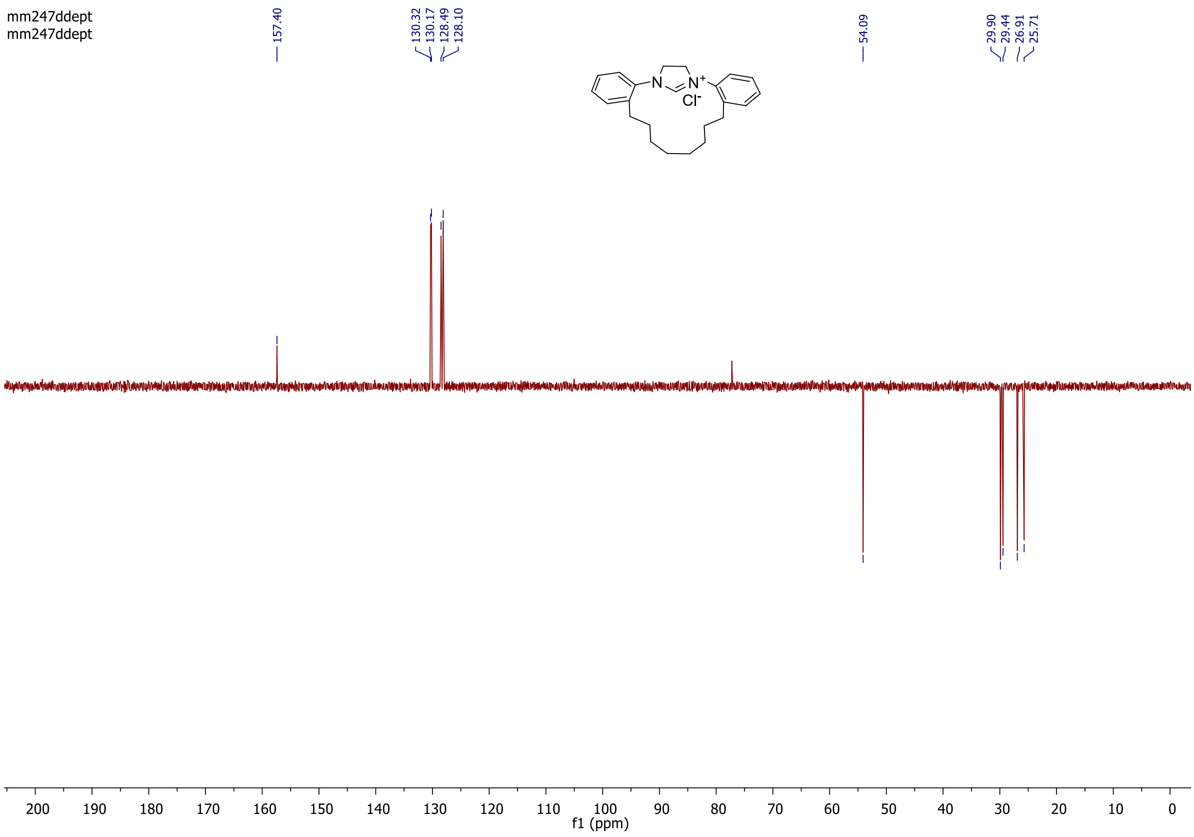

mm247ddept
mm247ddept
157.40
130.32
130.17
128.49
128.10
54.09
29.90
29.44
26.91
25.71
N
N+
Cl-
200 190 180 170 160 150 140 130 120 110 100 90 80 70 60 50 40 30 20 10 0
f1 (ppm)

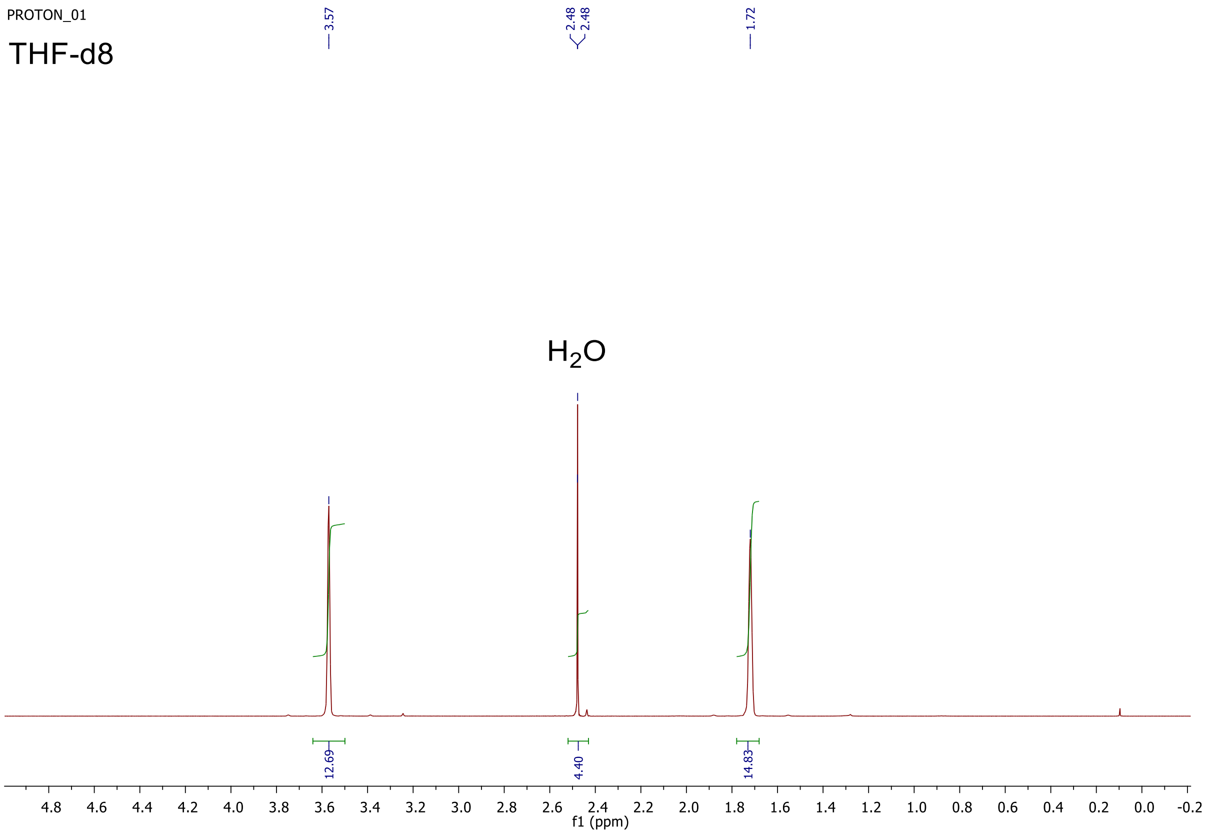
PROTON_01
THF-d8
3.57
2.48
2.48
1.72
H2O
12.69
4.40
14.83
f1 (ppm)

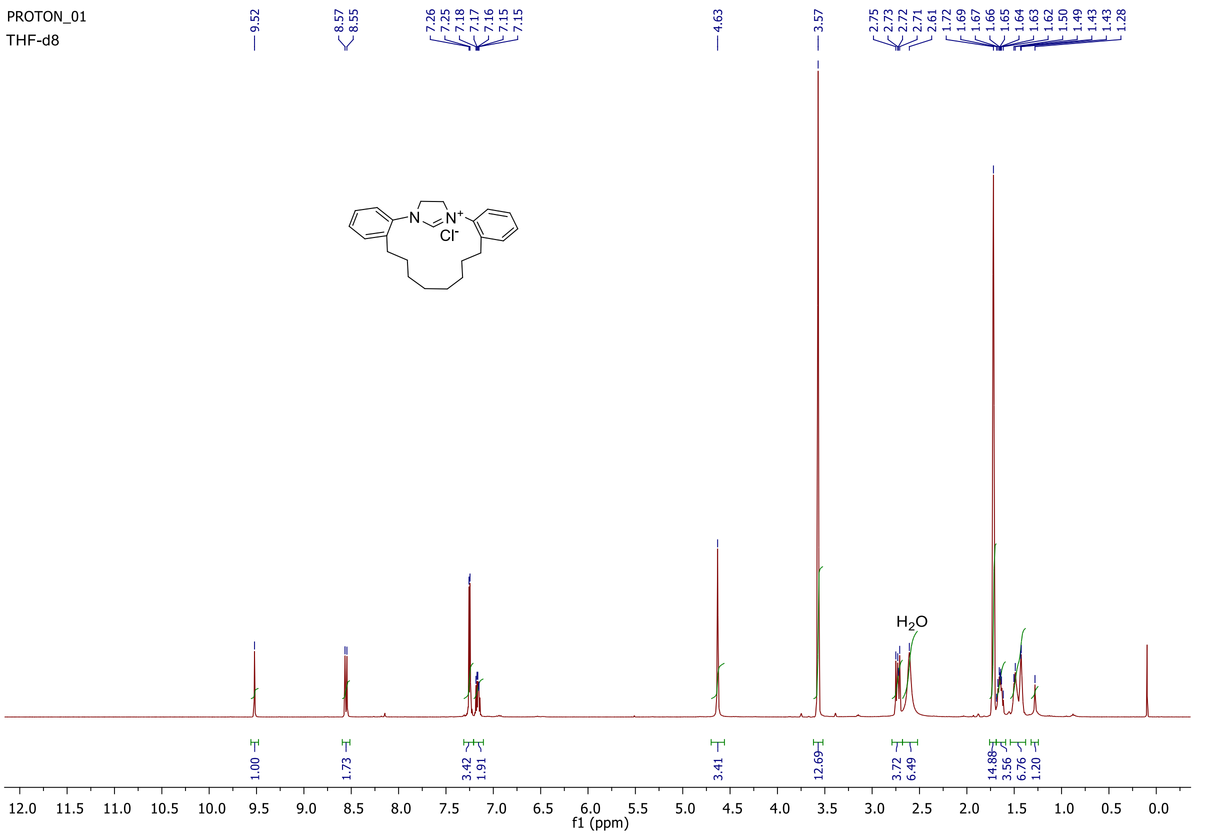
PROTON_01
THF-d8
9.52
8.57
8.55
4.63
3.57
Cl-
H2O
1.00
1.73
3.42
1.91
3.41
12.69
3.72
6.49
14.88
3.56
6.76
1.20
f1 (ppm)

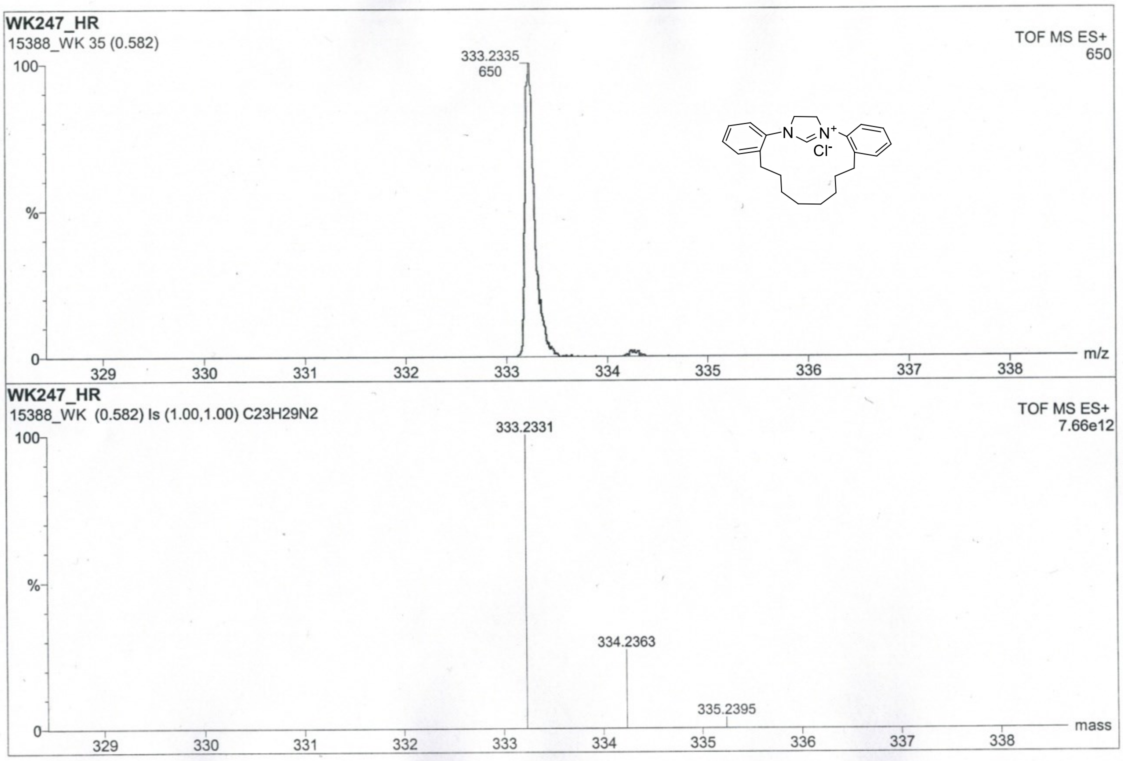

Elemental Composition Report

Page 1

Single Mass Analysis

Tolerance = 5.0 PPM / DBE: min = -0.5, max = 50.0

Monoisotopic Mass, Odd and Even Electron Ions
15 formula(e) evaluated with 1 results within limits (up to 30 closest results for each mass)

Minimum:				-0.5	
Maximum:		200.0	5.0	50.0	
Mass	Calc. Mass	mDa	PPM	DBE	Formula
333.2335	333.2331	0.4	1.3	10.5	C23 H29 N2

(*E*)-2^4,2^5-dihydro-2^1*H*-2(1,3)-imidazol-3-iuma-1,3(1,2)-dibenzenacycloundecaphan-2^3-ium tetrafluoroborate (6-BF_4)

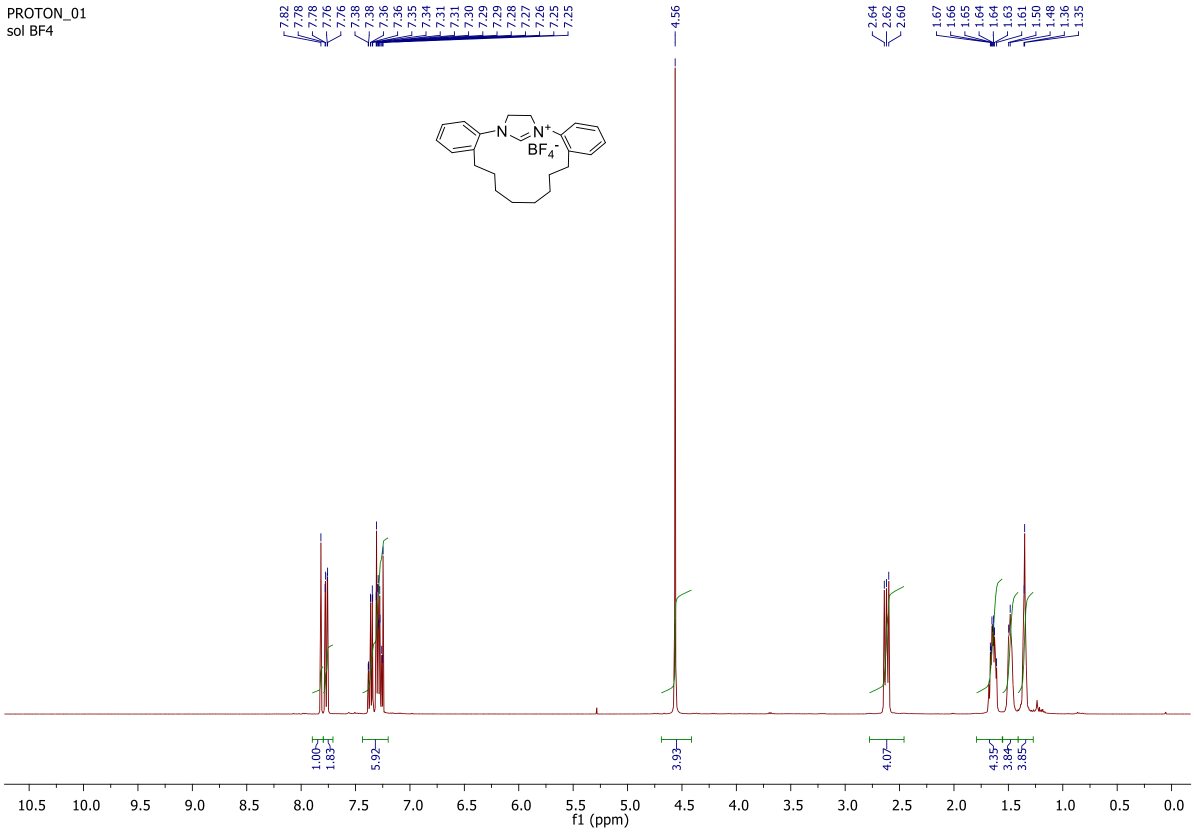

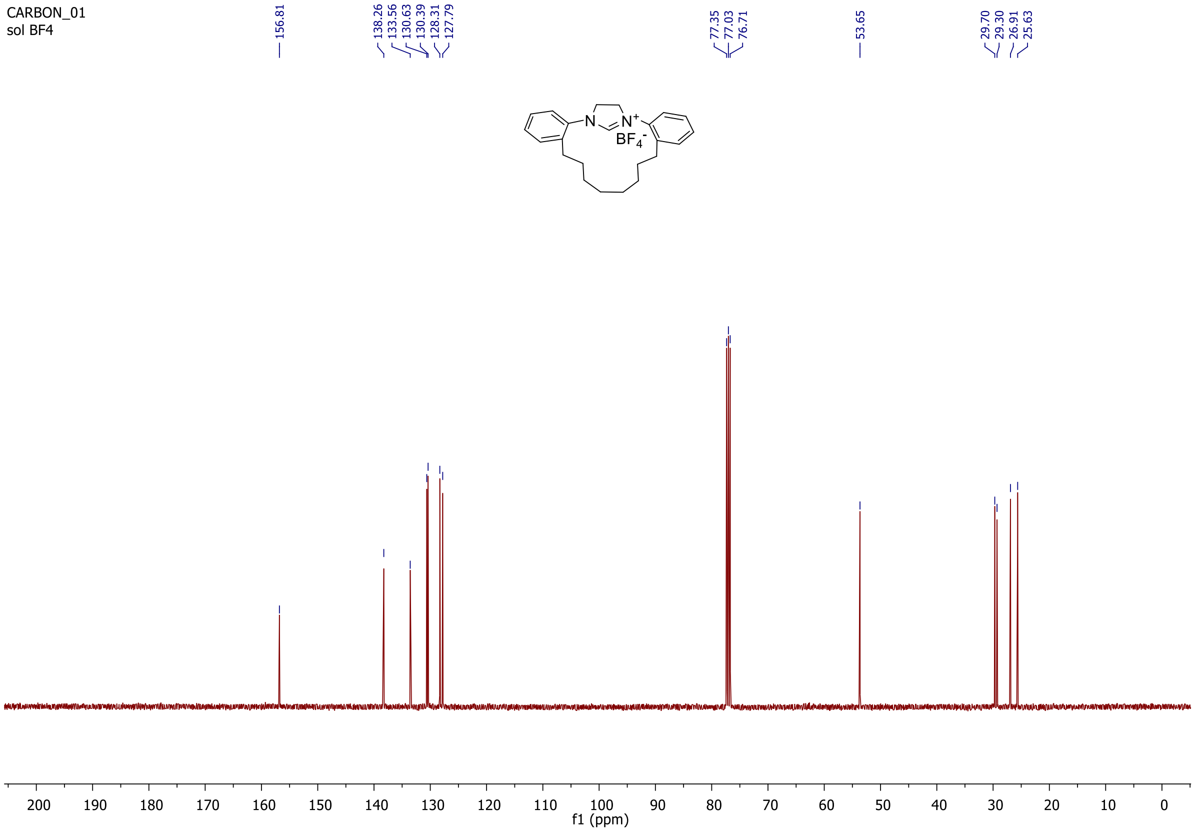

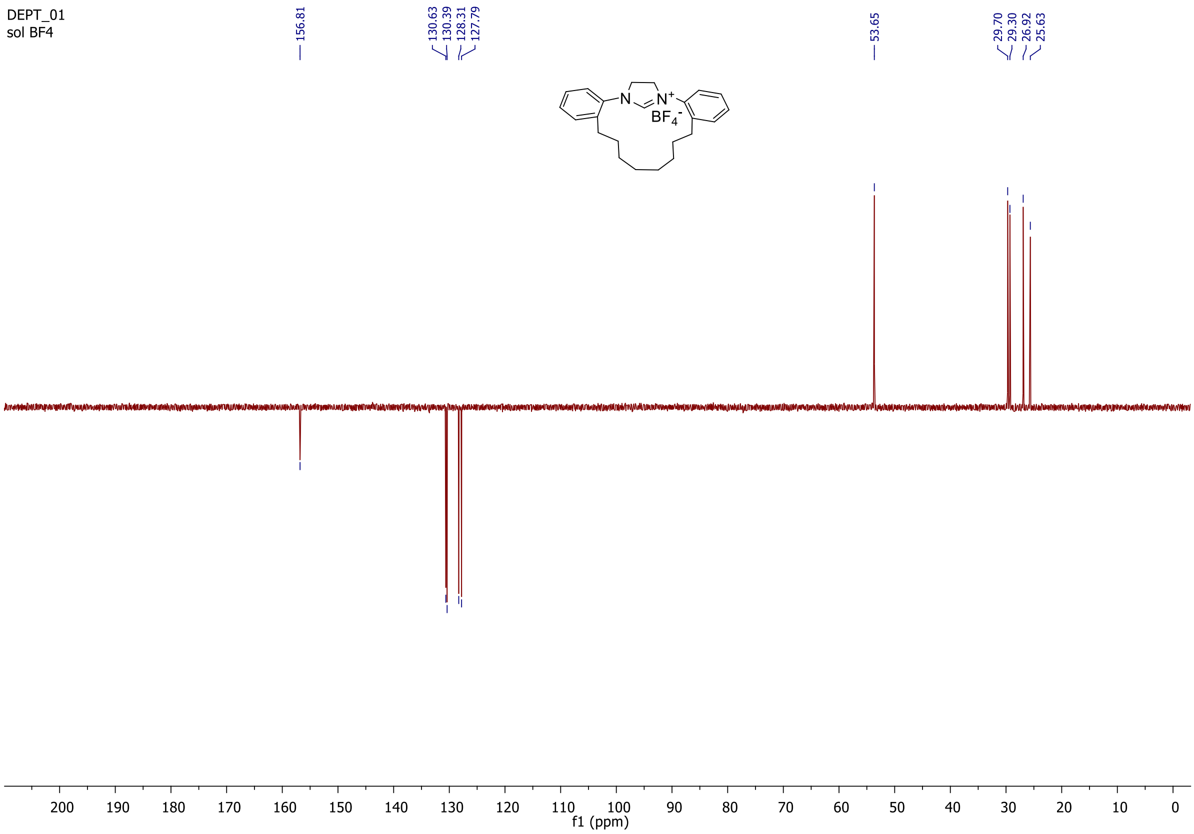
DEPT_01
sol BF4
156.81
130.63
130.39
128.31
127.79
53.65
29.70
29.30
26.92
25.63
N
N+
BF4-
200
190
180
170
160
150
140
130
120
110
100
90
80
70
60
50
40
30
20
10
0
f1 (ppm)

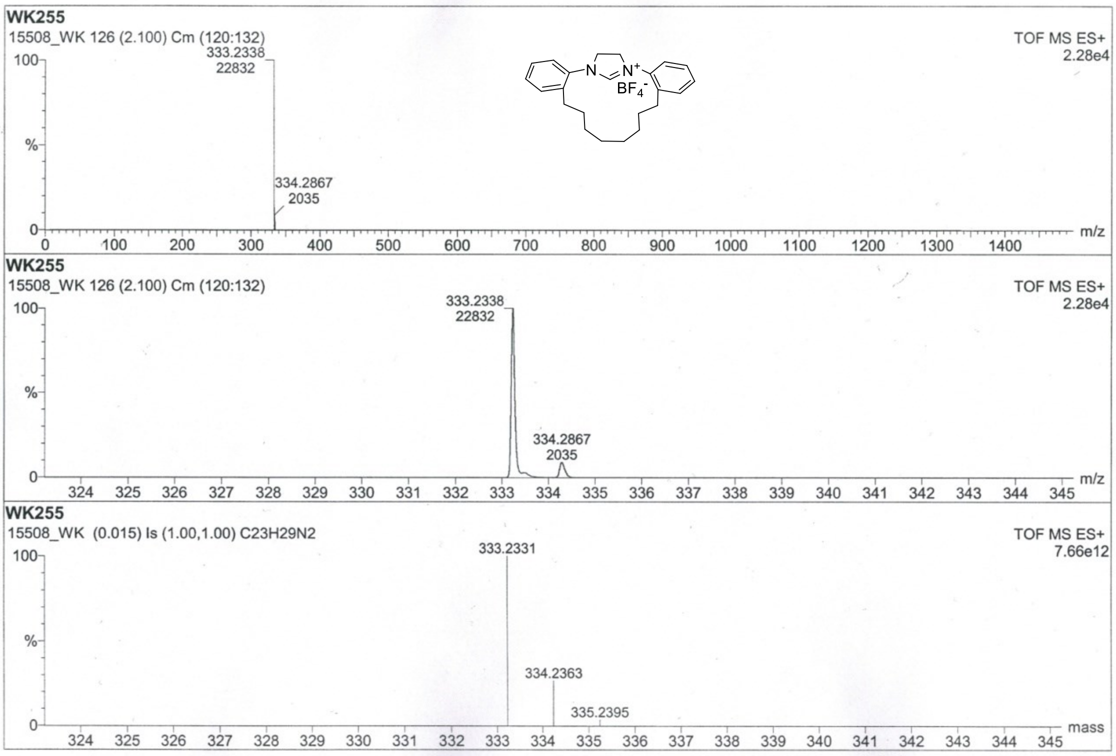
WK255
15508_WK 126 (2.100) Cm (120:132)
333.2338
22832
334.2867
2035
TOF MS ES+
2.28e4
m/z
WK255
15508_WK 126 (2.100) Cm (120:132)
333.2338
22832
334.2867
2035
TOF MS ES+
2.28e4
m/z
WK255
15508_WK (0.015) Is (1.00,1.00) C23H29N2
333.2331
334.2363
335.2395
TOF MS ES+
7.66e12
mass

Elemental Composition Report **Page 1**

Single Mass Analysis
Tolerance = 5.0 PPM / DBE: min = -0.5, max = 50.0

Monoisotopic Mass, Odd and Even Electron Ions
11 formula(e) evaluated with 1 results within limits (up to 30 closest results for each mass)

Mass	Calc. Mass	mDa	PPM	DBE	Formula
Minimum:				-0.5	
Maximum:		200.0	5.0	50.0	
333.2338	333.2331	0.8	2.3	10.5	C23 H29 N2

trans-**Ru6**

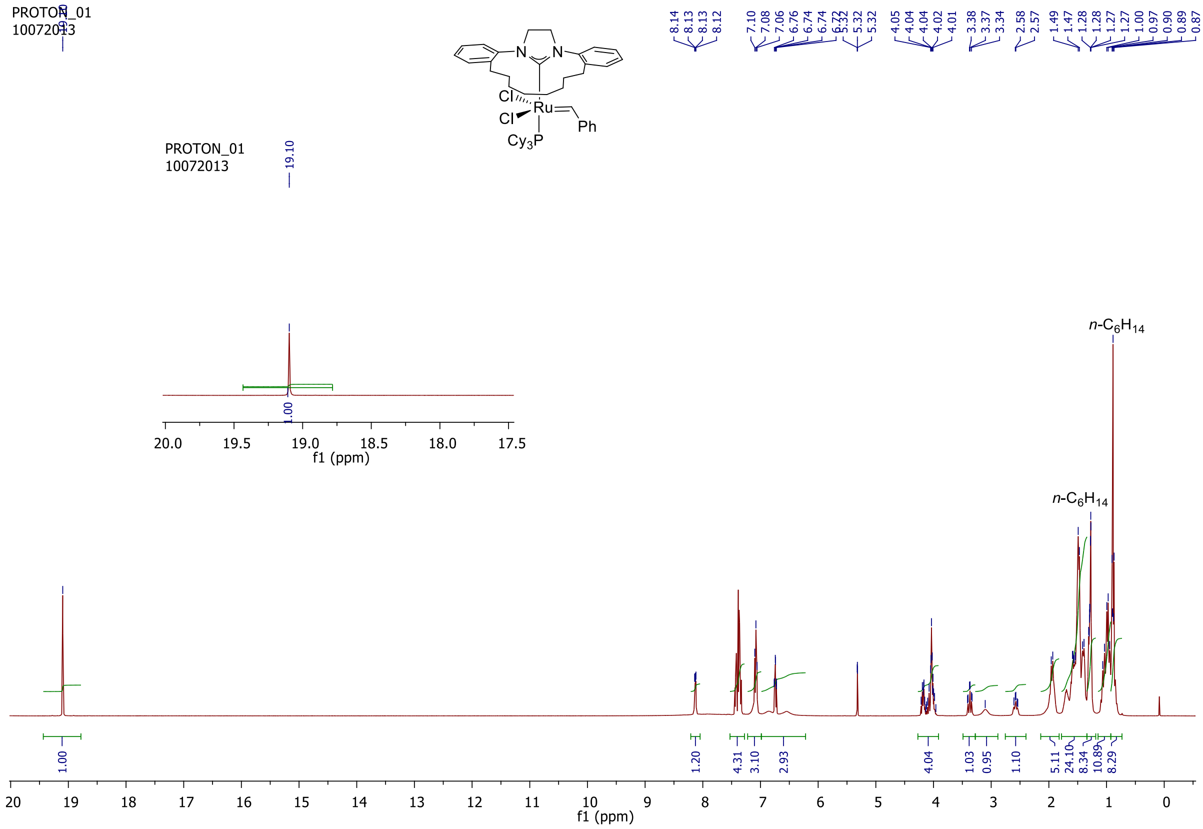

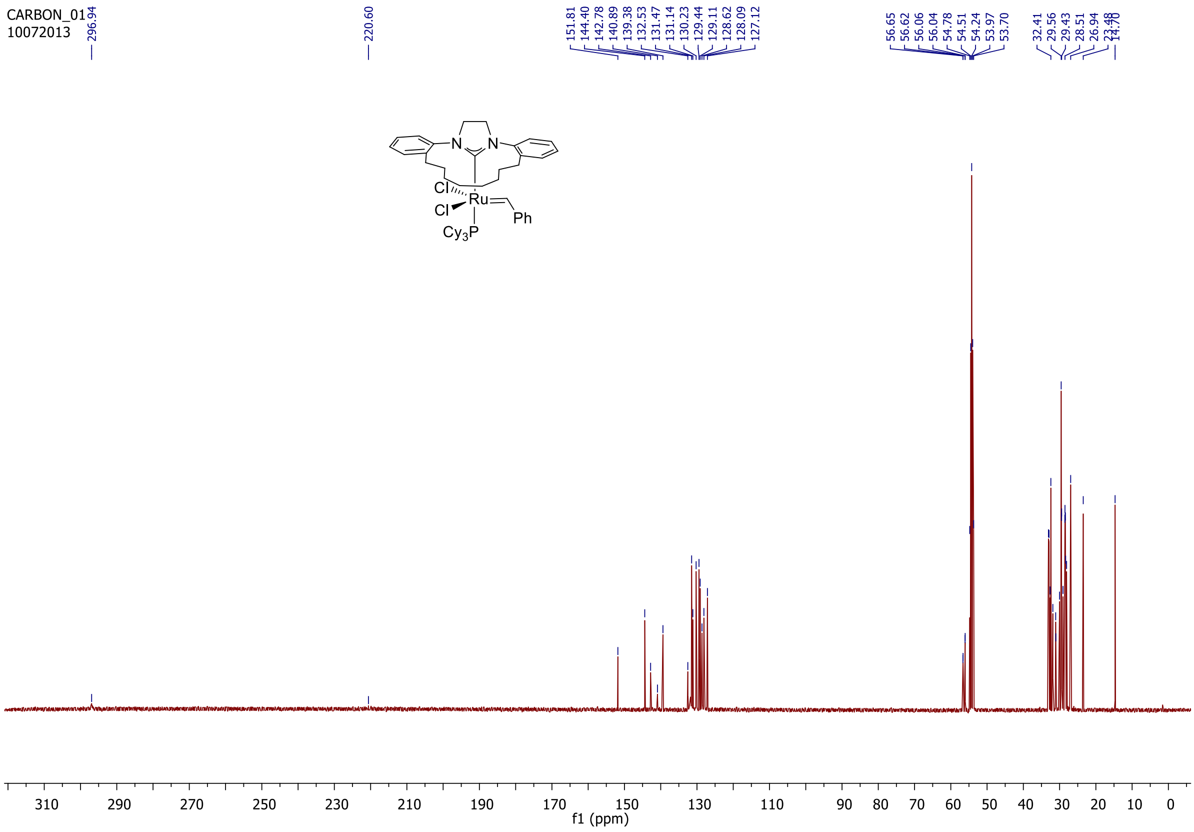
CARBON_01
10072013
f1 (ppm)
Cl
Ru
Ph
Cy3P
N

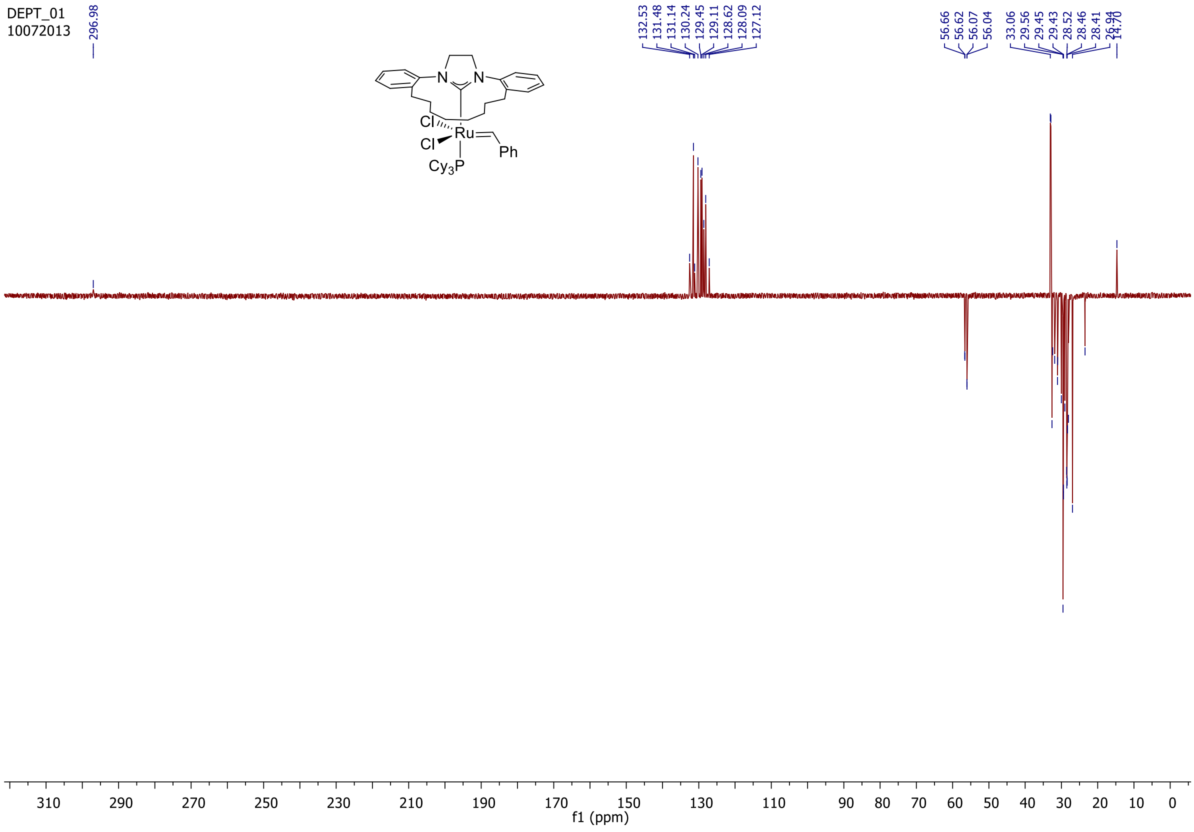
DEPT_01
10072013
f1 (ppm)
Cl
Ru
Ph
Cy3P
N

05072013
18.20
Sample Name: WKtrans402
Solvent: cd2cl2

Sample #4, Operator: grela_ADV

Relax. delay 1.000 sec
Acq. time 0.150 sec
Width 8012.8 Hz
2D Width 8012.8 Hz
8 repetitions
128 increments
OBSERVE H1, 399.9005888 MHz
DATA PROCESSING
Sq. sine bell 0.064 sec
F1 DATA PROCESSING
Sq. sine bell 0.016 sec
FT size 2048 x 4096
Total time 20 min

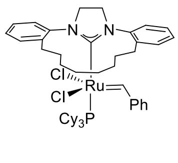

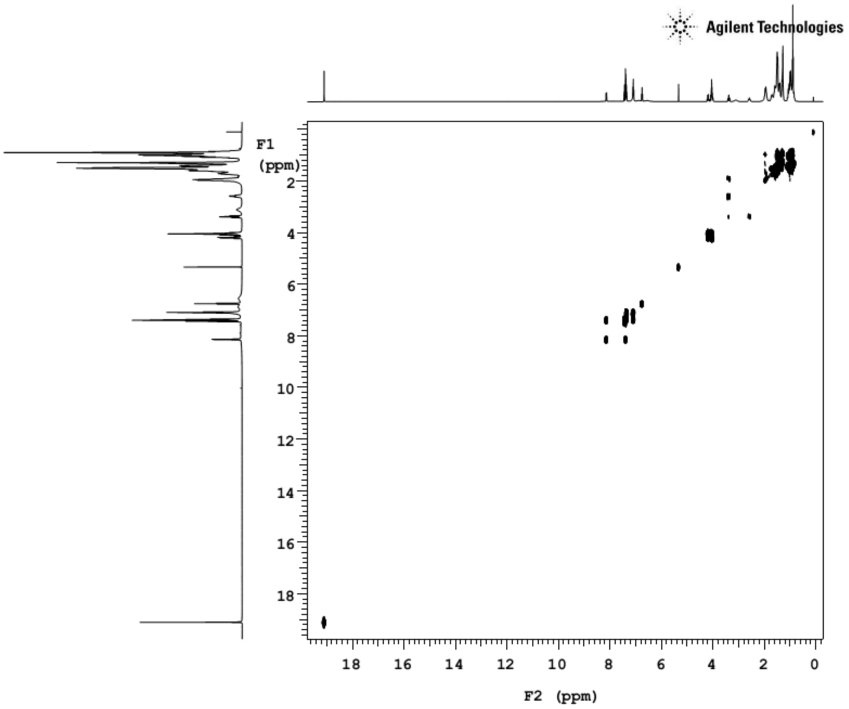

10072013
Sample Name: WKtransC8
Solvent: cd2cl2

Sample #2, Operator: grela_ADV

Relax. delay 1.000 sec
Acq. time 0.150 sec
Width 8012.8 Hz
2D Width 33181.3 Hz
16 repetitions
2 x 96 increments
OBSERVE H1, 399.9005888 MHz
DECOUPLE C13, 100.5706696 MHz
Power 36 dB
on during acquisition
off during delay
W40_OneNMR_W016 modulated
DATA PROCESSING
Gauss apodization 0.059 sec
F1 DATA PROCESSING
Gauss apodization 0.003 sec
FT size 2048 x 2048
Total time 1 hr, 1 min

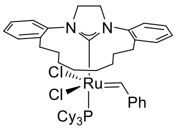

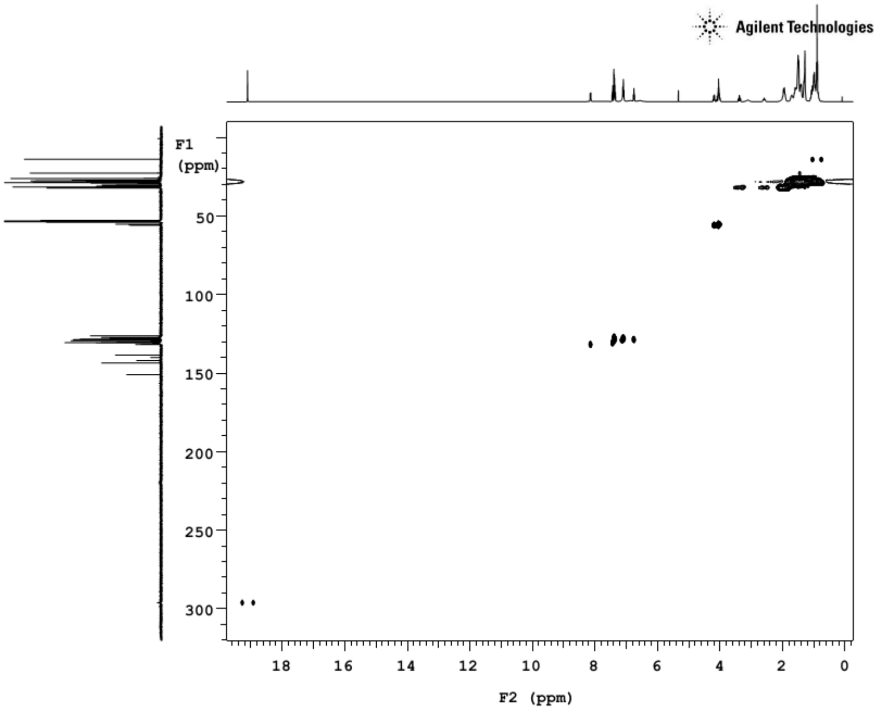

10072013
Sample Name: WKtransC8
Solvent: cd2cl2

Sample #2, Operator: grela_ADV

Relax. delay 1.000 sec
Acq. time 0.150 sec
Width 8012.8 Hz
2D Width 33684.2 Hz
8 repetitions
2 x 200 increments
OBSERVE H1, 399.9005888 MHz
DATA PROCESSING
Sq. sine bell 0.064 sec
F1 DATA PROCESSING
Gauss apodization 0.005 sec
FT size 2048 x 2048
Total time 1 hr, 6 min

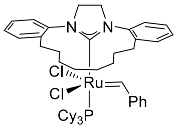

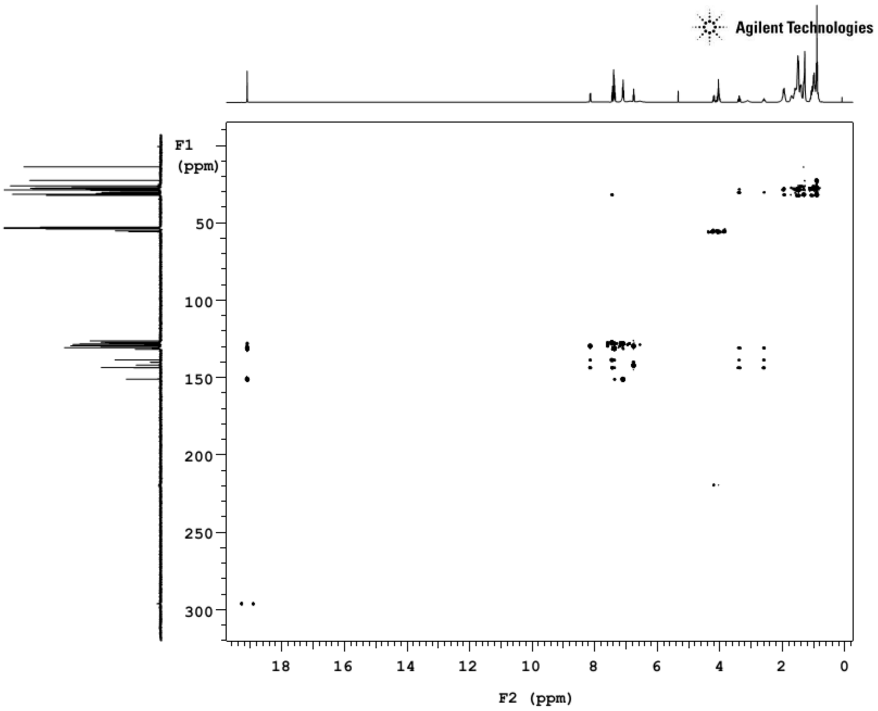

PHOSPHORUS_01
10072013

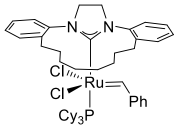

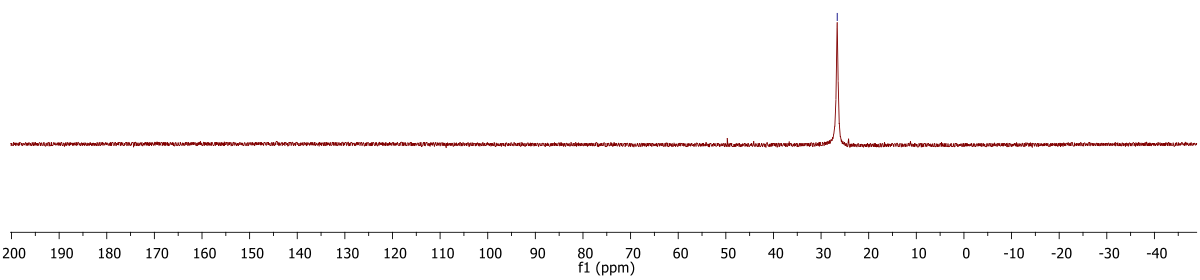

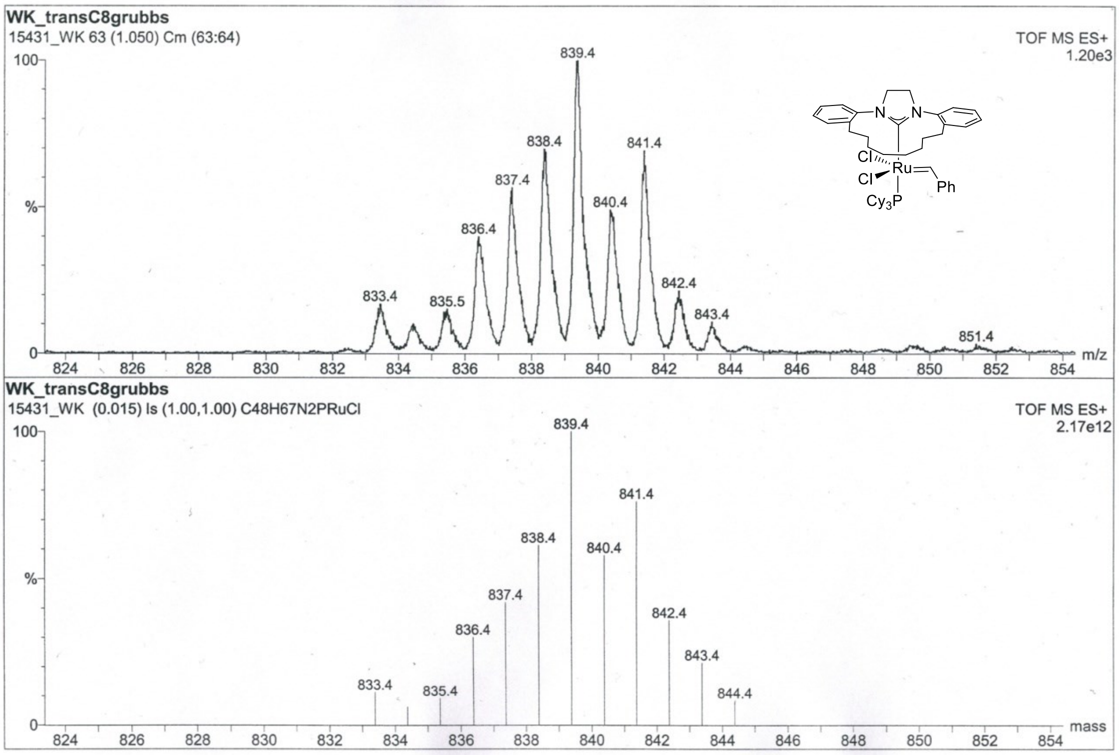

Elemental Composition Report

Page 1

Single Mass Analysis
Tolerance = 5.0 PPM / DBE: min = -0.5, max = 50.0

Monoisotopic Mass, Odd and Even Electron Ions
65 formula(e) evaluated with 2 results within limits (up to 30 closest results for each mass)

Minimum:				-0.5	
Maximum:		200.0	5.0	50.0	
Mass	Calc. Mass	mDa	PPM	DBE	Formula
839.3813	839.3777	3.6	4.3	12.0	C44 H70 N3 P2 Cl Ru
	839.3774	4.0	4.7	16.5	C48 H67 N2 P Cl Ru

cis-**Ru6**

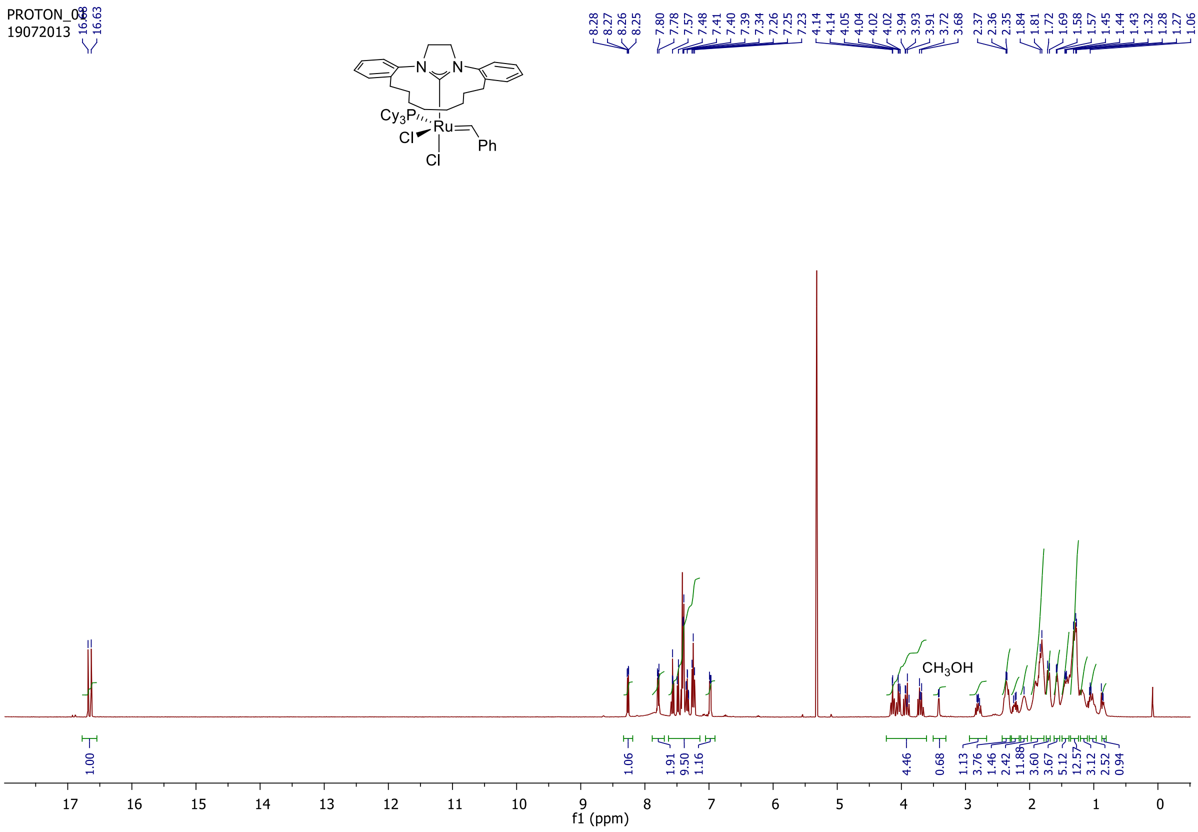

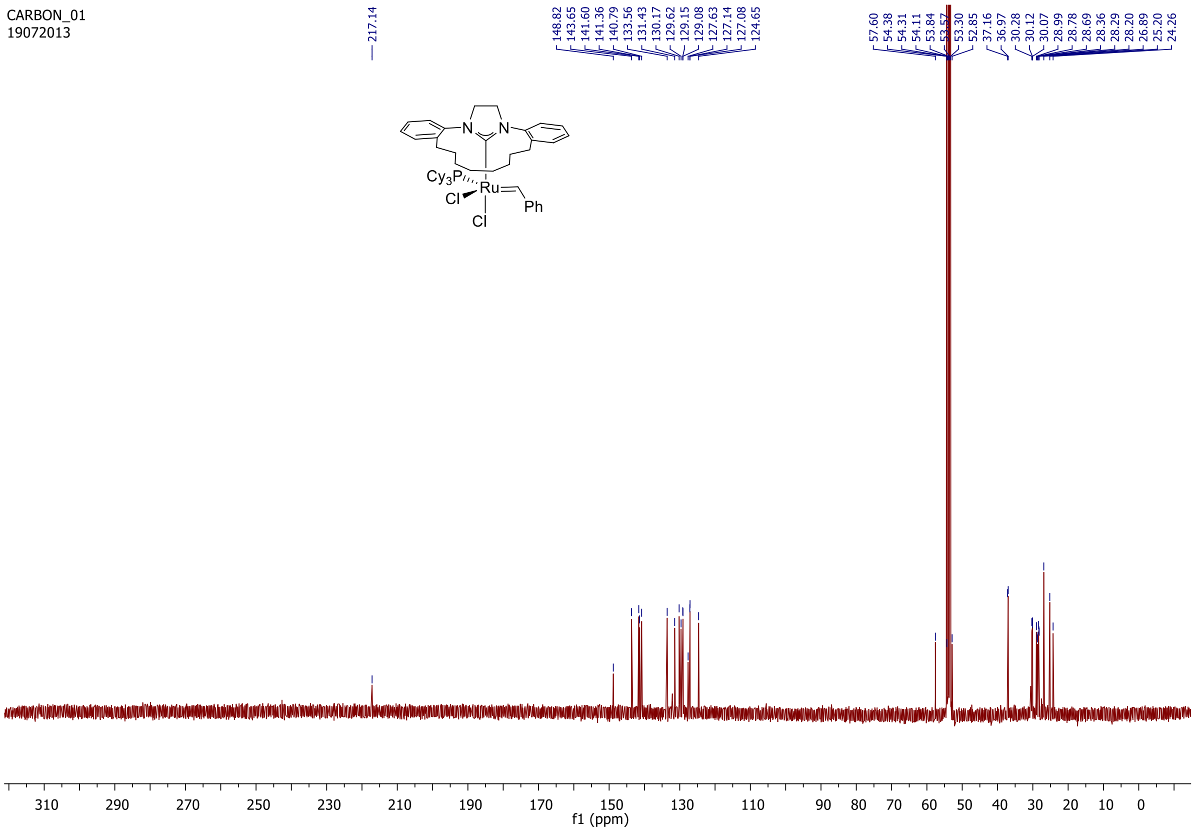

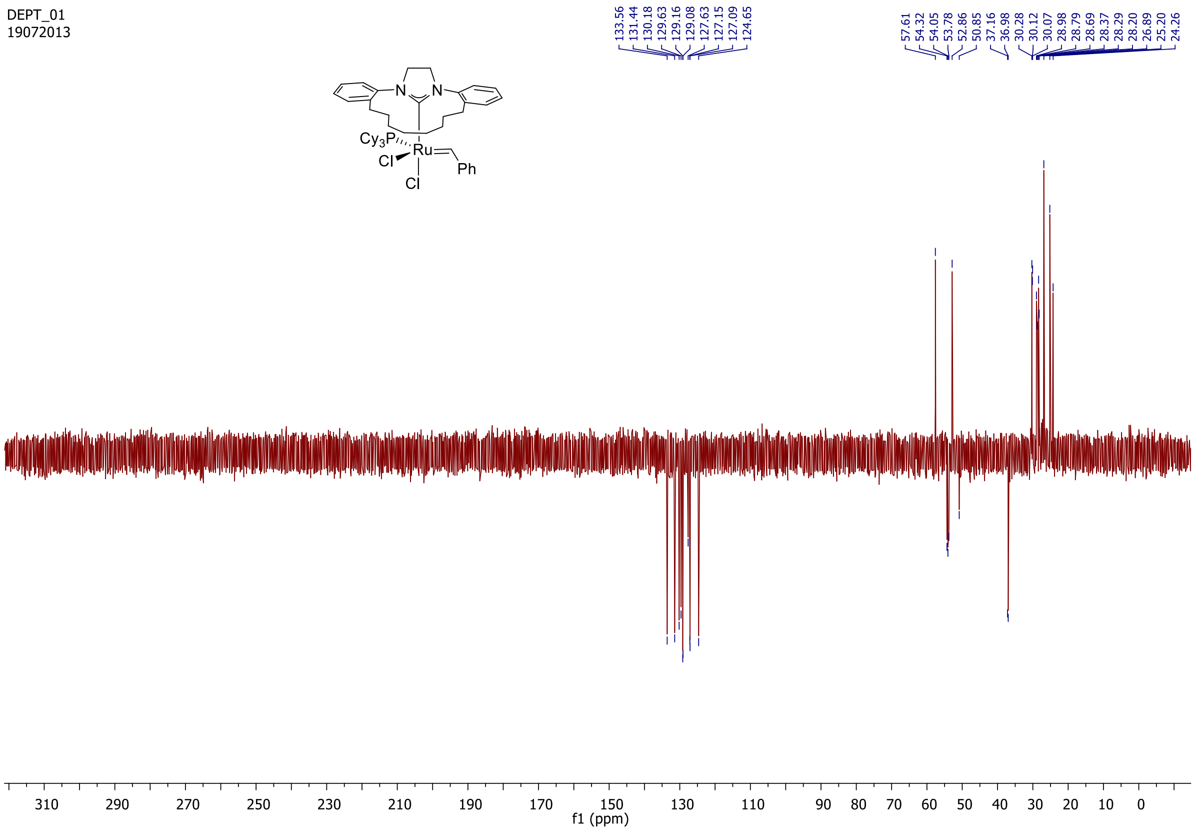
DEPT_01
19072013
133.56
131.44
130.18
129.63
129.16
129.08
127.63
127.15
127.09
124.65
57.61
54.32
54.05
53.78
52.86
50.85
37.16
36.98
30.28
30.12
30.07
28.98
28.79
28.69
28.37
28.29
28.20
26.89
25.20
24.26
Cy3P
Ru
Cl
Cl
Ph
N
N
f1 (ppm)

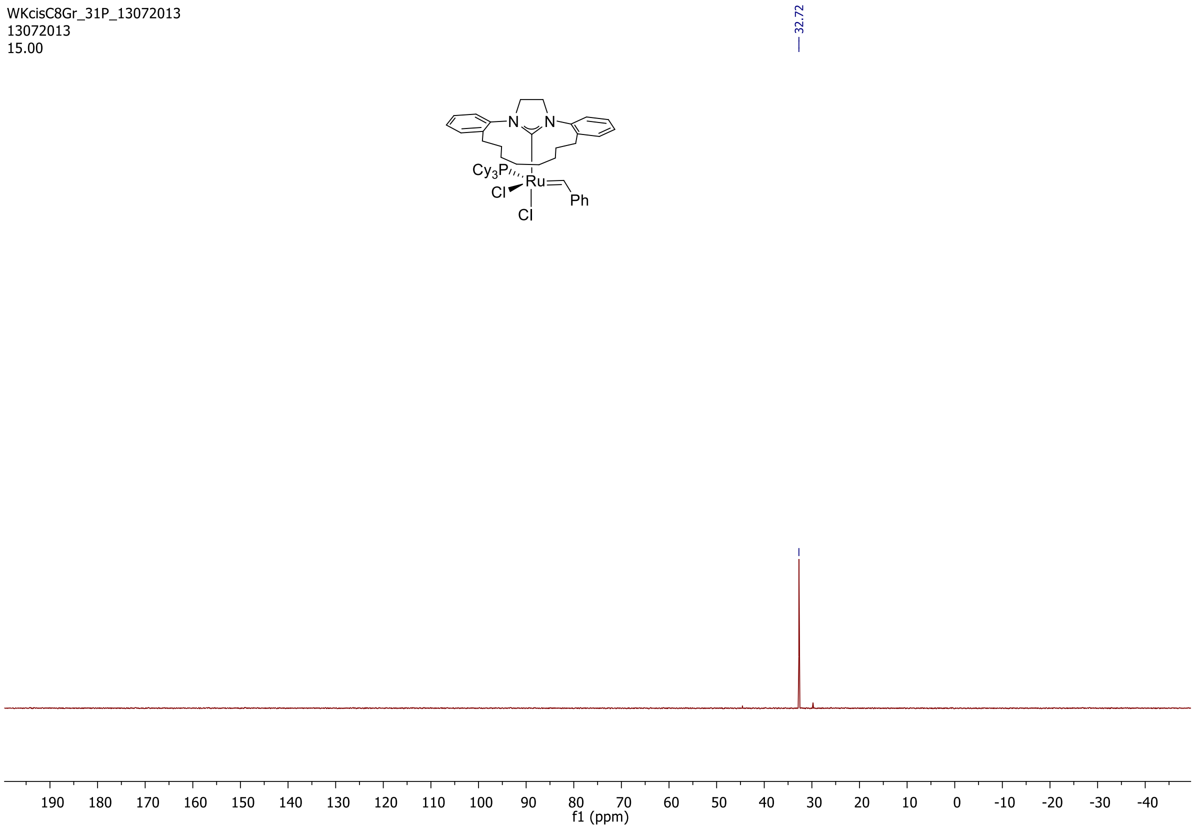
WKcisC8Gr_31P_13072013
13072013
15.00
32.72
Cy3P
Ru
Cl
Cl
Ph
N
N
f1 (ppm)

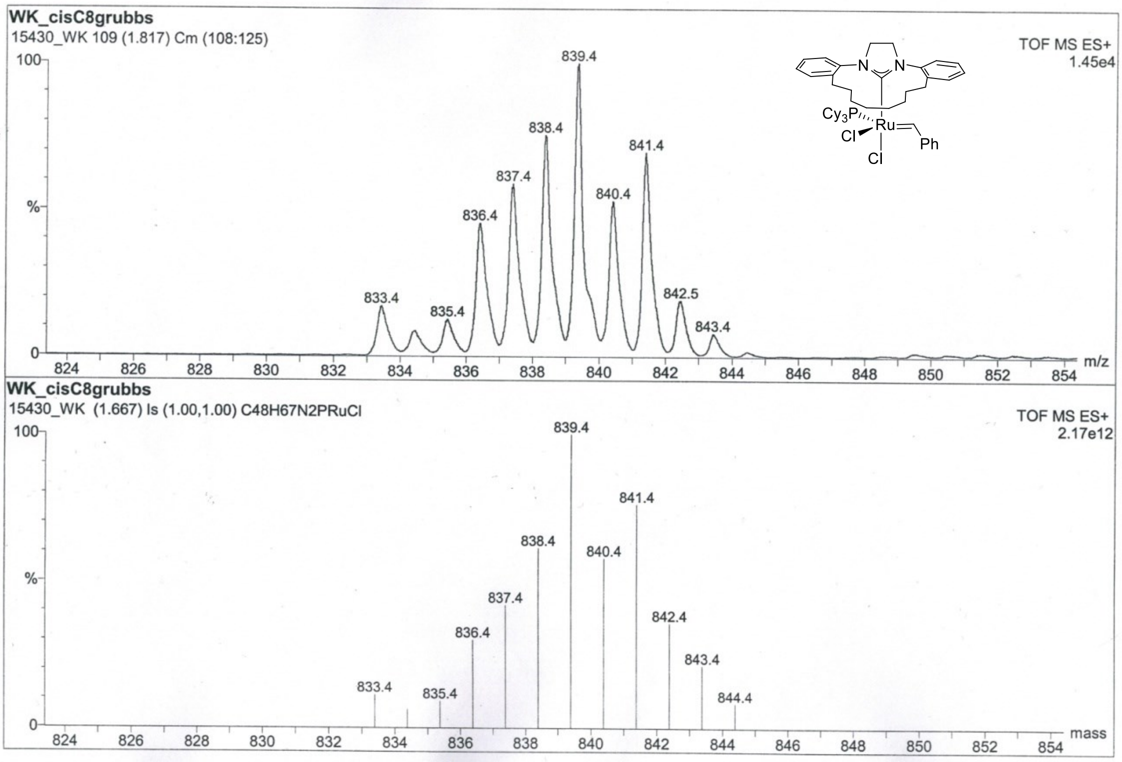

Elemental Composition Report

Page 1

Single Mass Analysis

Tolerance = 5.0 PPM / DBE: min = -0.5, max = 50.0

Monoisotopic Mass, Odd and Even Electron Ions
65 formula(e) evaluated with 2 results within limits (up to 30 closest results for each mass)

Minimum:				-0.5	
Maximum:		200.0	5.0	50.0	
Mass	Calc. Mass	mDa	PPM	DBE	Formula
839.3742	839.3774	-3.2	-3.8	16.5	C48 H67 N2 P Cl Ru
	839.3777	-3.5	-4.2	12.0	C44 H70 N3 P2 Cl Ru

Ru8

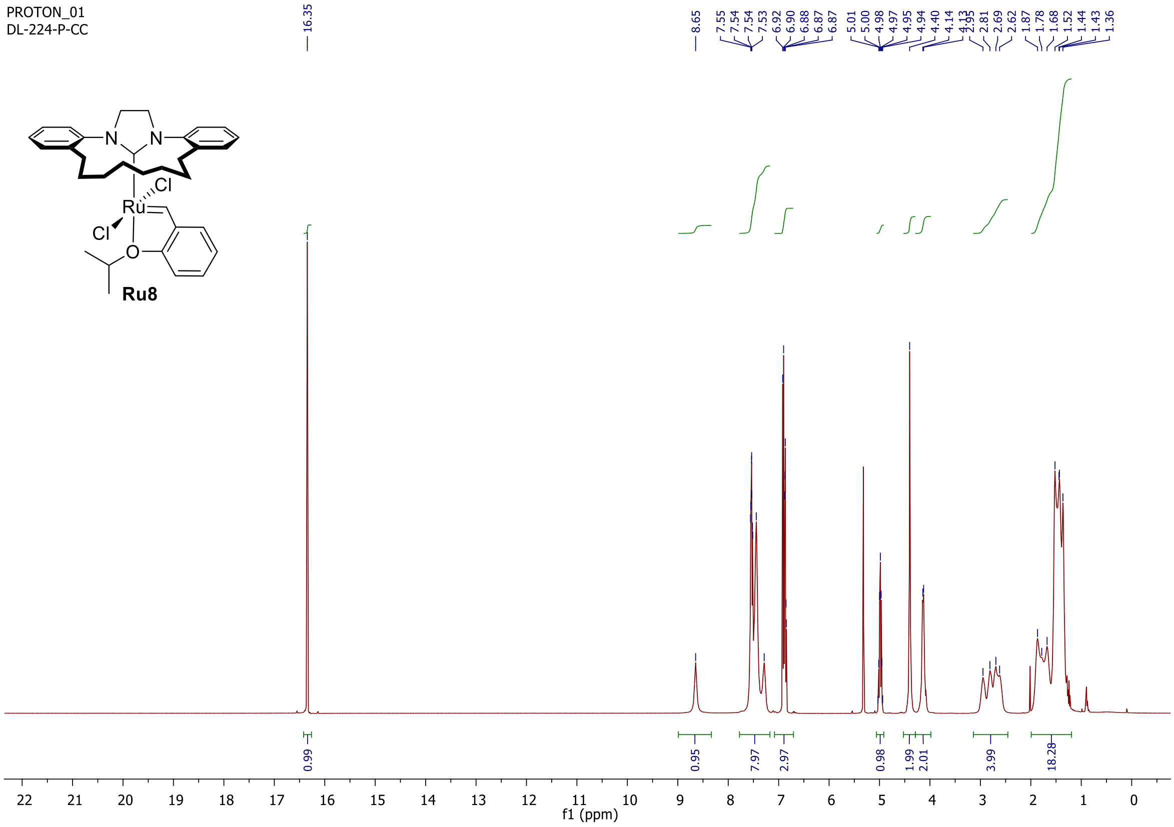
PROTON_01
DL-224-P-CC
16.35
8.65
7.55
7.54
7.54
7.53
6.92
6.90
6.88
6.87
6.87
5.01
5.00
4.98
4.97
4.95
4.94
4.40
4.14
2.95
2.81
2.69
2.62
1.87
1.78
1.68
1.52
1.44
1.43
1.36
Cl
Ru
Cl
O
N
N
Ru8
0.99
0.95
7.97
2.97
0.98
1.99
2.01
3.99
18.28
f1 (ppm)

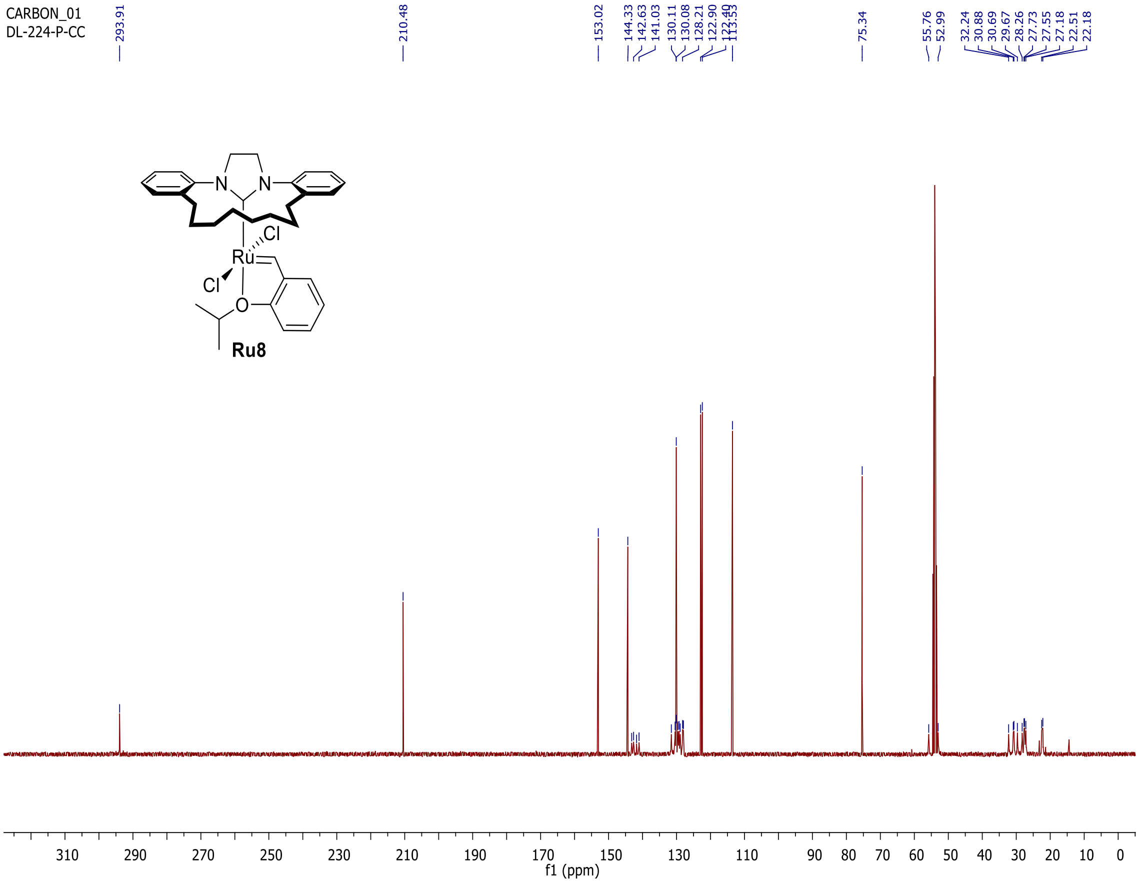
CARBON_01
DL-224-P-CC
293.91
210.48
153.02
144.33
142.63
141.03
130.11
130.08
128.21
122.90
113.53
75.34
55.76
52.99
32.24
30.88
30.69
29.67
28.26
27.73
27.55
27.18
22.51
22.18
Cl
Ru
Cl
O
N
N
Ru8
f1 (ppm)

1g

PROTON_01
DL-221-Substrate

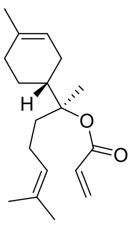

1g

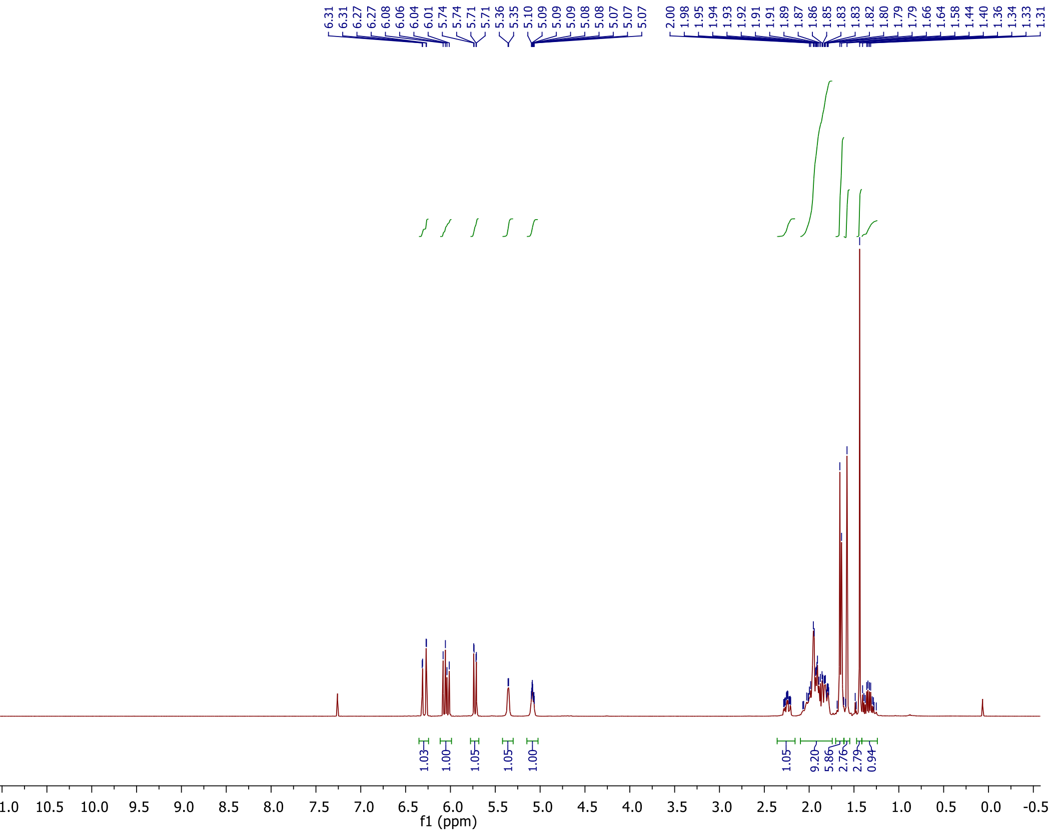

CARBON_01
DL-221-Substrate-C13

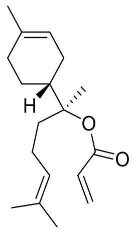

1g

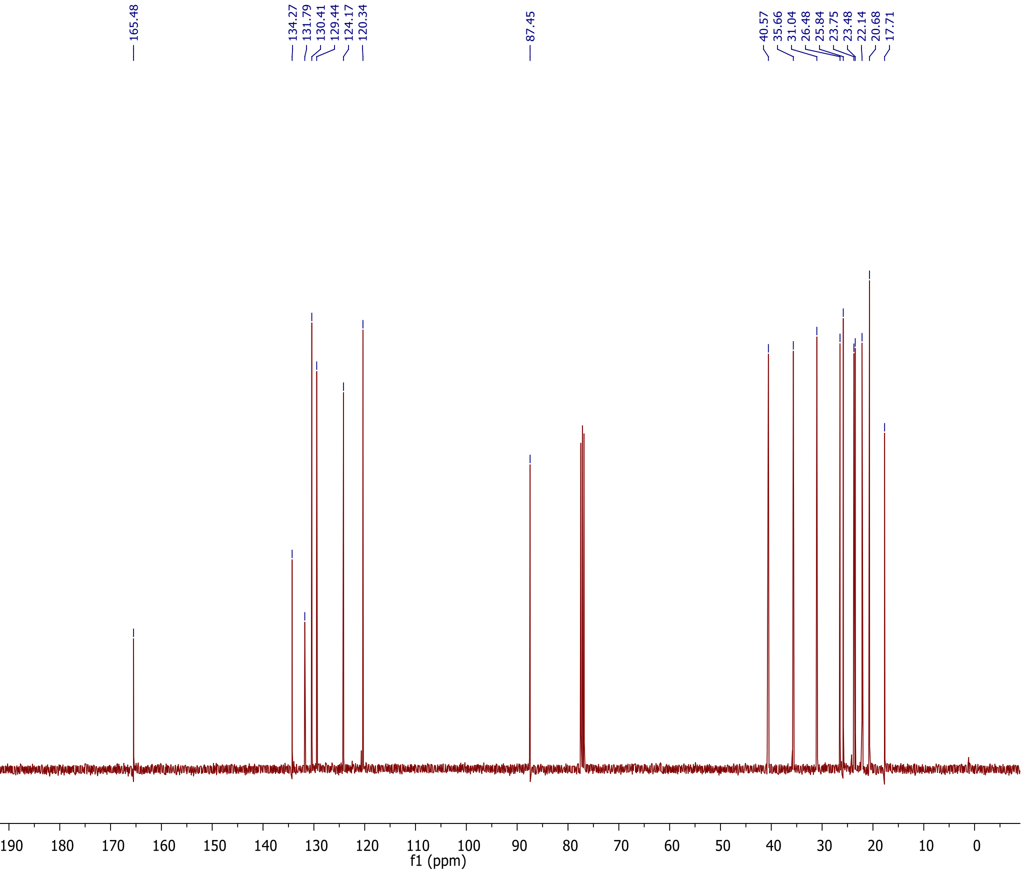

2g

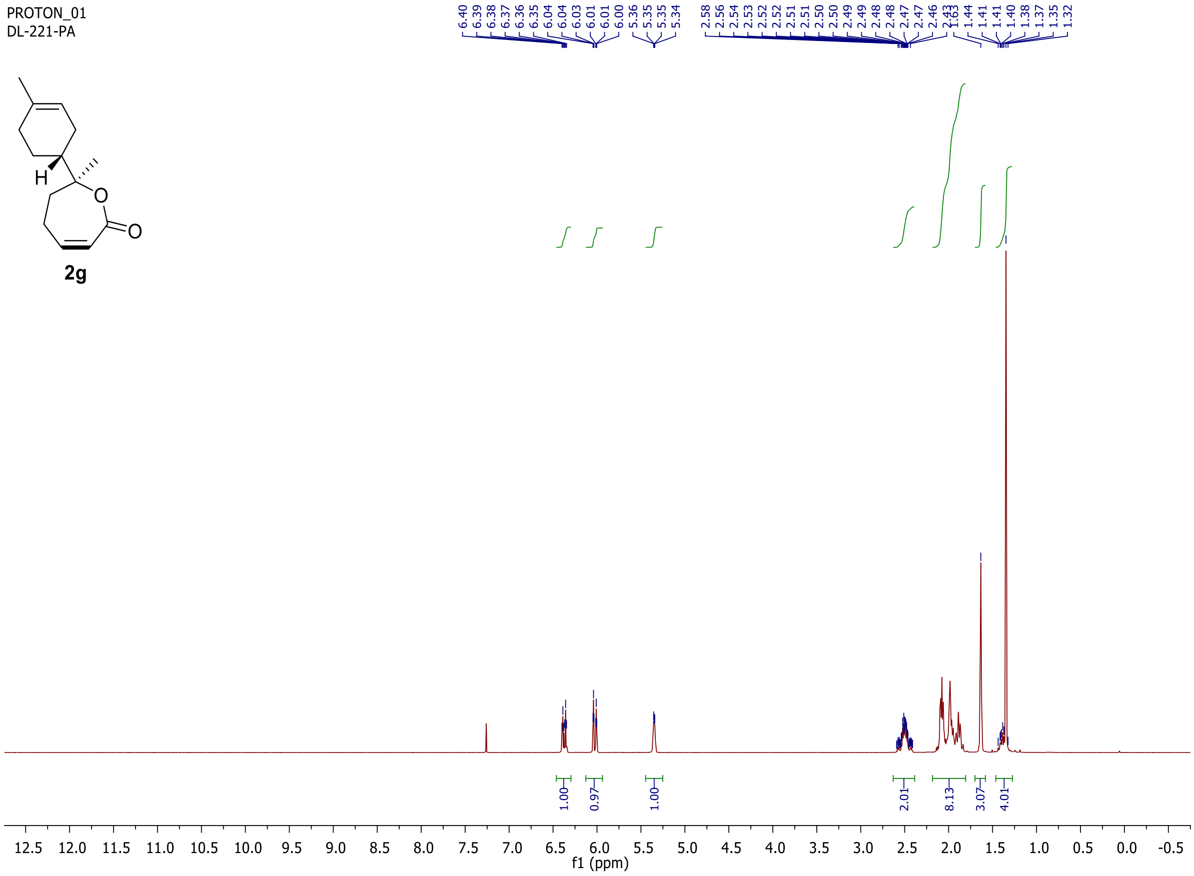
PROTON_01
DL-221-PA
6.40
6.39
6.38
6.37
6.36
6.35
6.04
6.03
6.01
6.01
6.00
5.36
5.35
5.35
5.34
2.58
2.56
2.54
2.53
2.52
2.52
2.51
2.51
2.50
2.50
2.49
2.49
2.48
2.48
2.47
2.47
2.46
1.44
1.41
1.41
1.40
1.38
1.37
1.35
1.32
H
O
O
2g
1.00
0.97
1.00
2.01
8.13
3.07
4.01
12.5
12.0
11.5
11.0
10.5
10.0
9.5
9.0
8.5
8.0
7.5
7.0
6.5
6.0
5.5
5.0
4.5
4.0
3.5
3.0
2.5
2.0
1.5
1.0
0.5
0.0
-0.5
f1 (ppm)

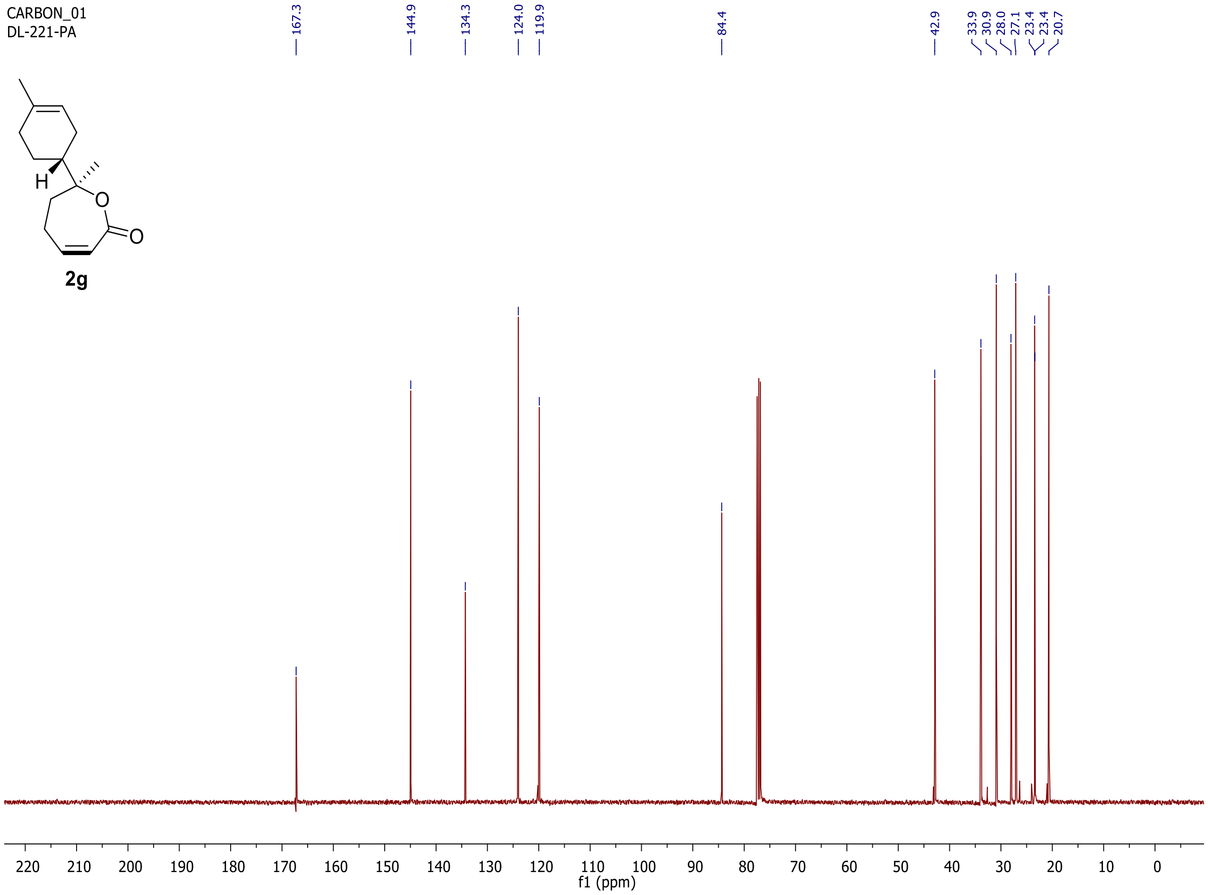
CARBON_01
DL-221-PA
167.3
144.9
134.3
124.0
119.9
84.4
42.9
33.9
30.9
28.0
27.1
23.4
23.4
20.7
H
O
O
2g
220
210
200
190
180
170
160
150
140
130
120
110
100
90
80
70
60
50
40
30
20
10
0
f1 (ppm)

9b

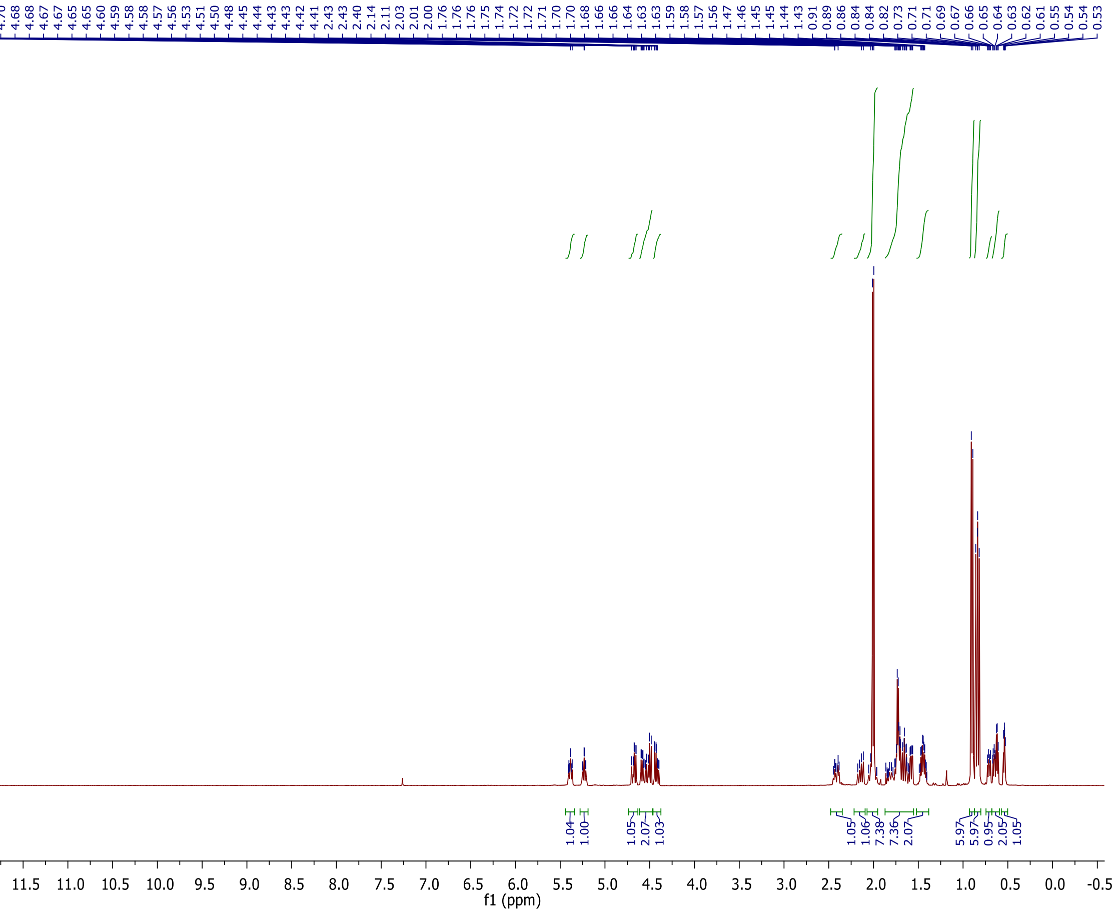

PROTON_01
DL-222-P
4.70 4.68 4.68 4.67 4.67 4.65 4.65 4.60 4.59 4.58 4.58 4.57 4.56 4.53 4.51 4.50 4.48 4.45 4.44 4.43 4.43 4.42 4.41 2.43 2.43 2.40 2.14 2.11 2.03 2.01 2.00 1.76 1.76 1.76 1.75 1.74 1.72 1.72 1.71 1.70 1.70 1.70 1.68 1.66 1.64 1.63 1.63 1.59 1.58 1.57 1.56 1.47 1.46 1.45 1.45 1.44 1.43 0.91 0.89 0.86 0.84 0.84 0.82 0.73 0.71 0.71 0.69 0.67 0.66 0.65 0.64 0.63 0.62 0.61 0.55 0.54 0.54 0.53
1.04 1.00 1.05 2.07 1.03 1.05 1.06 7.38 7.36 2.07 5.97 5.97 0.95 2.05 1.05
12.5 12.0 11.5 11.0 10.5 10.0 9.5 9.0 8.5 8.0 7.5 7.0 6.5 6.0 5.5 5.0 4.5 4.0 3.5 3.0 2.5 2.0 1.5 1.0 0.5 0.0 -0.5
f1 (ppm)

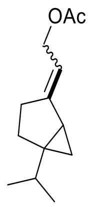

OAc

9b

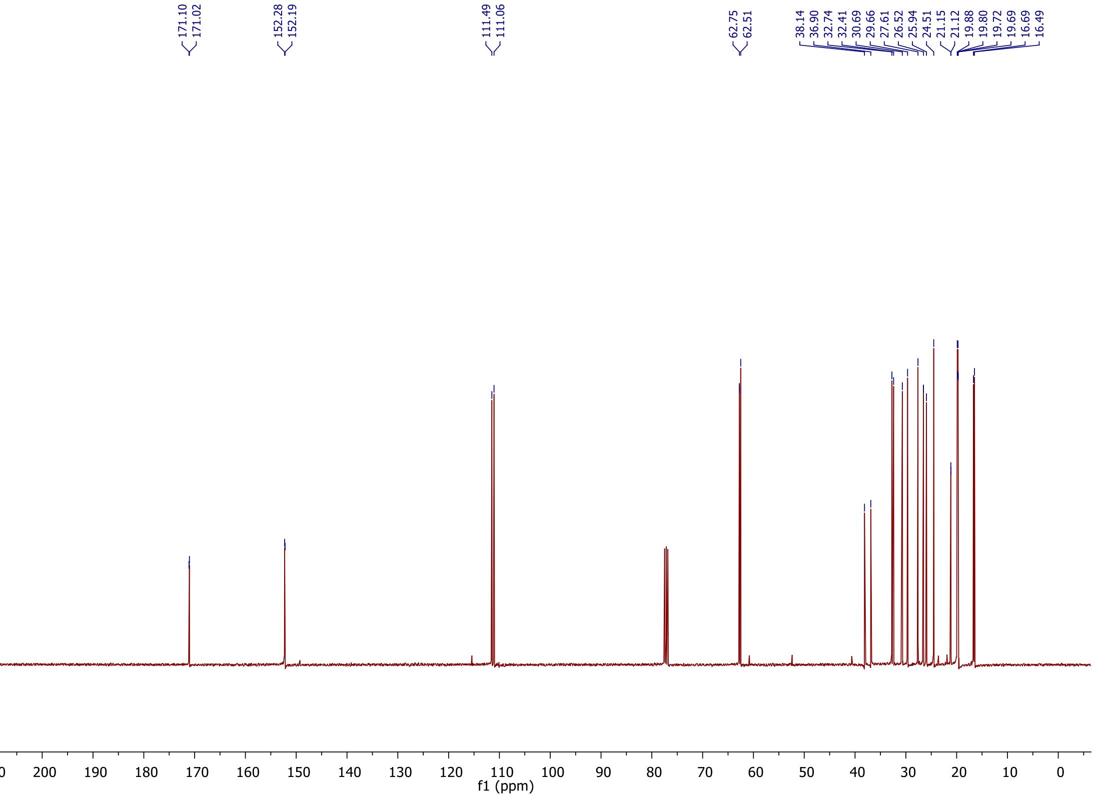

CARBON_01
DL-222-P
171.10 171.02 152.28 152.19 111.49 111.06 62.75 62.51 38.14 36.90 32.74 32.41 30.69 29.66 27.61 26.52 25.94 24.51 21.15 21.12 19.88 19.80 19.72 19.69 16.69 16.49
220 210 200 190 180 170 160 150 140 130 120 110 100 90 80 70 60 50 40 30 20 10 0
f1 (ppm)

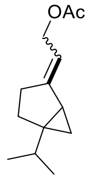

OAc

9b

15a

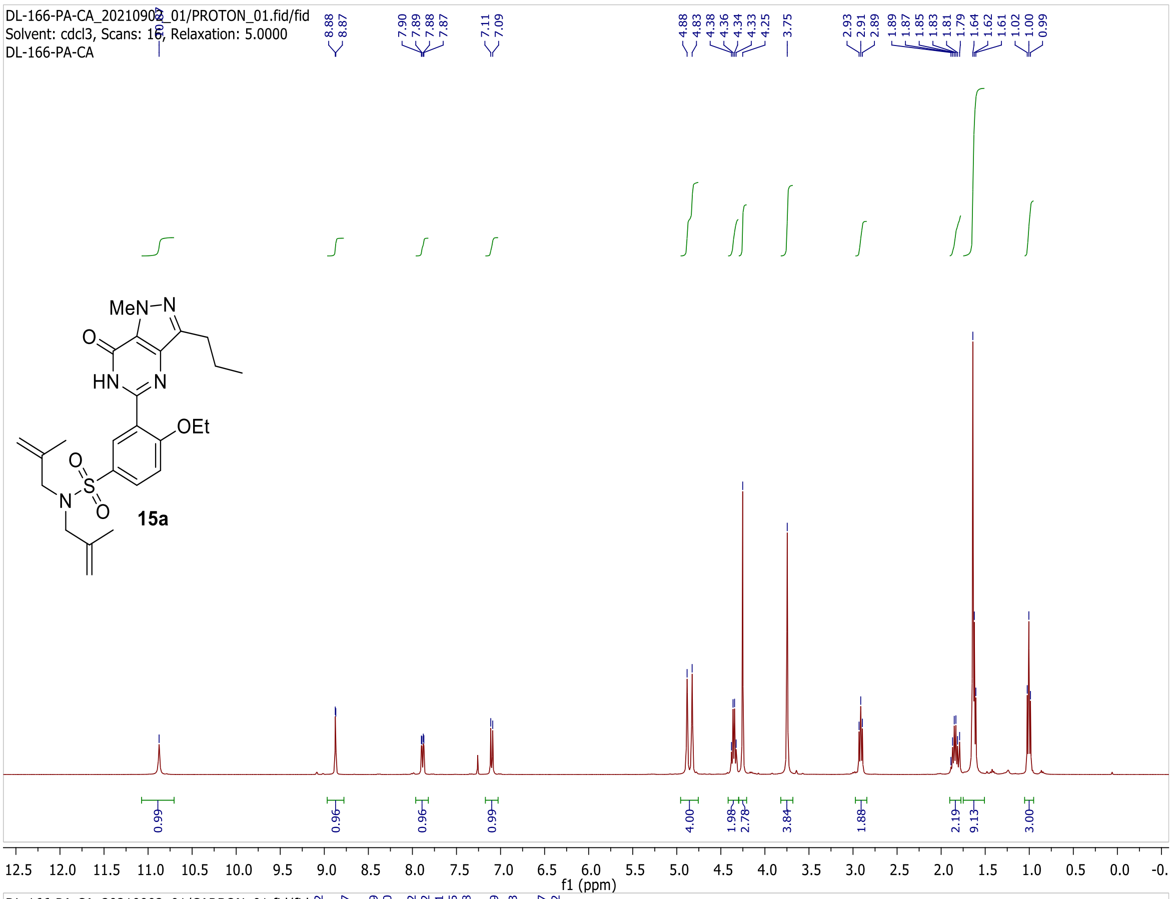

DL-166-PA-CA_20210902_01/PROTON_01.fid/fid
Solvent: cdcl3, Scans: 16, Relaxation: 5.0000
DL-166-PA-CA
8.88 8.87 7.90 7.89 7.88 7.87 7.11 7.09 4.88 4.83 4.38 4.36 4.34 4.33 4.25 3.75 2.93 2.91 2.89 1.89 1.87 1.85 1.83 1.81 1.79 1.64 1.62 1.61 1.02 1.00 0.99
0.99 0.96 0.96 0.99 4.00 1.98 2.78 3.84 1.88 2.19 9.13 3.00
MeN N O HN N OEt O S N O
15a
12.5 12.0 11.5 11.0 10.5 10.0 9.5 9.0 8.5 8.0 7.5 7.0 6.5 6.0 5.5 5.0 4.5 4.0 3.5 3.0 2.5 2.0 1.5 1.0 0.5 0.0
f1 (ppm)

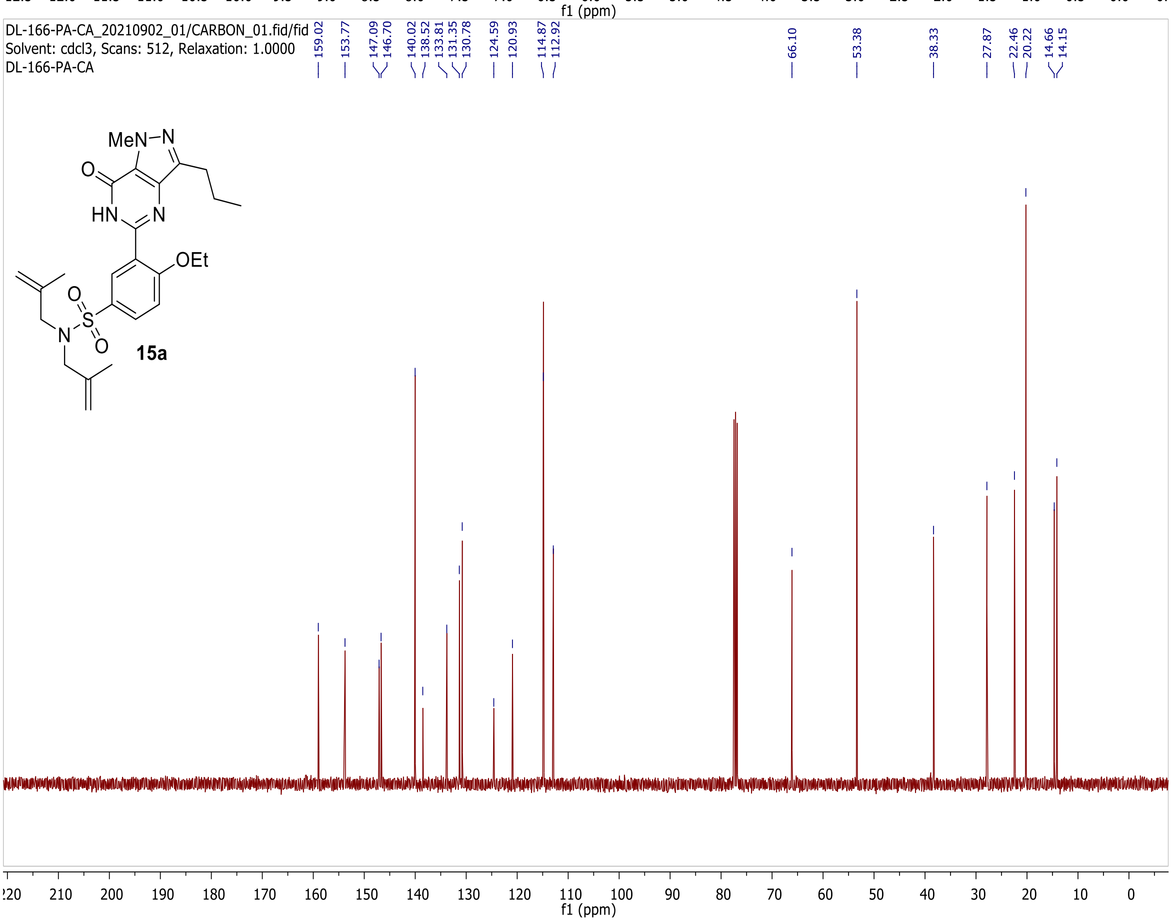

DL-166-PA-CA_20210902_01/CARBON_01.fid/fid
Solvent: cdcl3, Scans: 512, Relaxation: 1.0000
DL-166-PA-CA
159.02 153.77 147.09 146.70 140.02 138.52 133.81 131.35 130.78 124.59 120.93 114.87 112.92 66.10 53.38 38.33 27.87 22.46 20.22 14.66 14.15
MeN N O HN N OEt O S N O
15a
210 200 190 180 170 160 150 140 130 120 110 100 90 80 70 60 50 40 30 20 10 0
f1 (ppm)

15b

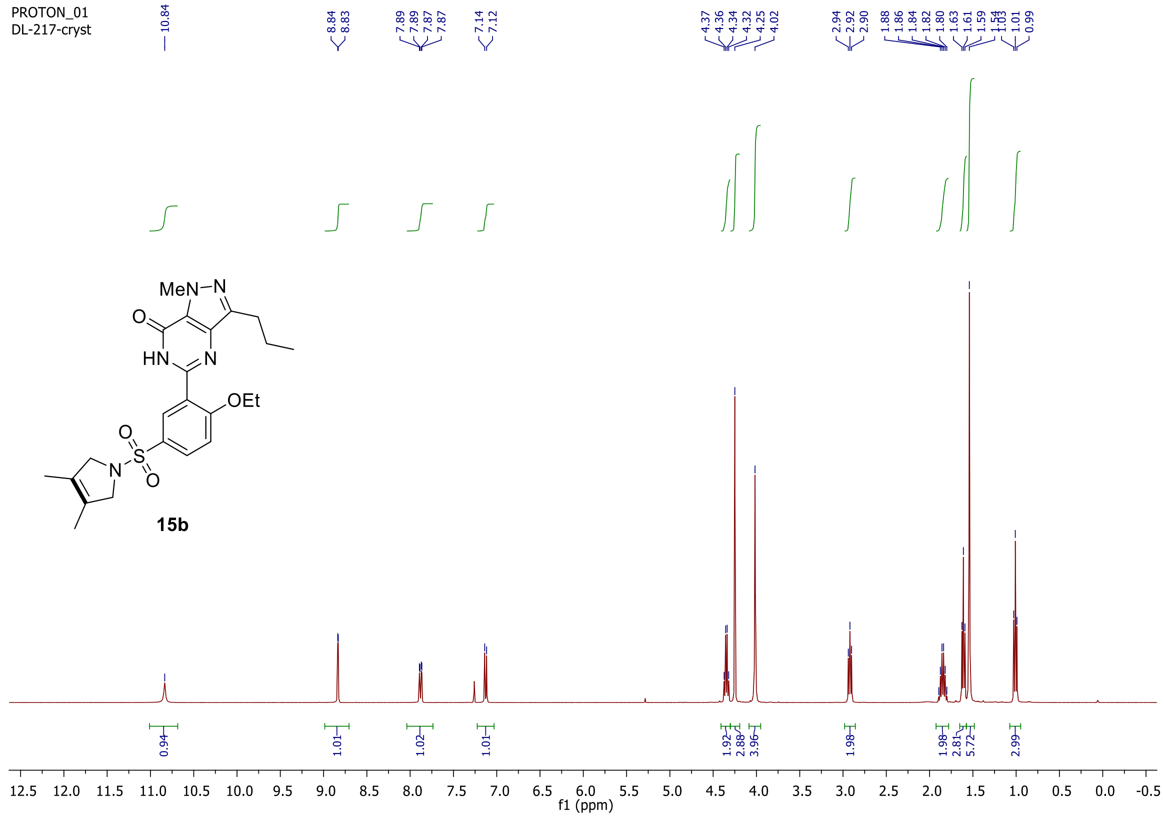
PROTON_01
DL-217-cryst
10.84
8.84
8.83
7.89
7.89
7.87
7.87
7.14
7.12
4.37
4.36
4.34
4.32
4.25
4.02
2.94
2.92
2.90
1.88
1.86
1.84
1.82
1.80
1.63
1.61
1.59
1.54
1.03
1.01
0.99
MeN
N
O
HN
N
OEt
O
S
N
O
15b
0.94
1.01
1.02
1.01
1.92
2.88
3.96
1.98
1.98
2.81
5.72
2.99
12.5 12.0 11.5 11.0 10.5 10.0 9.5 9.0 8.5 8.0 7.5 7.0 6.5 6.0 5.5 5.0 4.5 4.0 3.5 3.0 2.5 2.0 1.5 1.0 0.5 0.0 -0.5
f1 (ppm)

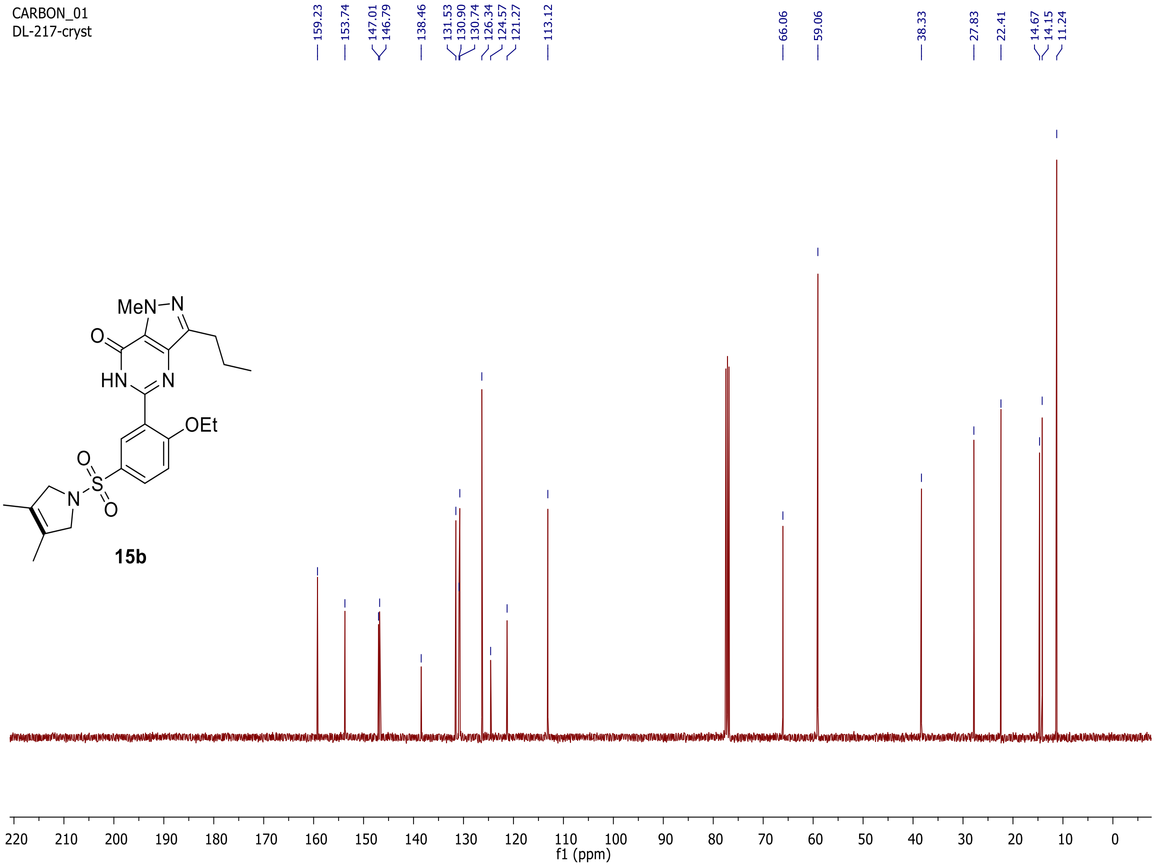
CARBON_01
DL-217-cryst
159.23
153.74
147.01
146.79
138.46
131.53
130.90
130.74
126.34
124.57
121.27
113.12
66.06
59.06
38.33
27.83
22.41
14.67
14.15
11.24
MeN
N
O
HN
N
OEt
O
S
N
O
15b
220 210 200 190 180 170 160 150 140 130 120 110 100 90 80 70 60 50 40 30 20 10 0
f1 (ppm)
